# The spatiotemporal evolution of compound impacts from lava flow and tephra fallout on buildings: lessons from the 2021 Tajogaite eruption (La Palma, Spain)

**DOI:** 10.1007/s00445-023-01700-w

**Published:** 2024-01-09

**Authors:** Sébastien Biass, María-Paz Reyes-Hardy, Christopher Gregg, Luigia Sara Di Maio, Lucia Dominguez, Corine Frischknecht, Costanza Bonadonna, Nemesio Perez

**Affiliations:** 1 Department of Earth Sciences, University of Geneva, Geneva, Switzerland; 2 Department of Geosciences, East Tennessee State University, Johnson City, USA; 3 Instituto Volcanológico de Canarias (INVOLCAN), San Cristóbal de La Laguna, Tenerife, Canary Islands Spain; 4 Instituto Tecnológico y de Energías Renovables (ITER), Granadilla de Abona, Tenerife, Canary Islands Spain

**Keywords:** Long-lasting hybrid eruptions, Tephra fallout, Lava flow, Compound impacts, Physical vulnerability

## Abstract

The simultaneous or sequential occurrence of several hazards—be they of natural or anthropogenic sources—can interact to produce unexpected *compound* hazards and impacts. Since success in responding to volcanic crises is often conditional on accurate identification of spatiotemporal patterns of hazard prior to an eruption, ignoring these interactions can lead to a misrepresentation or misinterpretation of the risk and, during emergencies, ineffective management priorities. The 2021 eruption of Tajogaite volcano on the island of La Palma, Canary Islands (Spain), was an 86 day-long hybrid explosive-effusive eruption that demonstrated the challenges of managing volcanic crises associated with the simultaneous emission of lava, tephra and volcanic gases. Here, we present the result of a small-scale impact assessment conducted during three-field deployments to investigate how tephra fallout and lava flow inundation interacted to cause compound physical impact on buildings. The study area was a neighbourhood of 30 buildings exposed to tephra fallout during the entire eruption and by a late-stage, short-lived lava flow. Observations highlight, on one hand, the influence of clean-up operations and rainfall on the impact of tephra fallout and, on the other hand, the importance of the dynamics of lava flow emplacement in controlling impact mechanisms. Overall, results provide an evidence-based insight into impact sequences when two primary hazards are produced simultaneously and demonstrate the importance of considering this aspect when implementing risk mitigation strategies for future long-lasting, hybrid explosive-effusive eruptions in urban environments.

**Supplementary Information:**

The online version contains supplementary material available at 10.1007/s00445-023-01700-w.

## Introduction

Understanding the relationship between various hazards is required to integrate the assessment of single phenomena into a multi-hazard framework that not only superimposes individual events (Gill and Malamud 2014; Sandri et al. 2014) but also considers their interactions in time and space (Catto and Dowdy 2021). With climate change and global warming, a growing number of interconnections is being recognised between natural and technological hazards and anthropogenic processes (AghaKouchak et al. 2018; Fink and Ajibade 2022), outlining various natures of interactions. Gill and Malamud (2016) suggest three types of interaction relationships (e.g. triggering, increased probability and catalysis/impedance) taking place within a network of possible interaction, which, amongst recent various terminologies being proposed, can be referred to as hazard chains, cascades, domino effects, compound hazards or coupled events (Pescaroli and Alexander 2018; Van Westen and Greiving 2017). We adopt here the generic definition of Fink and Ajibade (2022) where “compound” refers to the accumulating effect of multiple hazards without implying any causal connection amongst them.

Volcanic unrest and eruptions are inherently multi-hazard systems, so interactions of different hazard-related processes are common during volcanic crises. A range of hazardous processes can simultaneously affect the surrounding populations and environment, with proximal hazards (e.g. volcanic ballistic projectiles) affecting the vicinity of the vent and medial (e.g. lava flows, pyroclastic density currents, lahars) and distal (e.g. ash dispersal and fallout) impacts ranging from the proximal vent area to a few to hundreds of kilometres (e.g. Baxter et al. 2017; Biass et al. 2014, 2017; Deligne et al. 2022; Hayes et al. 2019; Jenkins et al. 2017; Kaneko et al. 2016; Kilgour et al. 2010; Neal et al. 2018; Spence et al. 1996). Hazards causatively connected to the eruption include interactions between two volcanic processes (e.g. cushioning of ballistic impacts from tephra fallout deposit during the 2014 eruption of Ontake, Japan; Kaneko et al. 2016; Williams et al. 2019) or between volcanic and external processes (e.g. simultaneous occurrence of the climatic phase of the 1991 Pinatubo eruption and landfall of typhoon Yunya on Luzon island; Lin et al. 2020; Oswalt et al. 1996). In the volcanological literature, the interest for compound hazards has primarily been dominated by the remobilisation of loose pyroclastic material originating from primary hazards (i.e. fall deposits or pyroclastic density currents) by water (typically rainfall or glacier melting; Barclay et al. 2007; Coppola et al. 2020; de Bélizal et al. 2013; Mead and Magill 2017; Miller et al. 2022) to generate secondary hazards (i.e. lahars). Although justified by the magnitude of their impacts and their persistence in time (Brown et al. 2017), impacts of eruptions can also result from a multitude of different interactions which, due to the empirical and opportunistic nature of post-event impact assessments (post-EIA), have received less attention (Blong 1984; Deligne et al. 2022).

The Sep. 19–Dec. 13, 2021, VEI 3 Tajogaite eruption on the island of La Palma (Spain) is the most recent example of a long-lasting, hybrid explosive-effusive eruption. Here, *hybrid eruption* refers to the simultaneous emission of lava flows and tephra caused by gas segregation in the shallow plumbing systems (Pioli et al. 2009; Schipper et al. 2013), with hazards able to interact and evolve to produce compound impacts. By *long lasting*, we refer to a sustained emission of material lasting for weeks to months. Throughout the 86 day-long eruptive sequence, lava flows dominated the physical impacts, destroying ~ 3000 buildings, impacting ~ 1000 hectares of farmland and plantations and 92 km of roads and causing a major disturbance to the general accessibility in the island (Carracedo et al. 2022; Rey et al. 2023). Tephra fallout and gas emissions concurrently occurred during the 14 weeks of eruption, mainly causing widespread disturbances that complicated the management of the crisis. Tephra fallout further impacted agricultural activities, caused the closure of airports in the Canary Islands and impacted buildings and towns unaffected by lava flows. Clean-up operations were setup to mitigate impacts of tephra fallout, allowing residents of the evacuated areas to temporarily access the restricted zones under strict supervision from members of security organisations designated by PEVOLCA (i.e. *Plan Especial de Protección Civil y Atención de Emergencias por riesgo volcánico en la Comunidad Autónoma de Canarias*). At the same time, gas emissions and laze (i.e. acidic gas caused by lava entering the ocean) resulted in locally high concentrations of PM10, SO_2_, and CO_2_ that, amongst other impacts on surrounding communities (e.g. closing of schools, confinements), sporadically prevented access to restricted areas and clean-up operations.

**Fig. 1:**
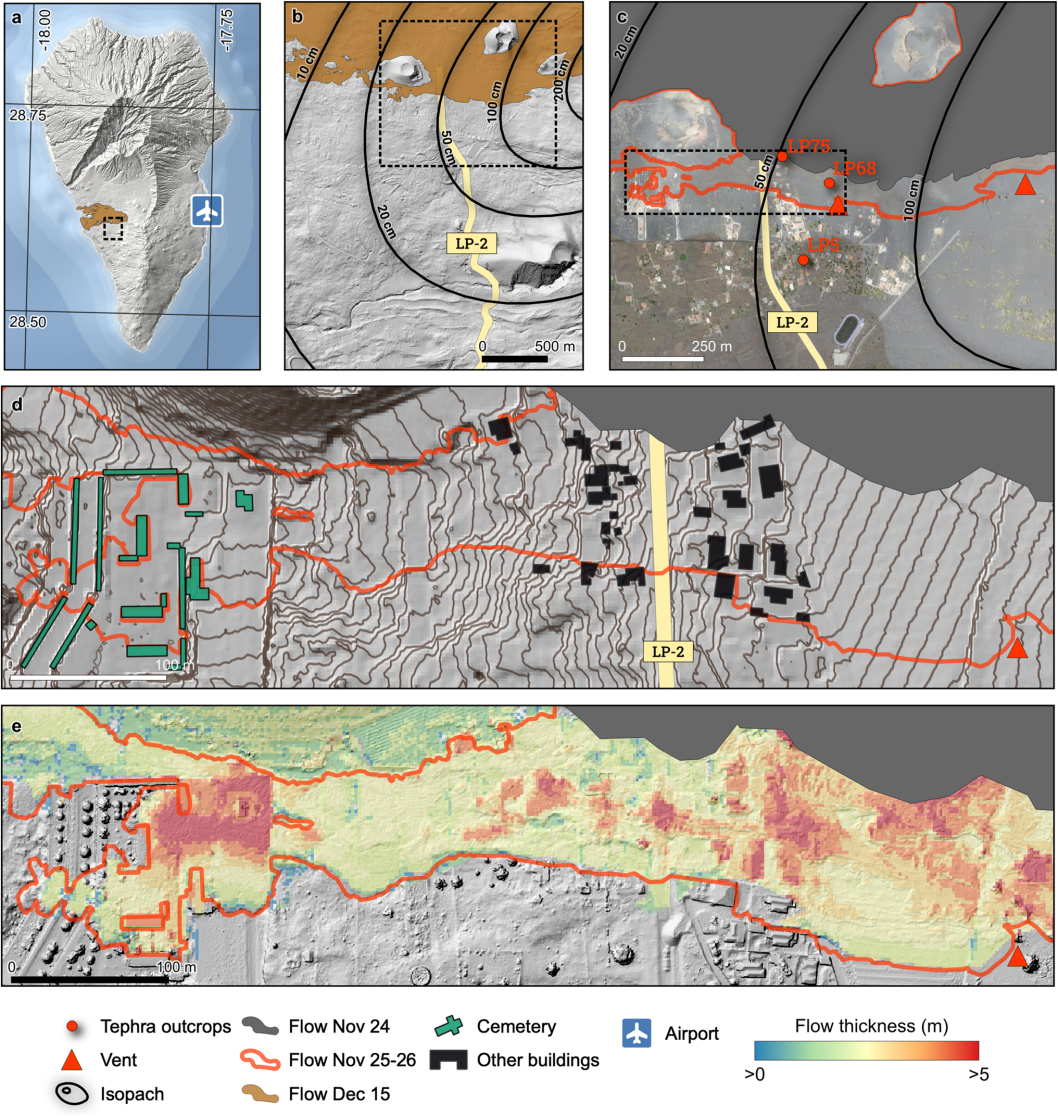
Overview of the study area. **a** Location of the 2021 lava flow; the dotted box shows the extent of **b**. **b** The locality of Las Manchas, with road network (OpenStreetMap contributors 2017), isopach of the tephra fallout blanket (Bonadonna et al. 2022) and lava flow extent from Copernicus Emergency Management Service (EMSR546 © 2021 European Union). Background topography is from Copernicus DEM (2023). **c** Zoom on the neighbourhood of Corazoncillo, showing the location of the tephra sections (LP5, 68 and 75; Bonadonna et al. 2022), the lava flow extent by Nov. 24 (grey) and Dec. 15 (red) and the sources of the second part of the Nov. 25–26 flow. Red triangles show eruptive sources. Background is Google Map (Map data ©2023 Google). **d** Zoom on the study area with hillshade and 1-m contour lines from the 2.5-m resolution digital surface model of Spain (Centro Nacional de Información Geográfica 2020) and building footprints in black from Dirección General del Catastro (2023). **e** Flow thickness overlaid on the post-eruption digital surface model of Civico et al. (2022). On all maps, a dotted rectangle indicates the zoom extent of the following subpanel

At a smaller spatial scale, the impact of lava flows is often considered to be binary (Deligne et al. 2022; Jenkins et al. 2017). Although this is certainly true within the consistently > 5-m-thick main lava flow field emplaced during the Tajogaite eruption (Civico et al. 2022), a subtle variability of impact mechanisms was observed on the edge of the flow (Meredith et al. 2022). On Nov. 25, 2021, a mostly undocumented short-lived flow inundated ~ 30 buildings in the neighbourhood of Corazoncillo on the edge of the main lava flow field. The neighbourhood was repeatedly exposed to deposition and subsequent clean-up of tephra fallout (Fig. 1). Three visits to the area, both during (but before lava inundation) and soon after the eruption revealed a complex impact sequence involving the interplay between mostly sustained and unsteady tephra deposition, clean-up operations and an intricate dynamics of flow emplacement. In this paper, we present the result of this field-based impact assessment to constrain impact mechanisms associated with each separate hazard and reconstruct a compound impact sequence that accounts for the interactions between hazardous processes and risk mitigation actions. This effort provides a detailed investigation of the compound impacts on the built environment caused by months of tephra fallout modulated by both clean-up operations and rainfall, and the emplacement of three stages of short-lived lava flows with varying morphologies and dominant impact mechanisms. Results outline some critical aspects required for the long-term risk mitigation and to support the crisis management of future similar eruptions in the Canary Islands and elsewhere.

## Case study

### The 2021 Tajogaite eruption

The island of La Palma, along with El Hierro, is the youngest and westernmost island of the Canary archipelago. The sub-aerial part of La Palma (Fig. 1) consists of four volcanic edifices, the youngest of which is the Cumbre Vieja ridge, active for the past 150,000 years (Troll and Carracedo 2016). The ridge has been host to half of the 14 historical eruptions that have occurred in the Canary Islands. After a week of seismic unrest and ground deformation, the Tajogaite eruption started on Sep. 19, 2021, opening an 86 day-long eruptive sequence that ended on Dec. 13. The initial phase of the eruption developed a 0.5-km-long, tectonically controlled, NW-oriented fissure that gradually built the ~ 4 × 10^7^ m^3^ Tajogaite pyroclastic cone where 11 vents developed (Romero et al. 2022; Civico et al. 2022; Pankhurst et al. 2022). The eruption consisted of the simultaneous emission of lava flows and a range of low to moderate explosive styles ranging from Hawaiian (i.e. sustained fountaining) to violent Strombolian activity (Bonadonna et al. 2023; Taddeucci et al. 2023). The final ~ 1.8 × 10^8^ m^3^ lava flow field was emplaced over an area of 12 km^2^, and the volume of the tephra blanket was estimated to be ~ 2.3 × 10^7^ m^3^ (Bonadonna et al. 2022; Civico et al. 2022; Fig. 1a, b).

### The November 25–26 flow

On Nov. 25, a lava flow inundated a narrow corridor of land just south of the lava flow field emplaced during earlier stages of the eruption and impacted the neighbourhood of Corazoncillo in Las Manchas (Fig. 1c). The flow extended downslope for 1.5 km in a westerly direction, inundating along the way a solar panel complex, the easternmost houses in Corazoncillo and the Cementerio de Nuestra Señora de los Ángeles in Las Manchas (hereafter referred to as cemetery). The flow stopped against the main, thick ʻaʻā flow. Based on the reference stratigraphy of Bonadonna et al. (2022), the flow occurred during the transition between the Middle Unit (MU) and the final Upper Unit (UU) of the tephra blanket. This latest 1.5-km-long flow has a thickness of < 5 m, locally < 2 m (Civico et al. 2022), and impacted 30 buildings (Fig. 1d, e).

## Methods

This study synthetises fieldwork undertaken during three deployments in Oct. 21–Nov. 5, 2021 (during the eruption), Feb. 7–18, 2022, and May 14–20, 2022. All campaigns aimed at characterising the tephra blanket, summarised in Bonadonna et al. (2022, 2023) and conducting post-event impact assessments (post-EIA) of the built environment (Reyes-Hardy et al. under review).

### Hazard properties

The main objective of the study is to assess the nature and extent of impacts caused by the simultaneous emission of tephra fallout and lava flows during long-lasting hybrid eruptions. For tephra fallout, we consider the load as the critical hazard metrics, the spatiotemporal evolution of which was reconstructed using the isopach maps of Bonadonna et al. (2022). For lava flows, existing observations on past eruptions suggest that impact can occur from a variety of hazard properties including static pressure, dynamic pressure and/or temperature, each resulting in specific impact mechanisms (e.g. direct lava flow impact vs. indirect impact from fire ignition; Meredith et al. 2022). Approximating the hazard impact metrics of lava flows is, therefore, difficult since each hazard metrics depends on the spatiotemporal evolution of critical properties (e.g. thickness, velocity, temperature, viscosity) that cannot be directly measured from emplaced lava flows. As an alternative, we reconstruct a qualitative emplacement sequence based on the mapping of surface textures and their relationships, physical features and evidence of interaction with both natural and urban microtopography, providing first-order constraints on the dynamics of flow emplacement.

### Post-event impact assessment

**Fig. 2:**
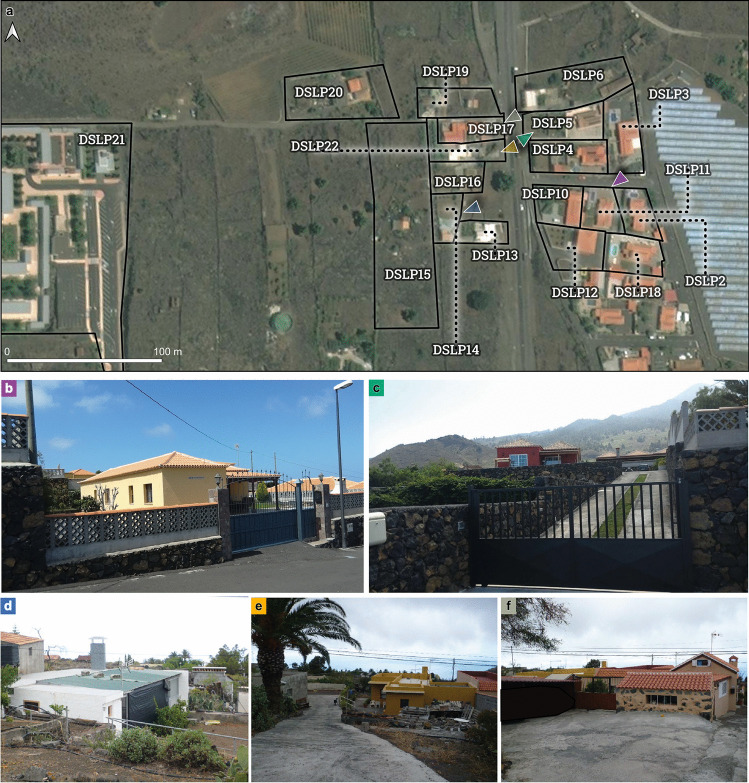
Buildings impacted by the Nov. 25–26 flow. **a** Reference ID of all buildings. Black contours show the extent of their respective parcel from the official cadastre (Dirección General del Catastro 2023). Coloured triangles show the approximate location and orientation of the pre-eruption pictures (**b**–**f**; Dirección General del Catastro 2023). **b** Example of a reinforced concrete building with complex roofs (DSLP11; here showing only the 4-pitched roof at the back of the building with a 1-pitched roof on the front porch). Notice the drop in elevation (left) and the walls in mixed masonry between properties. **c** DSLP5 (left) and DSLP 3 (centre) taken from the LP-2 road, illustrating terraces. These houses have similar typologies to **b**. **d** DSLP14, showing part of an aggregate of structural units in regular masonry with a flat, metal sheet roof. The rest of the building is visible to the left, and a secondary structure to the right. **e** DSLP16 (left) and DSLP22 (right), which is a structural aggregate with DSLP17. DSLP22 has a complex geometry and a flat concrete roof. **f** DSLP17, illustrating a structural aggregate of units made of different material (i.e. reinforced concrete, regular and irregular masonry) and a complex geometry. DSLP17 contains 1-, 2- and 4-pitched tile roofs with a flat, concrete roof at the back

The first and second post-EIA investigated the typology and impacts of 764 buildings affected by the eruption. During the first visit, when buildings in Corazoncillo were only affected by tephra, access to buildings was prohibited, and description of the building typology was mostly done from observations of the structure’s exterior. At the time of the second visit, after eruption cessation, Corazoncillo had been inundated by the flow. East of road LP-2, where flow thickness generally exceeds 3–4 m (Fig. 1e), most of the buildings were surrounded by lava up to the roof, with more exposition of the down-flow façade in the wake of the flow. During the third visit, we focused on 30 buildings affected by the flow, detailing impacts related to tephra load and lava flow inundation and describing the relevant hazard intensity metrics. The 30 buildings are spread on 18 cadastre parcels (Fig. 2), all classified as *residential* except for the cemetery (DSLP21, classified as *public*) and one farming building complex (DSPL5, classified as *industrial*).

Each building was catalogued in terms of building properties, including material, geometry, number of storeys, number and type of openings, roof type and typology and presence of secondary buildings (e.g. small independent constructions with a minor function). Only those buildings that were partially or totally collapsed provided an insight into the internal structural construction of the building. For buildings where direct pre-lava flow observations were not possible for reasons stated above, the analysis was complemented by extrapolation of direct observations in the area (e.g. building material) and pictures obtained from the official cadastre repository (https://www.catastro.minhap.es/webinspire/index_eng.html) and Google Street View (e.g. location of openings, typology). Each parameter was detailed in the perspective of the flow direction, with the aim of identifying the nature and extents of impact mechanisms caused by tephra and lava. We recorded evidence of mechanical (e.g. cracks, partial to total roof collapses) and thermal impacts (e.g. partially to totally melted synthetic seals and softened metal seals around windows and door openings) and attempted to reconstruct the sequence of hazards reproducing the observed impacts (e.g. different phases and pathways of flow inundation). A compilation of field observations is available in the Online Resource 1.

### GIS setup and material

The GIS analysis was setup in *QGIS v3.26* (QGIS Development Team 2022) using a UTM 28N projection on a WGS84 datum (EPSG:32628). The building survey was processed and analysed in *Python v3.9* using the *GeoPandas v0.11* library. The pre- and post-eruption topography was obtained from the 2.5-m resolution digital elevation model (DEM) of the Centro Nacional de Información Geográfica (Centro Nacional de Información Geográfica 2020) and from Civico et al. (2022), respectively. Building footprints and their associated parcels come from Dirección General del Catastro (2023). Photo mosaics from unmanned aerial vehicles (UAV) were retrieved from the *La Palma Open Data* repository (Cabildo Insular de La Palma 2023) and manually georeferenced.

## Results

### Description of the built environment

Figure 2 illustrates the distribution of the sampled buildings and their typologies. East of road LP-2, the average slope is 4–6° and was mostly terraced to accommodate buildings (Fig. 1d). The wide terrace with a constant northwest-oriented slope < 4° east of the DEM is the location of a solar panel farm. Residential buildings were dominantly built after 2000 with reinforced concrete frame and limestone walls except for DSLP6. All but DSLP18 are single-storey buildings. Although main buildings are single structural units, most have complex geometries with multiple low-angled 4-pitched complex tiled roofs. Roofs are made of several brick infill walls (2–3 depending on the roof size) lying on concrete beams, on top of which corrugated fibre-cement panels are fixed and covered with tiles. The main entrance to most buildings faces upslope and towards the east (i.e. towards the source of the flow), with the west-oriented facades usually containing wide openings. Most buildings have one or two porches, which are timber structures with 1-pitched tiled roofs connected to the main building by wooden beams, and secondary structures (e.g. outdoor kitchen, garage). Various groups of houses (i.e. DSLP3–5, DSLP 2, 10–11 and DSLP12, 18) are delimited by east–west oriented ~ 1.5-m tall masonry walls. Within these groups, each house is delimited by similar north–south–oriented walls that correspond to changes in elevations of ~ 1–2 m between each parcel. The closest terraces to road LP-2 overhang > 2 m above the road.

West of road LP-2, the slope increases to 6–9°, and the closest buildings are located at a lower elevation than the road (Fig. 2e). Except for the DSLP20 (i.e. the cemetery), buildings are all residential and more heterogeneous in their construction type and age (i.e. 1964–2008). Buildings are often aggregates of various structural units successively constructed over time (Fig. 2f). Construction types are dominantly regular and irregular (i.e. block fragments) masonry, sometime co-occurring in a single building. Roofs are either 1, 2 or 4-pitched tile roofs or flat concrete, but secondary structures also have metal sheet roofs. In areas of steeper slopes, houses accommodate the slope with various single-storey elements built at different levels (e.g. DSLP13, 17). Parcels are not as delimited with robust walls as the region east of road LP-2, and few walls lie in a north–south direction. The 200 m between the last residential buildings and the cemetery is terraced land containing a few agricultural parcels.

### *Hazard sequence*

#### Tephra fallout

**Fig. 3:**
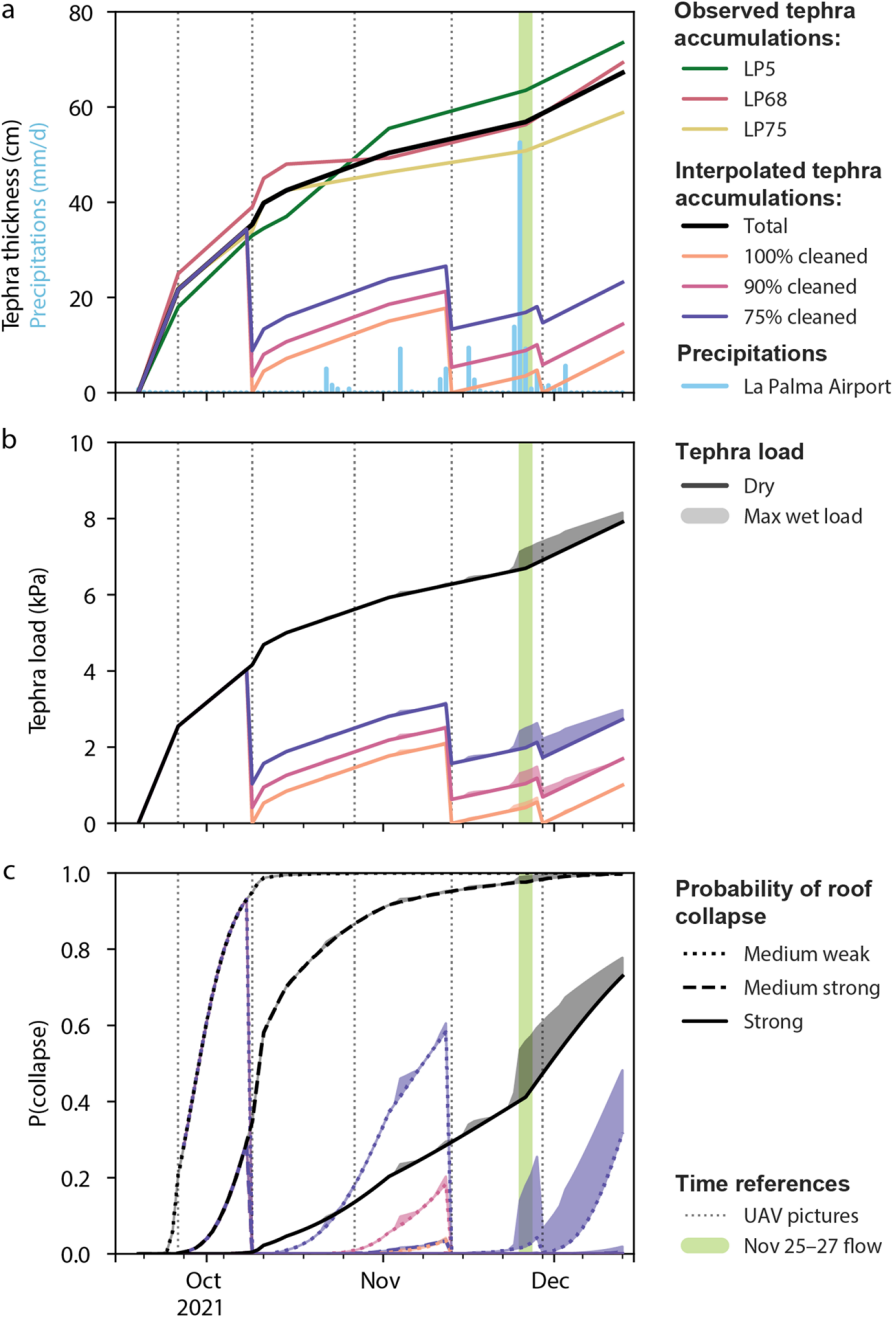
Evolution of the tephra fallout blanket throughout the eruption in terms of **a** thickness, **b** load and **c** probability of roof collapse. The green shaded area indicates the Nov. 25–26 flow, and vertical dotted grey lines show to the dates of the pictures in Fig. 4. **a** Observed tephra thicknesses at different outcrops (LP5, 68 and 75; Fig. 1c) and the interpolation at Corazoncillo (thick black line), with associated thicknesses considering various clean-up efficiencies. Blue vertical lines are daily precipitations at the airport (AEMET 2023). **b** Equivalent tephra loads. For each of the four clean-up scenarios, the maximum load increase due to rain is shown as a shaded area. **c** Probability of roof collapse considering medium weak (MW; dotted line), medium strong (MS, solid line) and strong (ST, dashed line) roof classes of Spence et al. (2005). For each subplot, legend items that are not specified follow entries previously defined

**Fig. 4:**
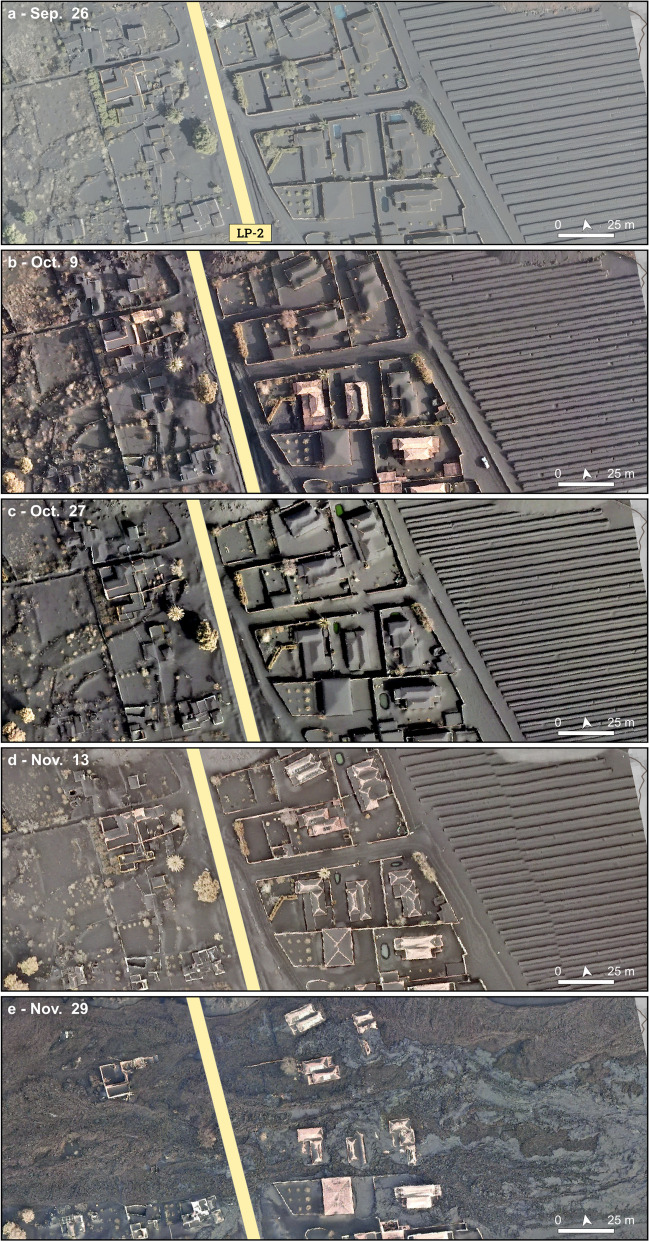
Evolution of the tephra fall and lava flow at Corazoncillo using UAV pictures from Cabildo Insular de La Palma (2023). Note the slight picture rotation from the geographic north

The neighbourhood of Corazoncillo was impacted by tephra fallout throughout the eruption. Based on the reference stratigraphy (Bonadonna et al. 2022), the tephra blanket was divided in three units: the Lower Unit (LU), deposited between Sep. 19 and Oct. 10–12; the Middle Unit (MU), deposited between Oct. 10–12 and Nov. 25–27 and the Upper Unit (UU), deposited until Dec. 13. The temporal evolution of the tephra blanket on Corazoncillo was estimated using the three closest stratigraphic sections (Fig. 1c) and indicated a total tephra section of 67 cm accumulated over 86 days, with the highest accumulation rates occurring during LU (Fig. 3). Accumulation rates on buildings were modulated by regular clean-up operations. Although officially organised by the *Unidad Militar de Emergencias* (UME) from Oct. 15, the visual analysis by unmanned aircraft vehicle (UAV) surveys reveals that clean-up operations occurred as early as Oct. 9 (Fig. 4). Visual evidences from UAV images suggest that clean-up operations lasted for a few days and that not all houses were systematically and entirely cleaned, thus preventing an accurate constraint of the timing of tephra removal. Here, we consider that clean-up operations started on Oct. 9 with a second one occurring on Nov. 13. To account for uncertainties on clean-up efficiency, we consider three scenarios of 100%, 90% and 75% of tephra removal from the cumulative accumulation for these dates. Note that the choice of these scenarios is arbitrary and, rather than reflecting the operational reality of clean-up operations, serves as an illustration of the variability inherent in our analysis.

Tephra thicknesses were converted to a load using a deposit density of 1386 kg m^−3^ to estimate the potential impact of the tephra blanket on the roofs in Corazoncillo, which represents a mean value for the stratigraphic LU and MU units (Bonadonna et al. 2023). In the absence of a dedicated fragility model for roofs in La Palma, we estimated the roofing stock of Corazoncillo to be comprised in the medium weak (MW), medium strong (MS) and strong (ST) roof classes of Spence et al. (2005) (Table 1) and used these fragility curves to illustrate the temporal evolution of collapse probability during the eruption. Without clean-up operations, a 50% probability of roof collapse could have been exceeded by Sep. 26, Oct. 10 and Nov. 6 for MW, MS and ST classes, respectively (Fig. 3c). Instead, clean-up operations resulted in a virtually null collapse probability for ST roofs and < 20% for MS roofs. For MW roofs, only the 75% clean-up efficiency scenario results in collapse probabilities > 50%, whereas a 90% clean-up efficiency prevents collapse probabilities exceeding ~ 40%.

**Table 1:** Description of European roof typologies of Spence et al. (2005). Fragility curves for roof collapse are estimated as a cumulative density function of a Normal distribution using the natural logarithm of both the tephra load and the mean collapse load with a standard deviation of 0.2 (Spence et al. 2005; Jenkins et al. 2014; Biass et al. 2016)

Roof class	Description	Mean collapse load (kPa)
MW (medium weak)	Sheet roof on timber, average quality, average or good quality tiled roof on timber rafters or trusses. Steel or precast reinforced concrete joists and flat terrace roof	3.0
MS (medium strong)	Flat reinforced concrete roof not all above characteristics, sloping reinforced concrete roof. Sheet roof on timber rafters or trusses, good quality and condition, designed for cyclone areas	4.5
ST (strong)	Flat reinforced concrete roof designed for access; recent, good quality construction, younger than 20 years	7.0

#### Lava flow inundation

**Fig. 5:**
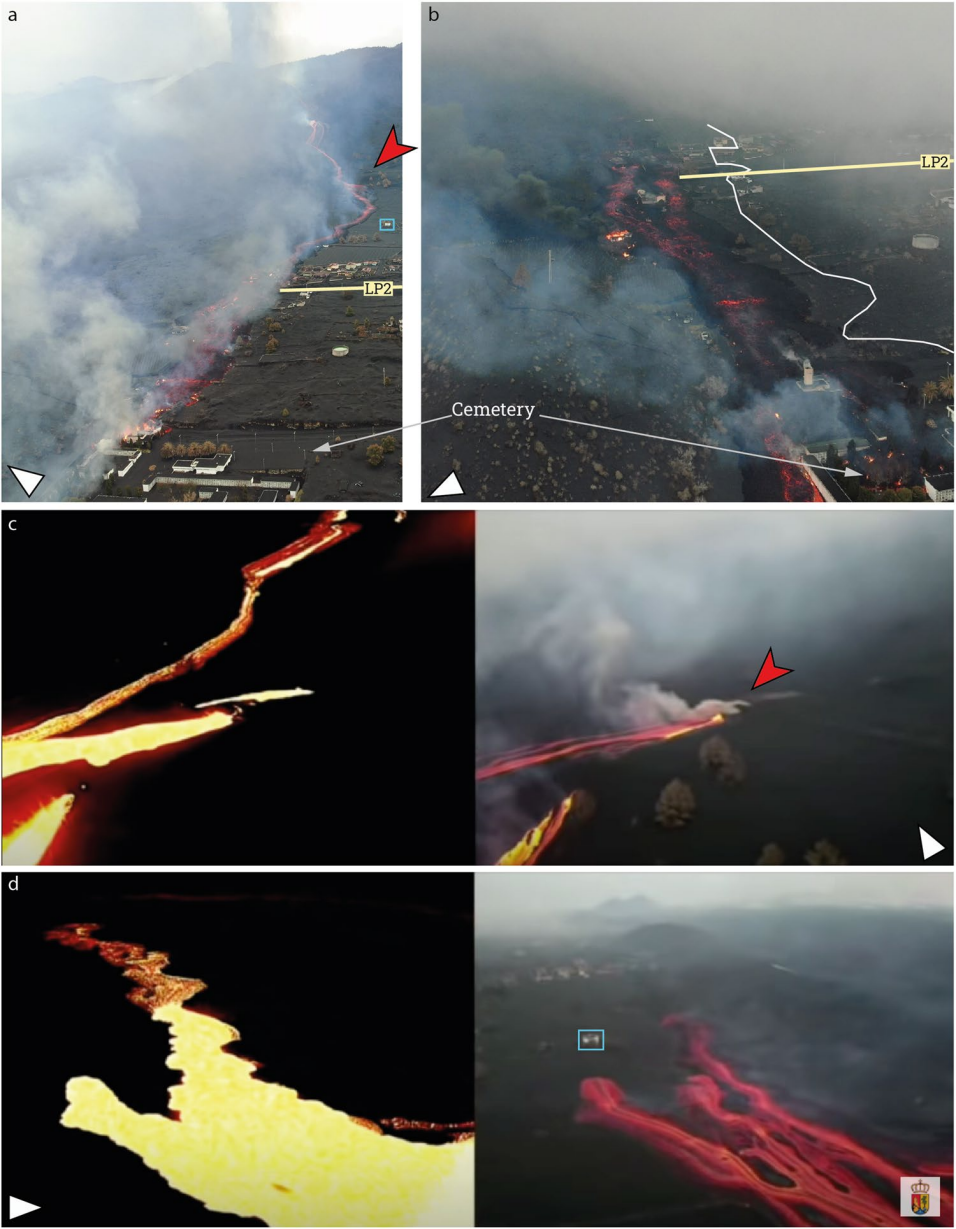
Evolution of the flow emplacement inferred from **a**, **b**: Cabildo Insular de La Palma (2023) and **c**, **d** Ayuntamiento de El Paso (2021). **a** UAV image captured on Nov. 25 at 12.15 local time. The approximate location of road LP2 is indicated. The red arrow indicates the easternmost vent in Fig. 1c and is discussed in **c**. The cyan box indicates the location of a house and is used as a placeholder visible on **d**. **b** UAV flight on Nov. 25 at 13.10. The white line estimates the southernmost edge of the final lava flow that was emplaced on Nov. 25. **c** Side-by-side thermal and optical images centred on the fissures that opened under a house (red arrow) captured in the evening of Nov. 25. The thermal image shows the presence of the earlier Nov. 25 flow as colder (i.e. less bright on the thermal imagery) and the two fissures of the new source. The cyan box corresponds to the house in **a**. **d** Same as **c** but showing the second flow overlaying the first one. White arrows indicate the approximate north

Due to scarce information on the timing and dynamics of the flows that are available in official reports, we reconstruct the flows inundation sequence from a combination of observations made by reviewing online footage and characteristics of the flow as seen in the field. All times are reported as local (i.e. UTC + 1). On Nov. 25, a new lava flow originating from the south part of the Tajogaite cone (hereafter referred to as *the first flow*) inundated a narrow corridor flanking the south edge of the ʻaʻā flow emplaced at the end of Oct. 2021 (Fig. 1c). The earliest evidence of this flow comes from pictures captured during a UAV flight from the local El Paso Government (Ayuntamiento de El Paso 2021) on Nov. 25 at 12.15 and shows a lava channel that transitions into an ʻaʻā flow at the contact of the easternmost houses of Corazoncillo (i.e. DSLP3, 4 and 5; Fig. 5a). The flow split around DSLP3, forming a northern branch topographically constrained in the north by the earlier Oct. flow and a southern branch channelising in a street. Both branches reconnected west of DSLP10. The south branch is an ʻaʻā flow that extended down to a crematorium tower at the cemetery.

A second UAV flight at 13.10 provides a close temporal comparison of the evolution of the flow (Fig. 5b). The channel transitioned into a matured, thick ʻaʻā texture statically ponding against DSLP 3, 4 and 5. Under the influence of microtopography (e.g. houses, walls and elevation drops from terraced urban and agricultural parcels), the flow downstream of DSLP5 appears relatively depleted in crust compared to the morphology upstream of DSLP3, better exposing the glowing core. The northern branch of the flow developed into an ʻaʻā flow around DSLP20, setting it on fire. To the W, the S part of the flow entered the perimeter of the cemetery and inundated the northernmost buildings, while the N part channelised against a wall in the cemetery, creating a topographically constrained narrow ʻaʻā flow that advanced by 250 m since 12.15. At 16.30, an INVOLCAN footage shows that the tip of the flow as a thin, stalling ʻaʻā lobe. Although the location cannot be precisely constrained, it is not yet in contact with the Oct. part of the main ʻaʻā flow, suggesting that the flow front continued to advance after 16.30.

Video footage from the INVOLCAN twitter account (INVOLCAN 2021a) first reported the opening of a new fissure under a house at 17.30, showing new lava branching out from a E-W–oriented fissure. This flow is hereafter referred to as *the second flow*. Further INVOLCAN videos between 18.00 and 19.00 (INVOLCAN 2021b, c) captured the junction of the two branches, showing an immature ʻaʻā’ texture with a poorly developed brittle crust with relatively small slabs over a fluid, glowing molten core, with an estimated advance rate of 600 m h^−1^ and a temperature of 1024 °C. Two UAV flights were published by Ayuntamiento de El Paso (2021) and dated Nov. 16 at 8.30. Footage from the first flight consisted of side-by-side optical and thermal imagery revealing how the second flow inundates the surface of the still-moving first flow (Fig. 5c, d). Due to the similarity in flow extent and morphologies with the INVOLCAN videos, we infer that the timestamp is incorrect and that this footage was captured in the evening of Nov. 25. Footage from the second flight showed an emplaced flow with no sign of activity at the fissure, suggesting that activity at the fissure stopped and that the flow was fully emplaced by the morning of Nov. 26.

The dynamics of emplacement of the flow during the night is therefore inferred from field observations. The flow generally widened towards the south (Fig. 5b). A main branch of the second flow inundated DSLP2, 10 and 11, topographically constrained in the south by the walls of DSPL12 and 8 and in the north by the initial ʻaʻā flow. The flow then moved towards the west with lateral breakouts affecting DSLP 13, 14 and 15 before reaching the cemetery and further inundating its south buildings with a dominantly ʻaʻā texture. However, UAV pictures show the occurrence of a widespread pāhoehoe lava sourced with a sharp origin east of the solar panel farm (see Figs. 1e and 4). Field observations reveal a source located on the edge of the second flow and evidence of oxidised spatters and back drainage features. The pāhoehoe surface shows transitions from smooth, ropy, slabby to rubbly textures and inundated the easternmost houses (i.e. DSLP 2, 3, 4, 5, 10, 11 and 18). Textural relationships show that the thin pāhoehoe flow emplaced on a still moving ʻaʻā substrate. The origin of this flow is further discussed in the “Where did the pāhoehoe come from?” section.

The final stage of the flow consisted of drainage episodes. Firstly, two main drainage channels are observed, one corresponding to the south branch of the first flow and the other to the second flow, both constrained by tall walls longitudinally oriented with respect to flow direction. Since pāhoehoe is pristine in the North Channel but shows a transition from smooth to rubbly and ʻaʻā textures in the South one, we infer that the channel associated with the early flow drained before the emplacement of the pāhoehoe sheet. Secondly, deflation cracks are observed around most houses. This suggest that the microtopography caused the flow to statically pond against the structures, allowing a maximum inundation height to be maintained for a sufficient time to develop a crust before draining after lava effusion ceased at the source.

### Impact mapping

#### Impact at the end of October

**Fig. 6:**
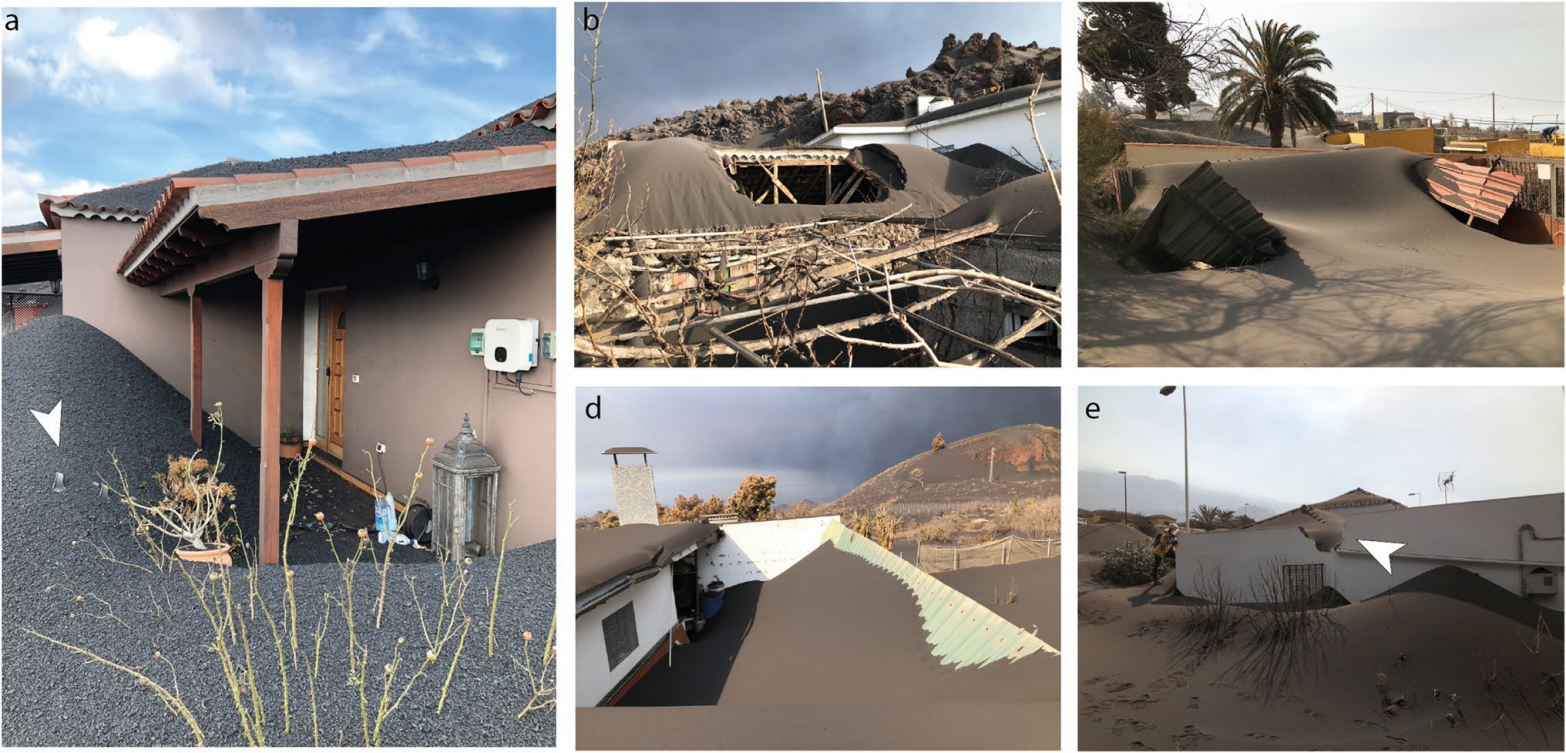
Various degrees of impacts from tephra fallout observed during the Oct. 21–Nov. 5, 2021, visit. **a** Porch and tephra burial (DSLP4), with roof fragments visible in the tephra deposit (white arrow); **b** secondary farming unit (DSLP19); **c** garage roof (DSLP17); **d** front patio (DSLP14); **e** partial wall collapse (white arrow) due to a lateral load caused by tephra accumulation (DSLP13)

The first field deployment (Oct. 21–Nov. 5) provides a syn-event impact assessment for tephra fallout, at which time the deposit is estimated to have been ~ 50–55 cm thick in Corazoncillo (Fig. 3a). At least one clean-up operation had already taken place by then (Fig. 4), and the spatiotemporal reconstruction of the tephra fallout blanket suggests a deposit thickness ranging between ~ 10 and 25 cm. This is mostly consistent with observations in the field, though some buildings west of road LP-2 showed larger accumulations. Except for the only building of DSLP6 unaffected by the Oct. lava flow (a long agricultural building in irregular masonry with a poorly maintained tile-covered timber roof that suffered total roof collapse), roof collapse from tephra fallout occurred only on annexes to the main structure (e.g. patios with sheet metal roofs) and secondary structures (agricultural units with tile roof and timber structure, garage roofs with sheet metal roof and structure; Fig. 6). Following existing damage states (DS; Table 1) for tephra fallout (Jenkins et al. 2015), these impacts vary between DS 3–5, ranging from partial roof collapse (Fig. 6b, d) to total structure collapse (Fig. 6c). For the case of DSLP14 (Fig. 6d), the collapse appears to be mostly due to failure of the connection between roof and main building. DSLP13 illustrates a complex building geometry in which the roof is surrounded by a flat balcony with a wall standing higher than the roof’s overhang (Fig. 6e). In this case, the balcony served as an accumulation area for tephra sliding off the roof, creating a sufficient lateral load to produce a partial wall collapse. Figure 6a shows typical wooden porch structures attached to houses east of road LP-2 and illustrates the overthickening of the tephra deposit around the building due to clean-up operations. Fragments of roof cement observed in the deposit suggest breakage occurring during clean-up.

#### Impact after the November 25–26 flow

##### East of road LP-2

**Fig. 7:**
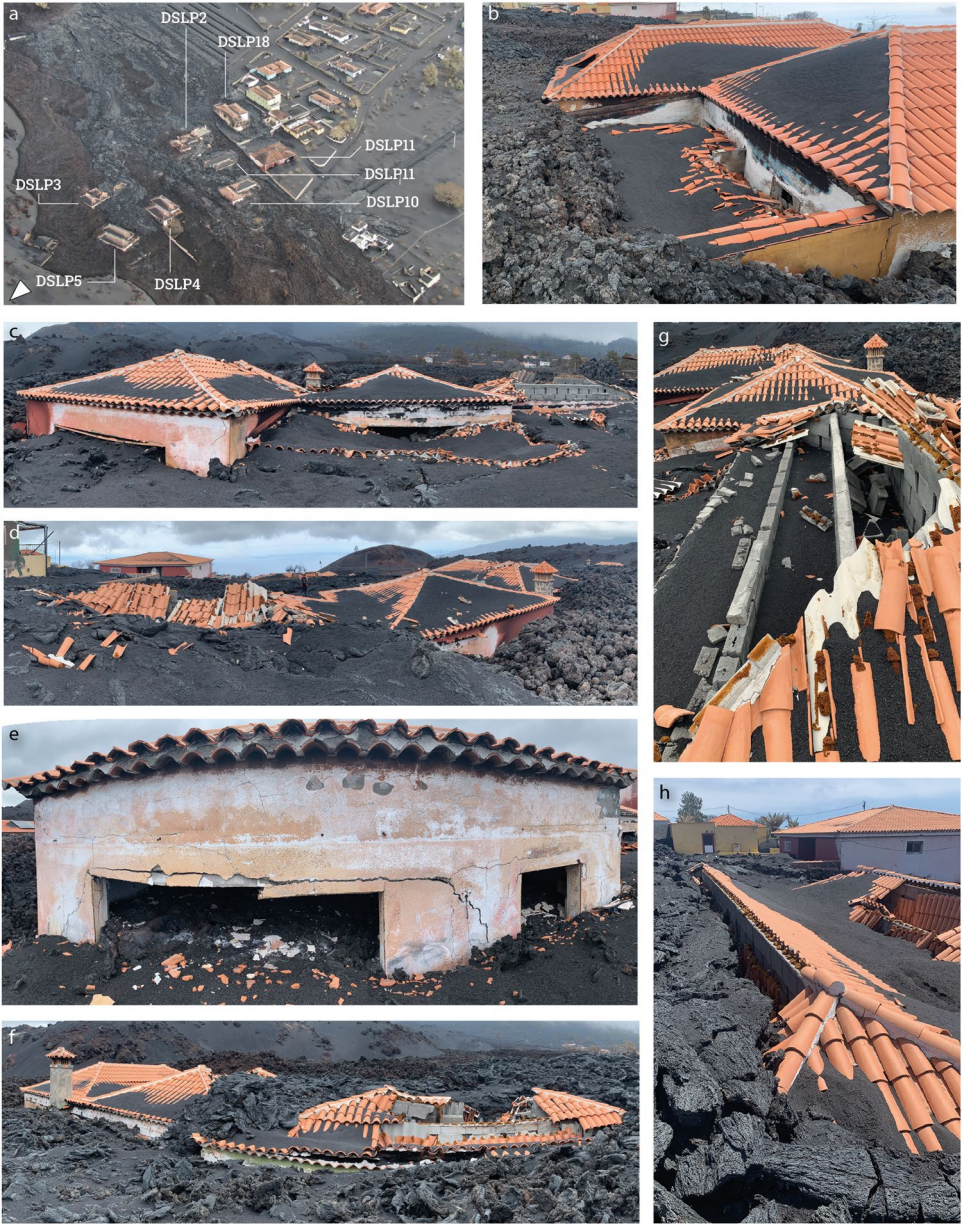
Illustration of impacts east of road LP-2 after the 25–26 November lava flow. **a** UAV picture taken on Nov. 27. The white arrow indicates the approximate north direction. **b** DSLP4, showing the collapse of a 1-pitched auxiliary roof due to burnt timber supporting rafters (front) and partial roof collapse due to tephra load (back). **c** Down-flow-facing side of DSLP2 illustrating structural diagonal impacts to the concrete columns at the edge and associated rotation of the central part above the door (left), collapse of a porch (centre) and total roof collapse (right). Notice partial roof collapse at the junction between the left and the central elements. **d** Up-flow-facing side of DSLP2 showing pāhoehoe inundation on roof (left) and static ponding of the earlier ʻaʻā flow against the wall (right). Notice the decolourisation halo due to pāhoehoe. **e** Illustration of diagonal, horizontal and stair-shaped cracks on the down-flow-facing façade of DSLP10. Note that the apparent rounding of the building is an artefact caused by the panoramic picture. **f** Down-flow view of DSLP3 illustrating total roof collapse (right) and pāhoehoe inundation between roof structures. Note that a porch was present along the entire side of this building but has been covered by pāhoehoe. **g** Structure of the longest roof of DSLP2 revealing the presence of pitched roof with infill in concrete blocks covered with fibre-cement cladding with tiles. **h** DSLP11 showing the collapse of one pitch of the roof from pāhoehoe inundation without impact to the main longitudinal wall (left) and partial roof collapse (right)

Houses east of LP-2 provide a uniform population of building construction. Adopting recent damage state scales for lava flow (Meredith et al. 2022; Table 1), these impacts are classified either as DS 1 (DSLP12; minor surface damage from lava flow on one wall without inundation), DS 4 (DSLP18; > 60% flow inundation) or DS 5 (DSLP3, 4, 5, 11 and 13; total burial). Amongst houses classified as DS 5, all are single-storey and no primary structural units collapsed despite inundation up to roof level on their up-flow sides (i.e. final flow thickness > 3.5 m; Figs. 1e and 7a) and evidence of lava flowing through, statically ponding in, and escaping through the openings of the down-flow side. On the contrary, no evidence of any secondary structure associated with these buildings remains. No up-flow side of primary buildings could be observed, but down-flow and lateral facades show a variety of cracks including diagonal and x-shaped, horizontal, vertical and stair-shaped fissures (Fig. 7). Amongst this building typology, only diagonal and x-shaped cracks are interpreted to reveal a structural impact when occurring on the edges of the building and imply damage to the concrete structure (Fig. 7c–e). Similar patterns occurring within walls (and often associated with stair-shaped cracks) denote a non-structural impact to infills. Both are attributed to lateral pressure from static lava ponding within buildings. Horizontal and vertical cracks are interpreted to reflect a static load, either caused by tephra or lava. Clamping vertical cracks were observed in one instance (DSLP4) at the junction of two parts of a structural unit. Figure 7b shows the main (right) and an auxiliary part with a different roof typology (i.e. 1-pitched tile roof on a timber structure). We attribute these clamping cracks to the effect of load and a poor design of the junction between the two parts of the building. For this building typology, horizontal cracks, mainly located in the line of connection between vertical and horizontal elements, are observed in conjunction with evidence of structural impact (Fig. 7e) and are attributed to tephra load after weakening the concrete structure.

All buildings show evidence of thermal impacts (e.g. decolourisation of outside walls, burnt elements and ductile deformation). Regarding façade colours, comparing our pictures with the official cadastre reveals that most buildings have been repainted with different shades through time. Although this prevents a quantitative interpretation (e.g. constraints on temperature), some general observations can be made. Firstly, halos of decolourisation around ʻaʻā morphology are more widespread on the lateral rather than up-flow sides of the buildings. Secondly, halos are generally more widespread surrounding pāhoehoe lobes (Fig. 7d). Thirdly, decolourisation occurs around charred beams and is most frequent around windows (Fig. 7b, d). Additional evidence of thermal effects includes melted plastic, rounded window glass fragments (sometime found rafted from origin and molten on pāhoehoe surface), ductile deformation of metal beams and corrosion of metallic elements (e.g. gutters; Osman et al. 2017). All these observations suggest that thermal impacts were caused by three different mechanisms. These include (i) thermal conduction from the initial contact with an immature ʻaʻā flows morphology, which burnt basal wood elements triggering (ii) fires causing zones of decolourisation around windows on outside facades similar to observations made during wildfires (Papalou and Baros 2019) and (iii) thermal radiation from more mature lava morphologies. Regarding this last aspect, observations illustrate how the radiative power of lava flows is modulated by crust thickness, which is greatest for pāhoehoe, and gradually reduced when ʻaʻā is flowing along the side of or is ponding against buildings.

Various degrees of roof collapse occurred due to different mechanisms. Figure 7c, g provides evidence of the roof structure of this building typology, showing five longitudinal brick walls serving as supports to the fibre-cement tile cladding. Structures DSPL2, 3 and 11 (Fig. 7f–h) illustrate how the inflation of the ʻaʻā flow up to the roof level provided an elevated substrate for late-stage pāhoehoe to inundate and collapse roof elements. At DSLP11, pāhoehoe lobes inundated the roof surface, causing breakage of the fibre-cement cladding under load. In this case, the down-flow roof pitch remained conspicuously intact. At DSLP2 and 3, the lateral stress from flow inundation additionally caused the collapse of the main roof wall, which caused the down-flow roof pitch to also collapse. Interestingly, a significant pāhoehoe inundation occurring at the junction of two roofing structures—and presumably aligned with concrete structural elements—induced structural damages but no structure collapse. Collapse of porches (e.g. Figure 7c) and auxiliary roofs (Fig. 7b), due to burning of the supporting timber structures, results in a coherent collapse of the roof on an already present and inflated ʻaʻā surface. Field relationships indicate that the collapse of these elements occurred before pāhoehoe inundation.

Partial roof collapses attributed to tephra load are also observed (Fig. 7b, h). In these cases, fibre-cement panels either broke or rotated without breaking due to the failure of their connection to the supporting walls. These dominantly occur in accumulation valley regions between two opposite pitches of a roof (Fig. 7h). At the time of the first visit, we observed an overthickening of the tephra deposit in these accumulation regions along with a relatively clean roof surface (i.e. with little to no remaining tephra) closer to the ridge (i.e. the top) of the roof. This thinning was attributed to the sliding of the tephra deposit on the roof pitch (Osman et al. 2022). Despite being regularly cleaned before Nov. 25, daily precipitation rates show that 50 mm of rain fell at the La Palma airport on Nov. 24. The increase of static load due to rainfall probably contributed to these impacts either soon before, during or soon after lava flow inundation (Macedonio and Costa 2012; Williams et al. 2021).

**Fig. 8:**
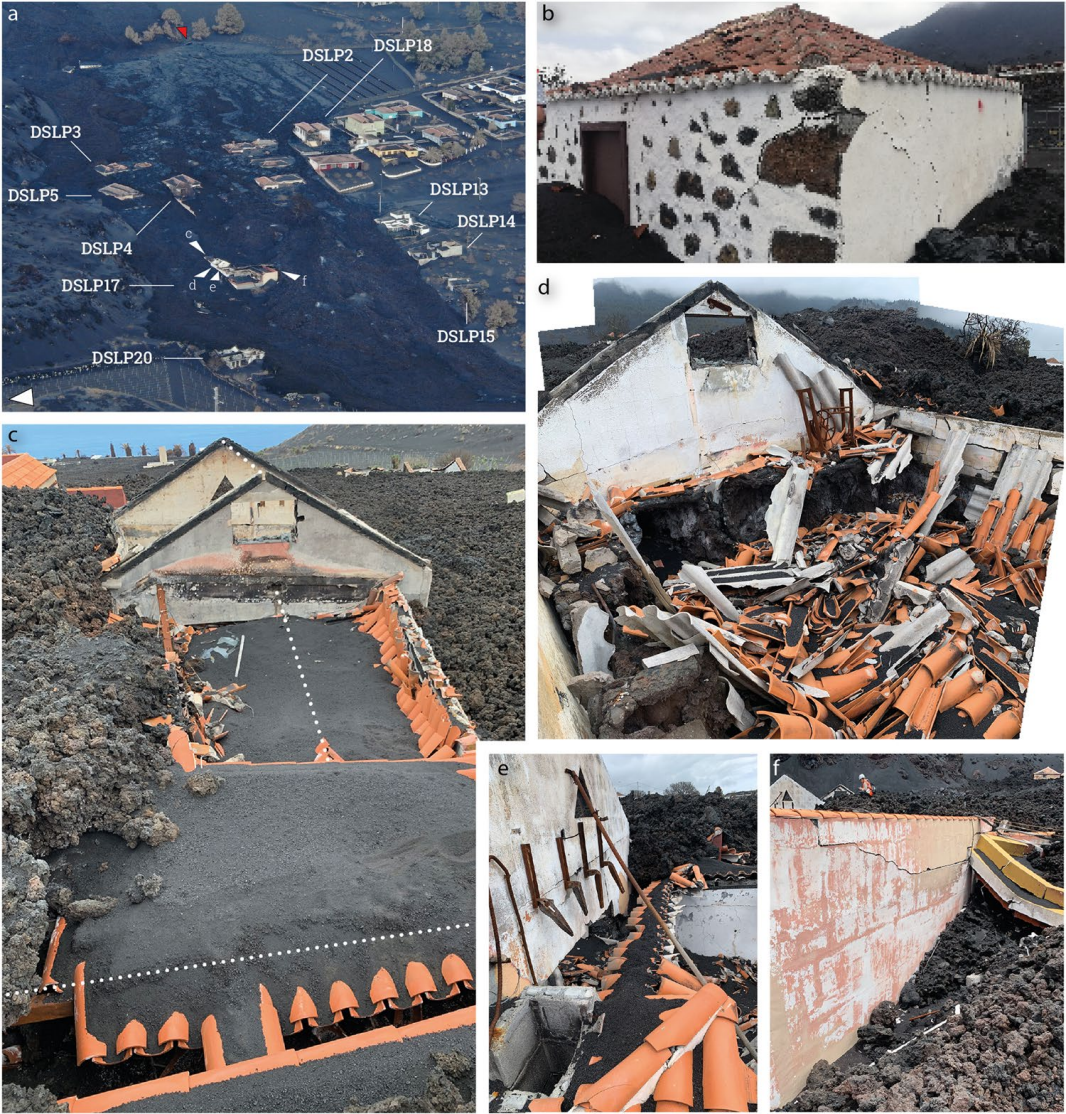
Illustration of impacts west of road LP-2. **a** UAV picture taken on Nov. 27 with labels for selected houses. Arrows around DSLP17 indicate the angle of pictures **c**–**f**. The red arrow shows the source of the pāhoehoe flow. The white arrow indicates the approximate north direction. **b** Cracks on the down-flow side of DSLP13 and **c**–**f** DSLP17. In **c**, white dotted lines indicate the location of the primary roof beam. Note that the horizontal crack on main roof wall in **d** is straight and only appears rounded due to panoramic photography

Both flow topography (Fig. 1e) and UAV pictures (Figs. 4e, 7a and 8f) show late stage drainage features, which translate into breakage of the surface lava crust and rafting of elements on their surface. For instance, the southernmost channel shown in Fig. 1e contains fragments of roofs from DSLP2 a few metres away from their source and brecciated within the ʻaʻā flow. This suggests that drainage events, which sometime occur after cessation of effusion at the source, might act as an additional down-flow process that can further impact damaged structures.

Finally, we did not find wall fragments, which, given the non‐flammable nature of the material, suggests that either (a) collapsed walls were brecciated in the 'a'ā flow and are not visible at the surface or (b) the walls were buried without collapsing. In regions of high walls and/or relatively thin flow, walls are still visible and standing (Figs. 5e and 7a). Although this is especially true for those aligned along the flow direction, some orthogonal walls were observed to have also withstood lateral pressure (e.g. DSLP5, 10; Fig. 7a). In many cases, flow textures tend to indicate that the emplacement was modulated by walls, either favouring static ponding or tearing of the crust due to elevation drops for walls perpendicular to flow direction or enabling channelisation of lava when walls topographically confine the flow parallel to the flow direction. These interpretations of flow textures seem to corroborate observations made from UAV imagery during the eruption (Fig. 5a, b) and reinforce the importance of microtopography for lava flow impact in urban areas.

##### West of road LP-2

West of LP-2, the heterogeneity of building and roof typologies prevented the extrapolation of discrete impact observations to all observed houses. Consequently, there is a larger uncertainty regarding building structures and materials, which should be considered when inferring impact mechanisms. Structures DSLP16, 19 and 22 are entirely inundated, and only fragments of the highest structures are visible at the flow surface and were possibly rafted away from their original locations (Fig. 8a). Structure DSLP13 was affected by lateral pāhoehoe breakouts that did not inundate the inside of the houses. Based only on lava flows, DSLP13 would be assigned a DS 1, but the combination with pre-flow impacts (Fig. 6e) with the appearance of cracks related to neither lava flow nor tephra load suggest a DS 2–3 (Fig. 8b).

DSLP17, being an aggregate of structural units with various roof typologies, illustrates a variety of impacts and associated mechanisms. Figure 8c, showing the right part of the building on Fig. 2f captures, from near to far, (i) the partial roof collapse of a 2-pitched roof from the translation of the fibre-cement tile panels on its primary metal structure; (ii) the total, inward roof collapse of a flat roof due to the failure of the connection of primary metal beam to the supporting wall and (iii) the total collapse of the main 2-pitched roof from the collapse of the entire roof structure. Assigning a single mechanism to these observations is difficult, especially since the roof typology could not be entirely inferred (i.e. no secondary beam could be observed), but impacts seem to be the result of a combination of tephra load (i.e. horizontal cracks), thermal effect (i.e. softening of metal beams) and lava flow pressure (i.e. wall deformation). The lateral stress from lava flows comes both from the outside (i.e. bulging walls on Fig. 8c, d) and from significant ponding that occurred inside the building at the junction of the different building parts (Fig. 8d).

Structure DSLP17 also illustrates the possible role of structural aggregates in modulating impact. Figure 8e shows the connection between the farthest wall on Fig. 8c and a structure with a small 4-pitched roof. The space between both structures suggests that this building contains statically independent structures that are dynamically interacting, which typically increases vulnerability to earthquakes (Formisano et al. 2010; Zuccaro and Cacace 2015). Although we observed no evidence of increased impact, a pre‐existing horizontal crack appears to have been reinforced prior to the eruption using vertical metal braces (Fig. 8e), suggesting a pre-existing vulnerability. Figure 8f shows the connection between DSLP17 (left) and DSLP22 (right), the collapse of which caused cracks in the connecting wall. Since this wall’s typology cannot be inferred from outside photographs, it is not possible to infer whether these cracks constitute structural or non-structural impacts. This observation nevertheless highlights the importance of considering structural aggregation in the physical vulnerability of buildings to volcanic hazards.

## Discussion

### Impacts from primary hazards

#### Lava flow

Recent studies on lava flow impacts have emphasised the importance of the flow’s properties (e.g. morphology, thickness) upon initial contact in controlling the impact on buildings (e.g. Meredith et al. 2022). By reconstructing the dynamics of flow emplacement from the field mapping of surface textures, the Corazoncillo case study suggests that buildings were impacted by various phases of the flow, each associated with specific impact mechanisms. Conceptually, we identified four main phases. Firstly, buildings were inundated by a low-viscosity, immature ʻaʻā morphology, aided by the presence of windows and doors on the up-flow side of buildings. During this phase, the thermal effect was the dominant impact mechanism, igniting fires, weakening some structural elements (e.g. metal beams) and melting plastics which, in the case of the oldest buildings, contributed to roof collapse (e.g. Figure 8c, d). Secondly, the urban microtopography combined with flow cooling caused static ponding on the side of and inside buildings. This caused the flow to stall, develop a thick, coherent ʻaʻā’ crust and inflate, resulting in an increase in lateral pressure. On the outside walls, surface decolouration due to thermal impact of this lava morphology is limited, demonstrating efficient thermal insulation (Biass et al. 2019; Harris et al. 2010). Thirdly, pāhoehoe flowing on an inflated ʻaʻā substrate was able to reach roof level and, through a combination of static vertical load and horizontal pressure, caused impact on roofs (Fig. 7). Finally, although late-stage drainage events probably caused negligible direct impact, they contributed to tearing apart already collapsed building elements. Together, these observations highlight the intrinsic dynamic nature of lava flows and the range of associated impacts potentially occurring within a flow that lasted for less than 24 h.

Most buildings suffered inundations > 60%, which correspond to DS ≥ 4 following the scheme of Meredith et al. (2022) (Table 1). Amongst different impact mechanisms and depending upon exposure of the building structure, we dominantly attribute the occurrence of structural impacts to lateral pressure. East of road LP-2, these structural damages are dominantly expressed through diagonal cracks on reinforced concrete pillars (e.g. Figure 7e). Interestingly, no primary structure appears to have suffered any displacement or collapse of the up-flow walls. This is especially remarkable for houses DSLP 2–3, which were the first buildings in the path of the flow and were oriented perpendicular to the main flow direction. No roof impact appears to have resulted in structural impact and consisted mostly of the collapse of the superficial fibre-cement tile panels and, in some instances, of the non-structural walls serving as the roof’s basal structure. Despite widespread inundation of the building’s interiors, no bulging of the roof was observed (e.g. Jenkins et al. 2017), which can be attributed to the short-lived nature of the flow that limited inflation. West of road LP-2, structural damages are more widespread, for which we postulate two main reasons. Firstly, their heterogeneity in building material and age often results in the aggregation of structural units with complex shapes, which appears to increase the physical vulnerability to the lateral pressure caused by lava flows. The second component is the spatial distribution of buildings, both in relation to each other and to the slope. East of road LP-2, buildings are regularly spaced and surrounded by terraced parcels that are surrounded by concrete walls. In contrast, buildings west of road LP-2 are relatively closer to each other and built to accommodate the underlying slope. The increase in terrain slope between road LP-2 and the buildings might have caused a flow acceleration that potentially increased the lateral pressure.

The Corazoncillo case study also illustrates the role of urban microtopography in modulating the emplacement and associated impact of lava flows. The 100 m before the first buildings were encountered by the flow display a shallow, NW-oriented slope (Fig. 1c) corresponding to a solar panel complex aligned along an E-W axis (Figs. 2a and 11a). This artificial terrace contributed to funnelling the initial flow towards the northernmost buildings (Fig. 5a) and probably contributed to reducing the flow velocity. Figure 5a also shows the role of the buildings in modulating the flow morphology, from a narrow, crust-free channel with well-developed levees before contact with the urban area to a wider ʻaʻā’ morphology after, revealing how static ponding against obstacles can influence rheological changes. Finally, channelling within urban elements has also played an important role in constraining flow directionality and impact, especially east of road LP-2 where buildings were sufficiently spaced and concrete walls present. This channelling is especially evident on Fig. 4e, where confinement within streets and against walls gave rise to preferential channel orientations aligned with topographic bulges corresponding to the flow accumulation of late-stage drainage events (Fig. 1e).

#### Tephra fallout

Prior to Nov. 25, tephra fallout only caused roof collapse on annexes in Corazoncillo (“Impact at the end of October” section). Based on our reconstruction of the temporal evolution of the tephra blanket, we estimate that without clean-up operations, roofs would have been exposed by collapse probabilities > 70% by the time the flow occurred (Fig. 3c). Although it is impossible to quantify their actual effectiveness, the theoretical framework of Spence et al. (2005) suggests that clean-up operations, even when incomplete, significantly help mitigate roof collapse impacts. Nevertheless, it is important to note that the roof morphology observed east of road LP-2 is improperly characterised by any roof type of Spence et al. (2005), and therefore classification might not coincide with applied fragility curves. In fact, tephra—and in most cases, lava—did not result in any structural roof collapse but only caused the failure of the fibre-cement cladding.

##### Effect of clean-up operations

Clean-up operations can, in parallel, result in some unexpected negative outcomes such as injuries or casualties (Blong 1984; Hayes et al. 2019; Magill et al. 2013) or additional damage from the clean-up process (Blong 1984; Jenkins et al. 2014; Wardman et al. 2012). In the context of long-lasting eruptions, repeated clean-up operations on roofs might, therefore, contribute to increasing physical vulnerability over the long term, either through punctual increases of static load from the cleaning crew (Jenkins et al. 2014) or through mechanical impact or abrasion during clean-up (Blong 1984). This aspect of dynamic vulnerability should be considered when designing clean-up operation procedures. In Corazoncillo, we observed one instance where clean-up operations were the direct cause of a secondary physical impact (e.g. partial wall collapse at house DSLP13; Fig. 6e).

##### Impact of rainfall on roof collapse

Rainfall has been observed to increase the static tephra load (Blong 1984). Theoretical (Macedonio and Costa 2012), experimental (Williams et al. 2021) and empirical (Hayes et al. 2019) investigations have demonstrated that precipitations can increase the load by 18–100% depending on the thickness and the composition of the tephra deposit, rainfall (e.g. both antecedent and of the actual event) and roof properties. During the second field deployment, we observed a secondary structure in Las Manchas—though outside of the Corazoncillo study area—collapsing overnight after rainfall episodes (i.e. Nov. 4–5). The structure, subject to similar tephra accumulations but located south of Corazoncillo along road LP-2, was a shop house with a sheet metal roof on iron beam, which we classify as a medium weak roof (MW; Spence et al. 2005; Table 1). We assessed the load increase due to rainfall on the probability of roof collapse at Corazoncillo by using daily precipitation rates measured at the La Palma airport (Fig. 3a). In our model, we consider that 75% of the rain is infiltrated in the deposit, which is justified by the low to moderate roof pitch and by the absence of fine ash in the deposit (Bonadonna et al. 2023). Following Williams et al. (2021), the additional load caused by rainfall is prevented to exceed 30%. We use a water evaporation rate of 0.93 kg h^−1^ using sandy soils as analogues for tephra fallout deposits (Hellwig 1973) and assume negligible loss of water from flow through the deposit. Results show that, for uncleaned deposits on ST roofs, the intense rainfall episode on Nov. 24 could have increased the collapse probability by 10% (i.e. from 68 to 78%). In the case of a 75% clean-up efficiency and MW roofs, collapse probabilities show an increase from 7 to 31% (Fig. 3c).

Conversely, it is important to note that this approach neglects how rainfall modulates the tephra accumulation as a function of the roof pitch. For roofs in La Palma, Osman et al. (2023) estimate that the sliding angle of a dry, 10-cm-thick scoria deposit on tiles is ~ 35°, which is reduced to 29° considering a *wet* deposit, where it is unclear whether *wet* refers to a partially or fully water-saturated deposit. For a 30-cm deposit, dry and wet sliding angles become similar (i.e. ~ 27–28°). Although providing an empirical framework to quantify deposit sliding from roofs, key questions regarding the deposit’s hydraulic properties (e.g. Baumann et al. 2019) or mechanisms (e.g. deposit sliding vs. surface erosion) still remain unanswered and prevent a comprehensive understanding of the role of rainfall in modulating the impact of tephra fallouts to the built environment.

##### Relevance of existing vulnerability models

The vulnerability model of Spence et al. (2005) links a probability of roof collapse to a static load and, in this sense, only considers the most severe damage state (e.g. DS 4–5; Jenkins et al. 2015; Table 2). In the case of Corazoncillo, roof collapse often resulted only in the non-structural failure of the fibre-cement cladding, which suggests a lower damage state. For instance, considering clay tile, asbestos and metal sheet roofs around Kelud, Williams et al. (2020) classified that impacts requiring the replacement of < 50% and > 50% of the cladding as DS 2 and DS 3, respectively. Any reference to probabilities of roof collapse in our results should therefore be interpreted in the light of this limitation. On the other hand, this observation further demonstrates the limitations of vulnerability models available for quantitative risk analyses on the built environment, which remain rooted in the observation of impacts after well-studied eruptions (e.g. Blong 2003; Hayes et al. 2019; Spence et al. 1996). Although dedicated controlled experiments (Hampton et al. 2015; Oze et al. 2014; Williams et al. 2017, 2021) and theoretical studies (Perelli et al. 2023; Zuccaro et al. 2008) are increasingly being developed to complement dominantly empirical vulnerability models, the 2021 Tajogaite eruption highlights the need of site-specific methodologies to accurately capture the physical vulnerability at the local level to improve the accuracy of quantitative risk analyses.

**Table 2:** Summary of damage state schemes used in this study

		Damage state					
		DS 0	DS 1	DS 2	DS 3	DS 4	DS 5
Structural damage from tephra load (Jenkins et al. 2015)	**Severity**	No damage	Minor/basic repair required	Moderate repair required	Major/specialist repair required		Beyond economic repair
	**Description**	No damage	No damage	No damage to principal roofing supports	Partial or complete failure of the supporting structure, e.g. battens or trusses; partial or moderate damage to the vertical structure		Collapse of roof and supporting structure over 50% of roof area; external walls may be destabilised
Roof damage from tephra load (Williams et al. 2020)	**Severity**	No damage/light damage		Moderate damage	Heavy damage	Severe damage/collapse	
	**Description**	No observable damage		Cladding replaced for < 50% of the roof area	Cladding replaced for > 50% of the roof area	The roof over the central part of the building has collapsed or the building has collapsed	
Building damage from lava inundation (Meredith et al. 2022)	**Severity**	No visible damage	Negligible	Minor	Moderate	Major	Complete destruction
	**Description**	No evidence of structural or non-structural damage	Negligible structural damage; minor surface damage, such as melting of plastic exterior façade	Minor structural or non-structural damage, such as cracking and holes in wall, or < 30% inundated	Partial structural damage or roof destruction, partial destruction of non-structural wall, or 30–60% inundated	Major structural and non-structural damage; wall, column, beam or roof collapse, or > 60% inundated	Complete structural destruction or burial

### Compound impact sequence

**Fig. 9:**
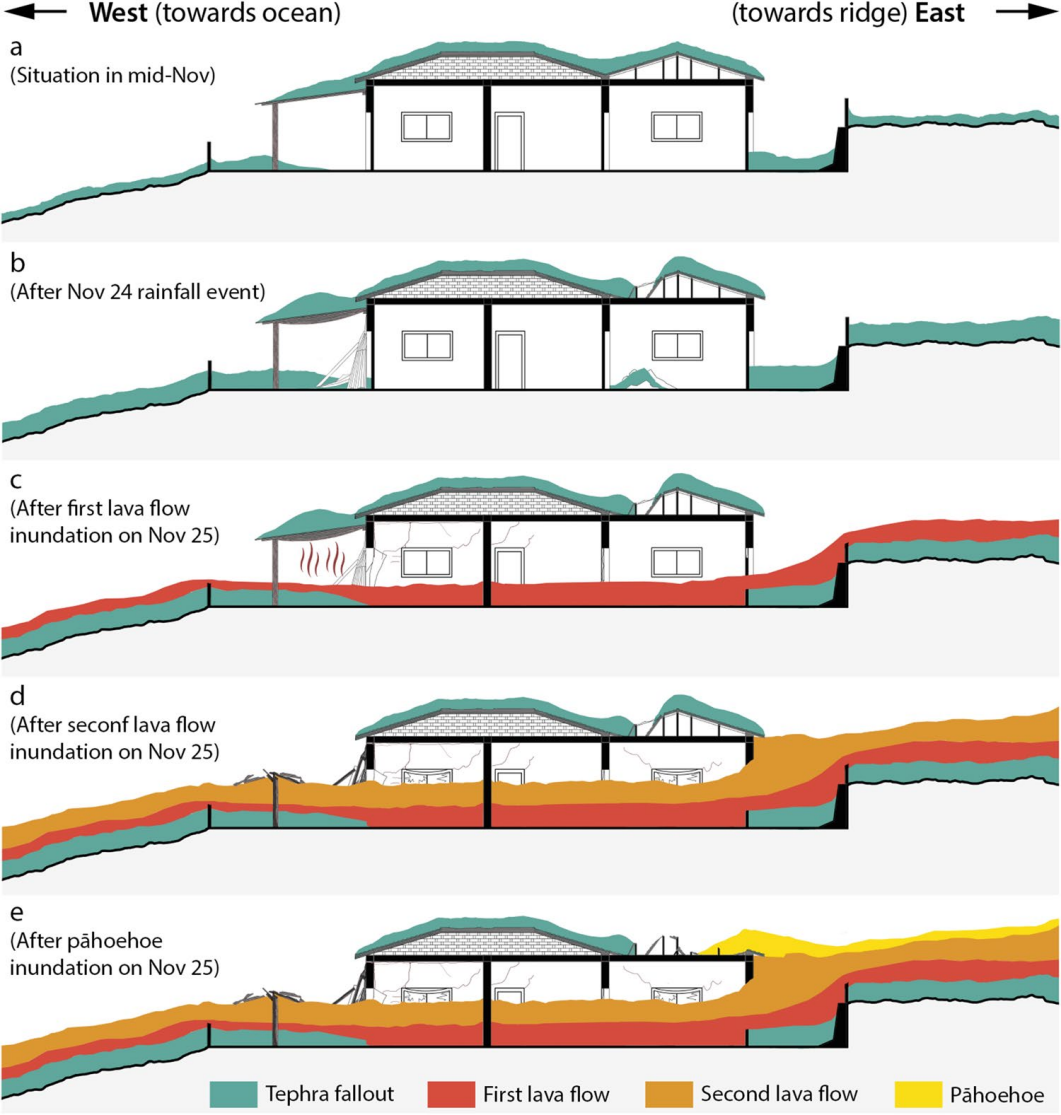
Schematic reconstruction of the compound impact sequence associated with the Nov. 25–26 flow

Compound impacts relate to the interaction of multiple hazard phenomena (Fink and Ajibade 2022; Catto and Dowdy 2021; Zscheischler et al. 2018), the sequence of which will modulate the impact mechanisms. Based on observations made around Corazoncillo, we reconstructed a conceptual impact sequence accounting for the compounding of impact mechanisms related to tephra fallout and lava flows (Fig. 9).

1. Between eruption onset and lava flow inundation on Nov. 25, repeated tephra accumulations affected Corazoncillo. Our results suggest that clean-up operations from circa Oct. 9 prevented the static tephra load from exceeding ~ 3.2 kPa (Fig. 3b). It is possible that, in some instances, clean-up operations might have simultaneously increased physical vulnerability of some buildings by an unquantified factor (Fig. 9a).
2. On Nov. 24, rainfall of ~ 50 mm affected Corazoncillo and likely had the simultaneous effect of increasing the static load and removing tephra from pitched roofs. Due to the increase of static load, the non-structural failure of some fibre-cement cladding in zones of tephra overthickening occurred (i.e. trough located at the junction of two opposite-sloping roof pitches; Fig. 9 b).
3. On the morning of Nov. 25, a fluid, immature ʻaʻā flow inundated Corazoncillo. Due to its low viscosity, the flow was able to penetrate through openings and inundate buildings, triggering fires (Fig. 9c). The most widespread thermal impact occurred to wooden elements (e.g. pillars and rafters supporting porches). Relationships inferred from flow textures suggest that despite weakening the wooden elements, porches did not yet collapse. In the rare cases where porches were made of metal (e.g. DSLP18), metal trusses show ductile deformation under the combined action of temperature and tephra load. On the weakest buildings (e.g. DSLP17; Fig. 8c–f), fires might have contributed to roof collapse.
4. Due to changes in flow properties and static ponding caused by urban microtopography, the flow transitioned to an ʻaʻā morphology, developed a coherent crust and inflated, allowing the lateral pressure to become the dominant hazard mechanism over temperature. Along its main axis, the flow inflated up to roof level, causing a sufficient pressure to induce structural impacts to the weakest walls (e.g. Figure 8c). The flow also inflated inside buildings, causing a confinement capable of impacting the reinforced concrete structure of the strongest observed structures (Fig. 9d; e.g. Figure 7b). East of road LP-2, most porches collapsed during this stage of the flow.
5. In the evening of Nov. 25, a new vent opened by the solar panels and emitted pāhoehoe that overtopped the ʻaʻā surface whilst residual movement of the latter still occurred. East of road LP-2, pāhoehoe breakouts were able to inundate roofs, causing the collapse of fibre-cement tile panels under a vertical static load and, in some instance, overturned the walls supporting the cladding (Fig. 9e).
6. Possibly simultaneous to pāhoehoe inundation or short after, drainage of the ʻaʻā flow further tore apart collapsed elements lying on the flow surface.

### Additional observations

**Fig. 10:**
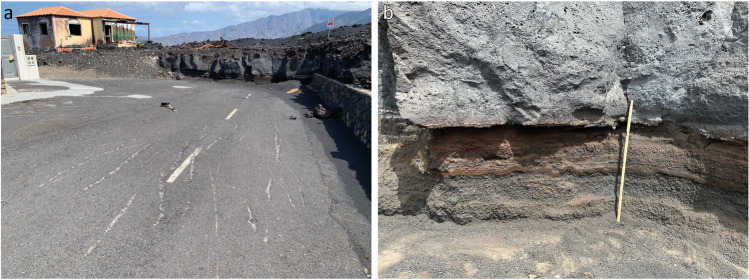
Relationship between the tephra deposit and the lava flow. **a** Cleaned road showing scratch marks from cleaning machinery with DSLP18 in the background; **b** tephra section showing the top of MU thermally altered by the lava flow. **b** is located in the extension of the white dashed line in the road visible on **a**

During a later visit in May 2023, we observed that the lava flow on the road west of the solar panels leading to DSLP2 was bulldozed and removed, exposing the original road surface at the entrance of DSLP18 (Fig. 2a) and creating a vertical section exposing the tephra deposit (up to the top of MU, with the end of MU sedimentation being assigned to 25–27 November; Bonadonna et al. 2022) and the lava flow sequence (Fig. 10). At this location, the thicknesses of the tephra deposit and lava flows are ~ 60 cm and ~ 1.30 m, respectively, with the latter comprising a ~ 1.2-m-thick massive core showing no basal breccia and a ~ 10-cm crust. This section is informative regarding two aspects: the thermal impact of lava flows on roads and tephra compaction that are discussed below.

#### Thermal impact of lava flows on roads

In the current recognition of their non-binary impact (Deligne et al. 2022; Meredith et al. 2022), there is a growing concern about the potential thermal impact of lava flows to bury infrastructure and, specifically for roads, their ability to cause discolouration and desiccation (e.g. Tsang et al. 2020). Figure 10 shows no such impact. The experiments of Tsang et al. (2019) suggest that the maximum temperature reached in a soil substrate at 20-cm depth from inundation of a short-lived, 1-m-thick basaltic lava flow would result in temperatures < 60 °C. At a depth of 60 cm, lava flow inundation caused virtually no temperature increase for dry soils and a marginal (~ 10 °C) increase for wet soils, which is attributed to the development of convection cells (Baker et al. 2015). These experiments suggest that the presence of tephra mitigated the direct potential thermal impact of the lava flow on the road surface, further illustrating the importance of considering the hazard sequence when inferring compound impacts (e.g. Williams et al. 2019, 2021; Pescaroli and Alexander 2018). It is however interesting to note that the only impact to the road surface is scratches made by bucket teeth during clean-up operations (Fig. 10a), providing another illustration of the potential impacts caused by clean-up operations (Wardman et al. 2012).

#### Tephra compaction

Compaction plays a critical role in the preservation of tephra deposits and the estimation of associated eruption source parameters (Engwell et al. 2013). In the case of fine-grained deposits, rainfall has been shown to cause up to 50% compaction (Blong et al. 2017; Tarasenko et al. 2019). Figure 10b illustrates a rapid coverage of the tephra deposit by a metre-thick lava deposit. Although the exact tephra thickness before flow emplacement could not be measured at this location, the post-flow thickness of the tephra deposit corresponding to LU and MU units is about 60 cm, which is slightly larger than the ~ 55 cm estimated from the spatial interpolation of outcrops (Fig. 3a). Although this discrepancy can largely be attributed to the linear interpolation used to estimate the deposit thickness in Corazoncillo, the absence of apparent compaction is striking. Several other interesting observations can be made from this section. Firstly, the tephra does not show physical reworking by the lava flow emplacement, and the contact between the two units is consistently plane parallel. Nevertheless, the top 1–2 cm of the tephra is welded, indicating a thermal effect. Secondly, despite negligible compaction, we notice a disturbance of the finer layers (e.g. MU1 and MU4; Bonadonna et al. 2022). These layers display a wavy pattern with constant thickness that did not appear to have caused any disturbance to the surrounding units. Based on our observations, we cannot unequivocally infer whether the added load caused disturbance to the fine layers that was accommodated by the coarser ones or vice-versa.

#### Where did the pāhoehoe come from?

**Fig. 11:**
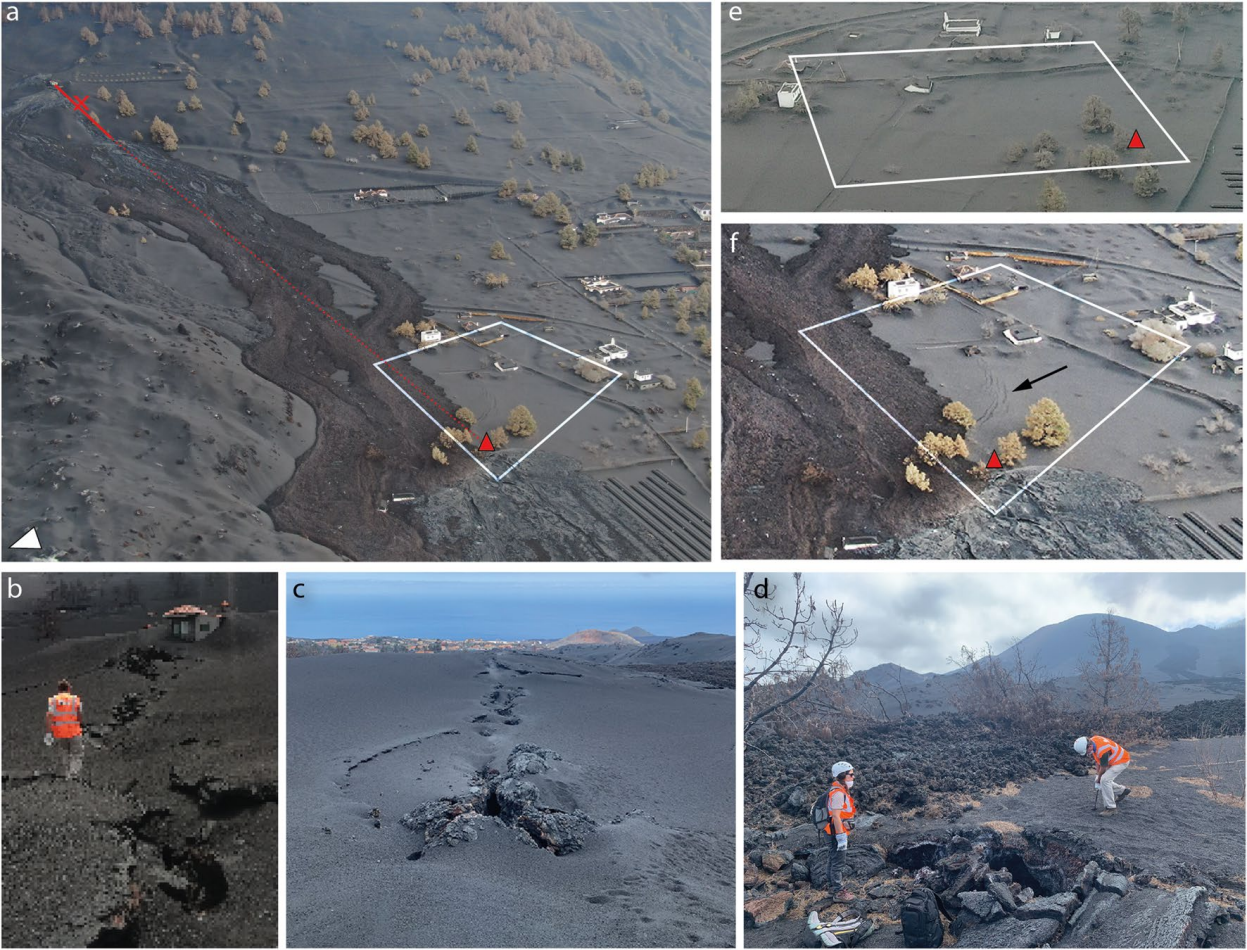
Context and location of the source of the pāhoehoe outbreak. **a** Oblique UAV image taken on Nov. 26 showing the location of the fissure (solid red line) and the extrapolation (dotted red line) to the inferred source of the outbreak. The white arrow indicates the approximate north direction. **b**, **c** Up- and down-flow pictures of the fissure, respectively, taken from the location represented by the red cross on **a**. **d** Inferred source of the outbreak in the field, which is shown as a red triangle on the following pictures. **e** UAV picture taken on Nov. 20. **f** Zoomed into **a** with enhanced contrasts revealing the presence of cracks (black arrow). **a**, **e** and **f** images come from Cabildo Insular de La Palma (2023), and the white square shows the same approximate area

Figure 11a–c shows the orientation of the fissure associated with the second flow, the direction of which extends to the inferred source of the main pāhoehoe outbreak close to the solar panels (Fig. 11a). The pāhoehoe source is located on the southernmost edge of the ʻaʻā flow and shows oxidised spatters and some small drain back features (Fig. 11d). We argue that this source is connected to the house fissure in some way, either by a late-stage fraction of the lava flowing into pre-existing cracks or by the propagation of the fissure. The second option is favoured since, on the one hand, there is no expression of a crack observed on pre-flow UAV images and, on the other hand, surface features appearing on Nov. 26 UAV imagery might indicate some degree of ground deformation associated with the opening of a new vent (Fig. 11e, f). Time-averaged discharge rates of the lava flow inferred from the MIROVA system (Coppola et al. 2020) for the entire eruption suggest the occurrence of a surge around Nov. 25–27 (Bonadonna et al. 2022). This surge may be responsible for the sequential opening of a series of new vents emitting gas-rich lava, with the pāhoehoe representing the latest part of the surge erupted at lower effusion rates. In addition, we discard the possibility of the pāhoehoe originating from the edge of the second flow as this would have either implied a source in the core of the ʻaʻā, which typically results in toothpaste or slabby pāhoehoe (Rowland and Walker 1987) or the development of a nascent tube system, which is unlikely for such short-lived ʻaʻā flow, especially without topographic constraint.

#### Other sources of impact

Although the entire eruptive sequence was accompanied by earthquakes and gas emission, we did not observe any evidence pointing to impact mechanisms associated with these primary hazards. The only evidence of impact related to neither tephra nor lava is illustrated by the cracks in DSLP13 (Fig. 8b), which was interpreted as the result of ground subsidence. However, the absence of information on ground movement throughout the eruption prevents us to infer direct causality to a specific event causing this impact.

## Conclusions

This study combines a detailed mapping of both hazards (e.g. spatiotemporal evolution of the tephra load accounting for clean-up operations and rainfall, dynamics of lava flow emplacement) and the building stocks (both physical vulnerability and impacts) at the scale of a small neighbourhood to explore the interaction of multiple hazards that modulated the overall impact associated with the 86 day-long Tajogaite eruption. Observations allowed for the reconstruction of an impact sequence that highlights the complexity of implementing efficient risk mitigation actions during volcanic crises associated with long-lasting hybrid eruptions.

Regarding the tephra hazard, repeated clean-up operations efficiently reduced the load on structures and contributed to lower the probability of structural collapse. Regarding the hazard from lava flow inundation, our reconstruction highlights the importance of accounting for the dynamics of flow emplacement and the evolution of impact mechanisms as a function of the changes of lava behaviours caused by the evolution of physical properties, which can transition from a dominant thermal effect to lateral stresses and vertical loads. Regarding the compound nature of tephra-lava interactions, although lava flows alone were able to cause impacts beyond repair, field-based evidence shows that some impacts were probably exacerbated by the tephra load. Conversely, tephra fallout deposits were observed to provide an insulating layer that mitigated the thermal impact on road surfaces from lava flows. The relative contribution of each process is, therefore, of difficult quantification.

Although mostly descriptive, this study highlights previously unidentified combination of impact mechanisms resulting from the simultaneous exposure of buildings to two volcanic hazards typically located on two endmembers of eruptive styles and rarely considered together (tephra and lava) and highlights the importance of the hazard sequence when inferring compound impact mechanism from field observations. Our analysis encourages the inclusion of compound hazards within risk assessments and risk management plans to efficiently reduce the potential impact of long-lasting, hybrid eruptions in urban environments.

### Supplementary Information

Below is the link to the electronic supplementary material.

Supplementary file1 (PDF 12752 KB)
