# Supplementary material for: The spatiotemporal evolution of compound impacts from lava flow and tephra fallout on buildings: lessons from the 2021 Tajogaite eruption (La Palma, Spain)
Source: Bull Volcanol. 2024 Jan 9;86(2):10. doi: 10.1007/s00445-023-01700-w (PMC10774154; doi:10.1007/s00445-023-01700-w)

Online Resource 1

The spatiotemporal evolution of compound impacts from lava flow and tephra fallout on buildings: lessons from the 2021 Tajogaite eruption (La Palma, Spain)

Sébastien Biass[1], María-Paz Reyes-Hardy[1], Christopher Gregg[1, 2], Luigia Sara Di Maio[1], Lucia Dominguez[1], Corine Frischknecht[1], Costanza Bonadonna[1], Nemesio Perez[3, 4]

[1] Department of Earth Sciences, University of Geneva, Geneva, Switzerland
[2,1] Department of Geosciences, East Tennessee State University, Johnson City, USA
[3] Instituto Volcanológico de Canarias (INVOLCAN), San Cristóbal de La Laguna, Tenerife, Canary Islands, Spain
[4] Instituto Tecnológico y de Energías Renovables (ITER), Granadilla de Abona, Tenerife, Canary Islands, Spain

Correspondence:
Sébastien Biass (sebastien.biasse@unige.ch)

This supplementary information provides a compilation of field observations of i) house typology, ii) hazards metrics and sequence and iii) impact mechanisms for the buildings described in the main manuscript. Building nomenclature follows the definition provided in Figure 2a of the manuscript. For each house, we provide the damage state inferred from the scheme of Meredith et al. (2022) summarised in Table 2 of the manuscript. Each house also comprises a collection of pictures taken during the Oct 2021 (during the eruption, pre lava-flow inundation) and Nov 2022 (after the eruption).

Name: DSLP-2
Location: 28.608674, -17.882533

Damage State: 4
Date: 17-05-2022

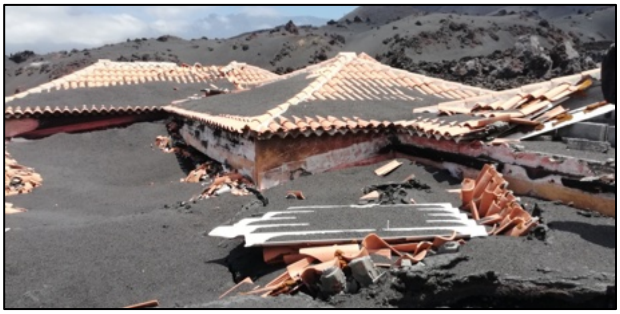

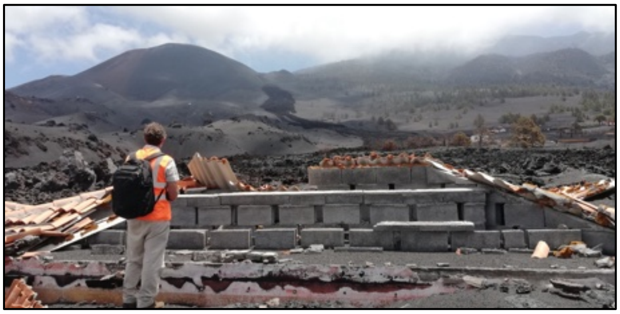

Footprint of the property:

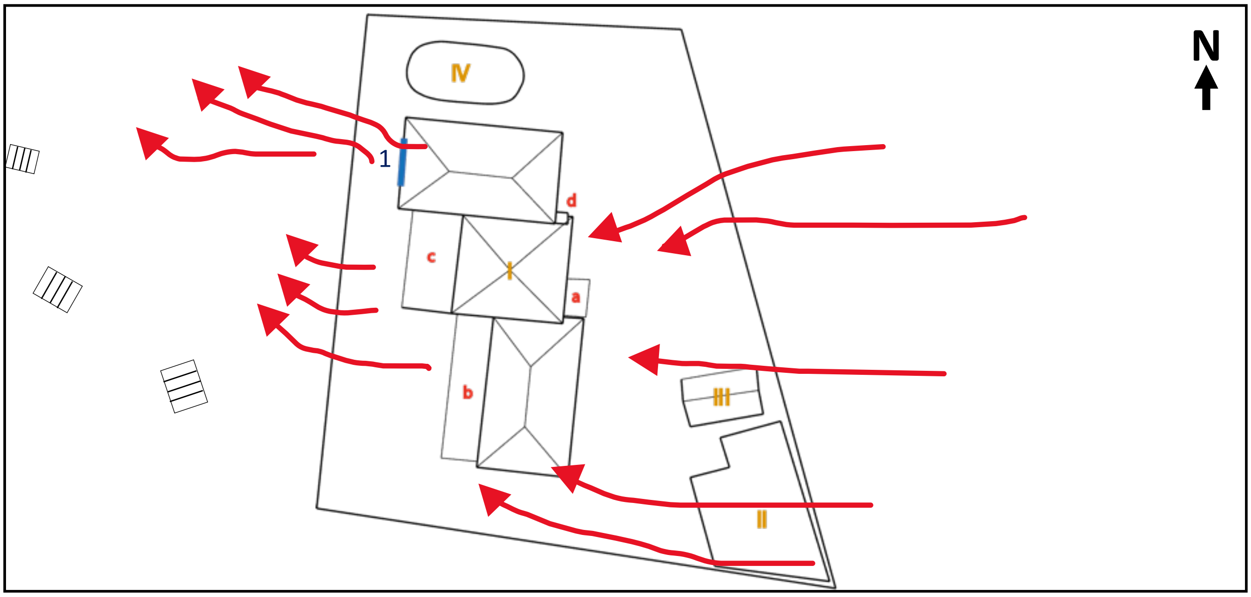

Type of structures in the property: I) Primary (main house) with accessory roofs (a,b,c,) and a chimney (d), II) Secondary (Garage), III) Secondary (storage room), IV) Pool

N° Storeys: I) 1, II) 0, III) 1

Openings:
I) Main house: - 1: 259 cm long, pahoehoe lava up to the top of the window
* It was not possible to measure the other openings of the house since they were completely covered by lava
* 50 cm deflated pahoehoe measured from the top of the ceiling to the top of the collapsed accessory roof (b)
* A'a inside the house and pahoehoe outside
* Roof of the main house of corrugated composite material and tiles supported by cement blocks collapsed by pahoehoe
* Accessory roof (b) moved downstream by pahoehoe lava flow

Construction materials: I) AT2R1, II) AT0W0, III) AM0W0

Age of construction: New

Lava type: A'a and Slabby Pahoehoe

Lava thickness: ~ 3 m

Temporal order: Pahoehoe on top of A'a

Damage: Flow impacting from the E to the front of the main house (up to 75 cm on the roof). First floor 50-70% flooded by lava, only the ceiling can be seen. A'a lava from the E, NE and pahoehoe from S-SE of the house. Lava comes out through openings of the W side of the house. No major structural damage but there are fractures in the corners of the walls and the chimney. Fire damage in the interior and heat removes exterior paint from the house leaving the wall white. Burnt wooden beams and composite material and tiles of the accessory roofs collapsed (a,b,c). Garage (II), storage room (III) and Pool (IV) completely covered by lava.

28/10/2021:

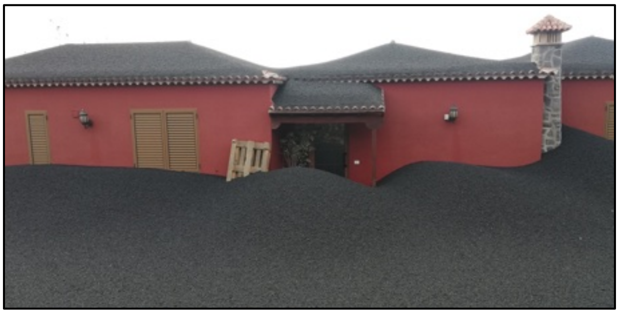
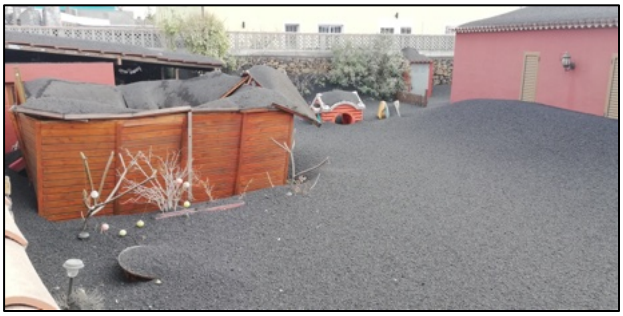

17/05/2022:

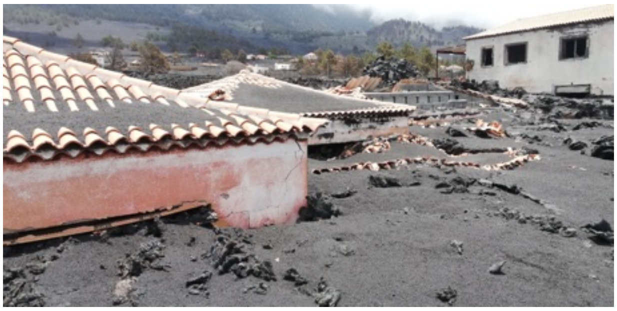
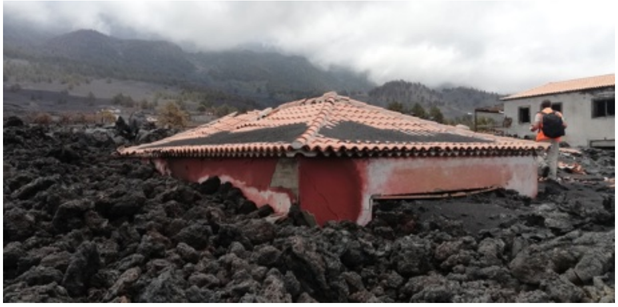
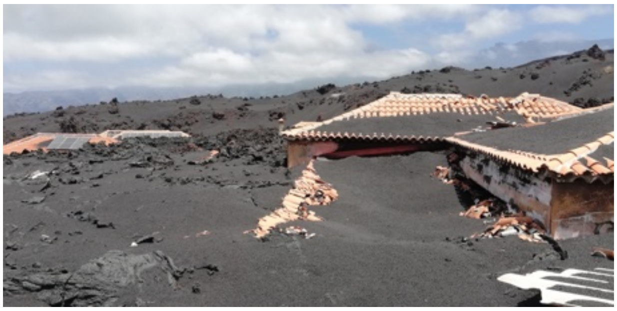
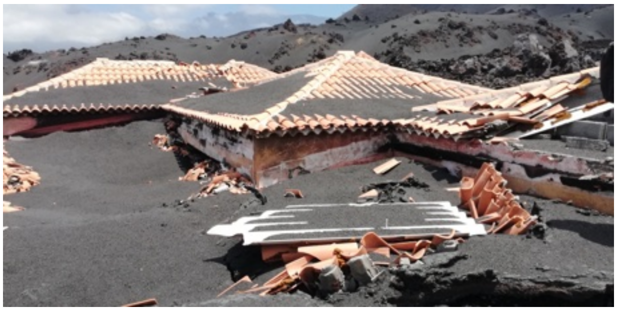
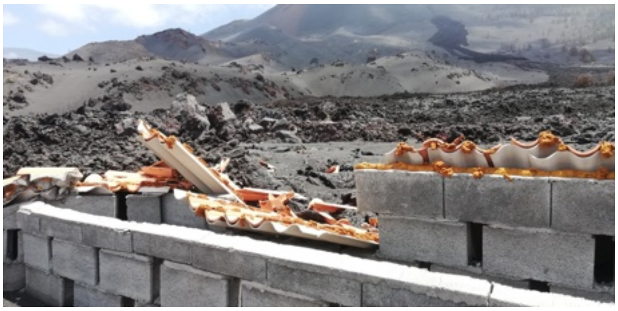
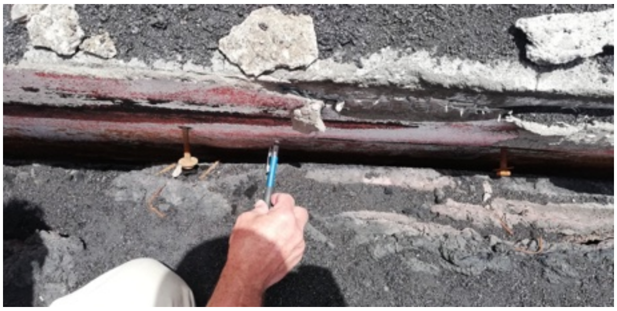

Name: DSLP-3
Location: 28.60915, -17.882586

Damage State: 5
Date: 17-05-2022

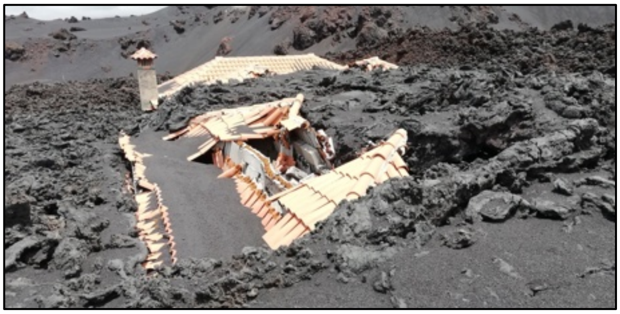

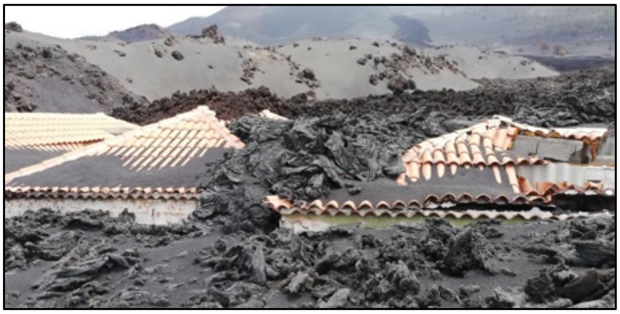

Footprint of the property:

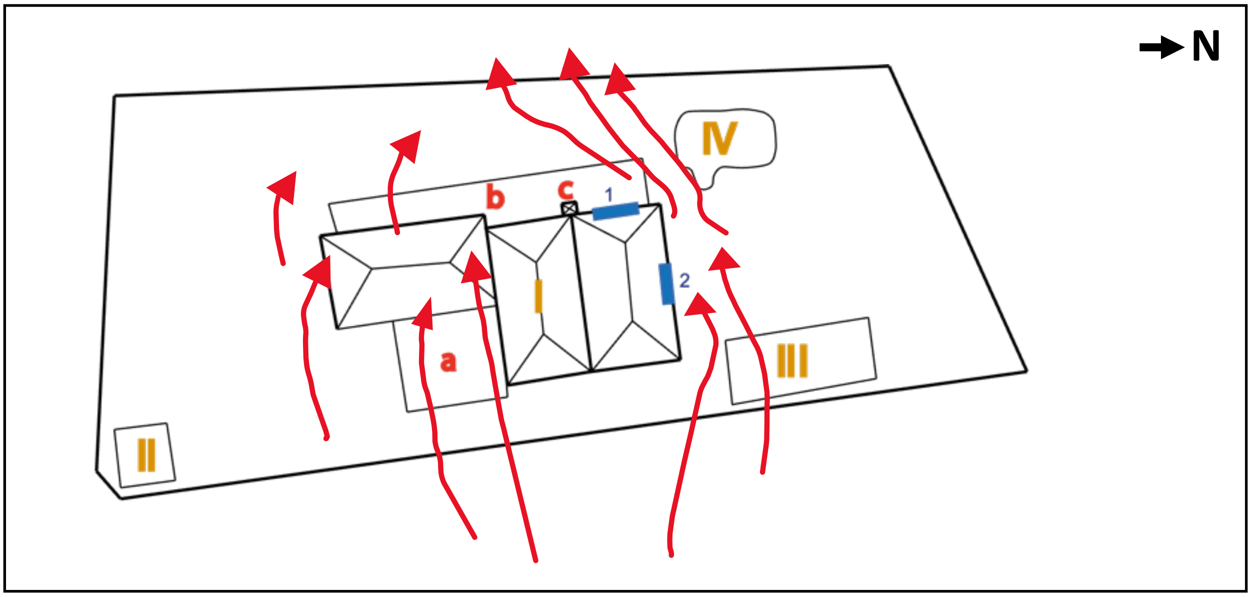

Type of structures in the property: I) Primary (main house) with accessory roofs (a,b) and chimney (c), II) Secondary (?), III) Secondary (under construction in february), IV) Pool

N° Storeys: I) 1, II) 1, III) 1

Openings:
I) Main house: - 1: 199cm long, - 2: 150 cm long

* The pressure of the lava could have helped to destabilize the roof and together with the accumulation of tephra it collapsed.
* The double load where the pitches of the two northern roofs meet could have caused it to collapse since its shape allows for greater accumulation.

Construction materials: I) AT0R1

Age of construction: New

Lava type: Slabby pahoehoe and A'a

Lava thickness: ~ 3 m

Temporal order: Pahoehoe on top of A'a

Damage: A'a flow impacting from the N and NE and Pahoehoe from the E, S and W to the main house. First floor completely flooded by A'a lava and pahoehoe on top of it. Structural damage of the chimney and fractures in the walls. Fire damage in the interior and heat removes exterior paint from the house leaving the wall white. Accessory roof "a" completely covered by pahoehoe lava and "b" collapsed. Secondary structures (II, III) and pool (IV) completely covered by lava.

28/10/2021:

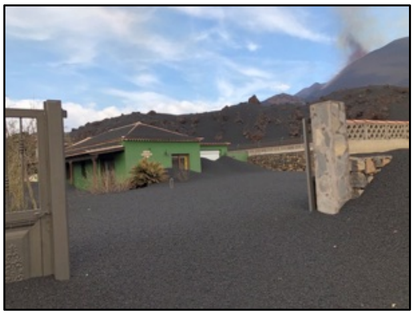

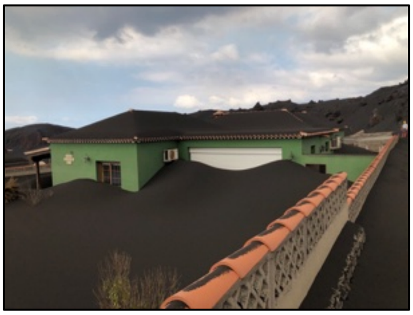

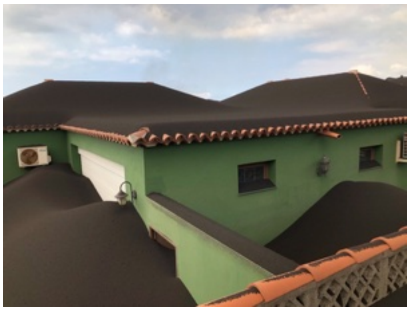

17/05/2022:

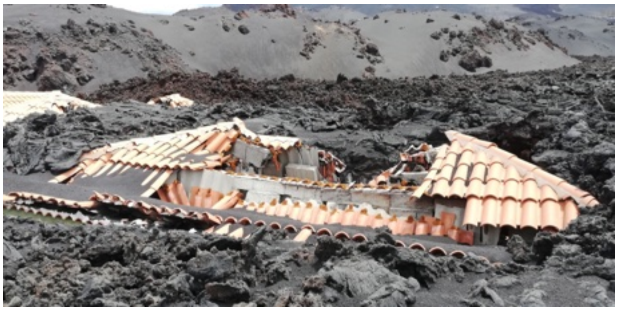

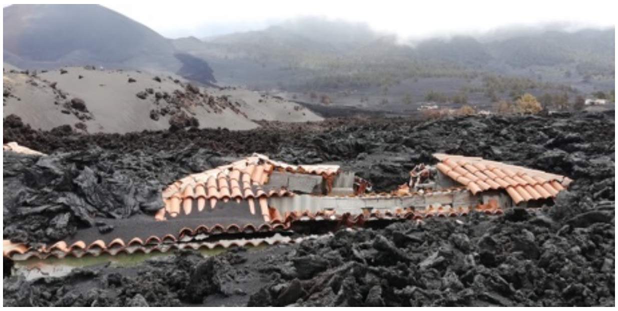

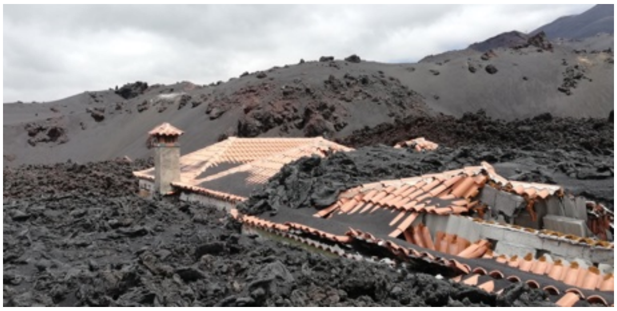

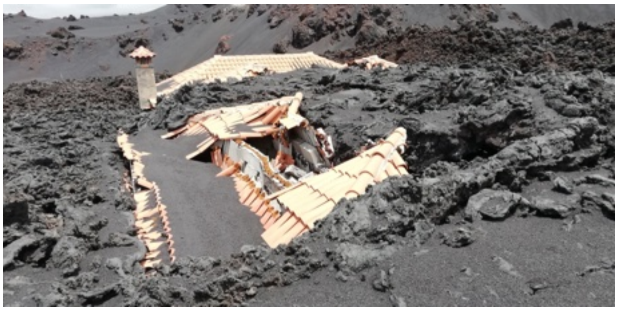

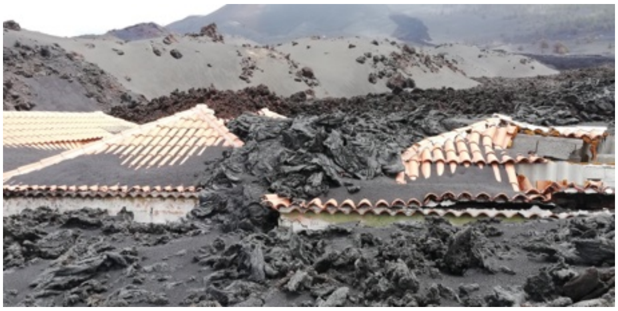

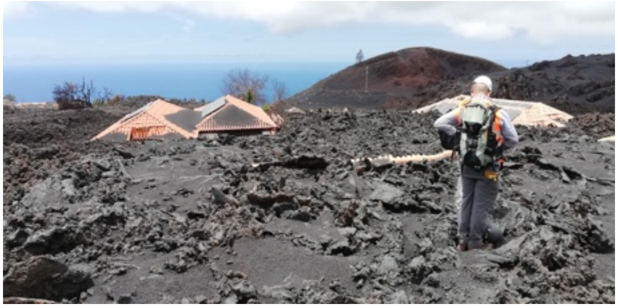

Name: DSLP-4
Location: 28.609022, -17.88281

Damage State: 5
Date: 17-05-2022

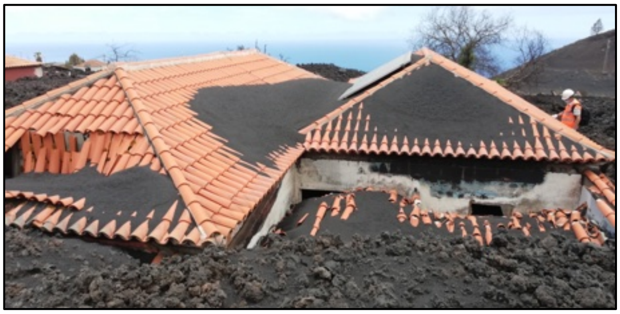

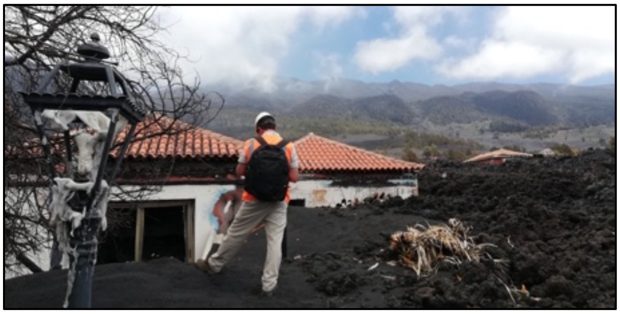

Footprint of the property:

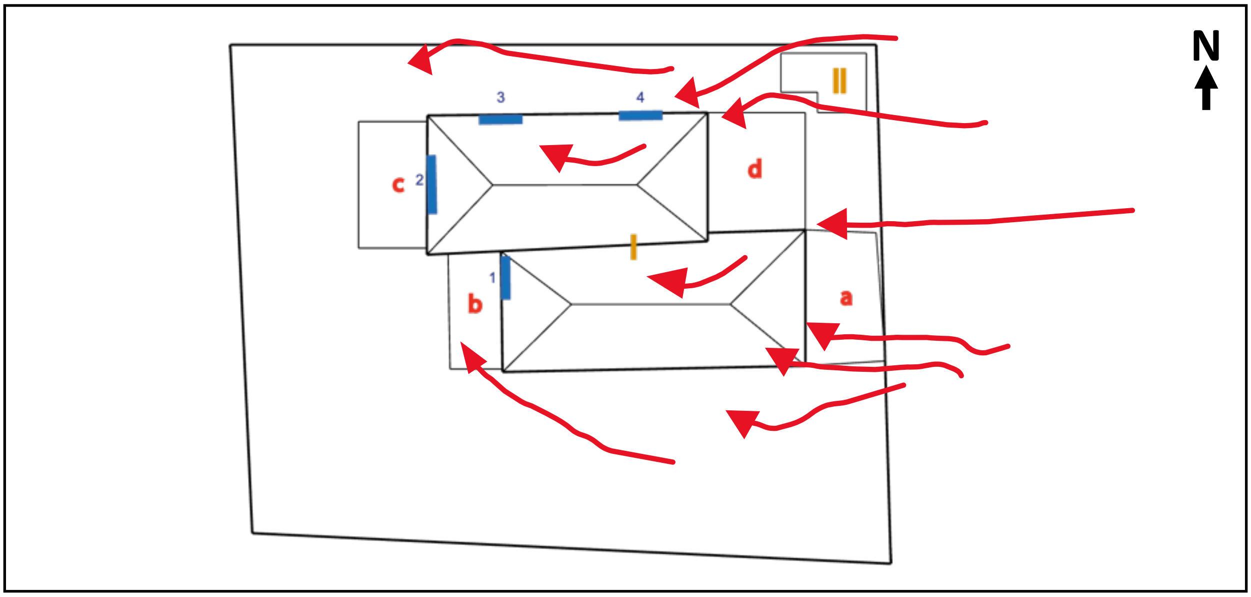

Type of structures in the property: I) Primary (main house) with accessory roofs (a,b,c,d), II) Secondary (?)

N° Storeys: I) 1, II) 1

Openings:
I) Main house/ First floor: - 1: 90 cm with 19 cm from its top to the top of the A'a lava,
- 2: 136 cm long with 90 cm from the top of the window to the top of the A'a lava
- 3: ~125 cm long and - 4: 66 cm long (3 &4 free of lava)

*The A'a flow could have been still in motion when the pahoehoe flow passed over it. There could be evidence of this on what was once the street to the south of the house.

* We entered inside this house and could walk as a dwarf.

Construction materials: I) AT0R1

Age of construction: New

Lava type: A'a

Lava thickness: ~ **3** m

Temporal order: only A'a around the house, a little bit farther pahoehoe on top of A'a

Damage: Flow impacting from the E to the main house. First floor completely flooded by lava entering openings E, and SE. Lava comes out through openings of the W, of the house. No major structural damage but fractures in the NW corner of the house, fire damage in the interior heat removes exterior paint from the house leaving some parts of the wall white. Metal frame of sliding door (opening 2) deformed by heat. Burnt wood beams and collapsed fiberglass and tile roof of the accessory roofs (b and c). Accessory roof "d" collapsed by A'a lava pressure. Secondary structure (II) and accessory roof "a" completely covered by A'a lava (at least 60 cm on top of the roof).

26/10/2021 & 30-10-2021:

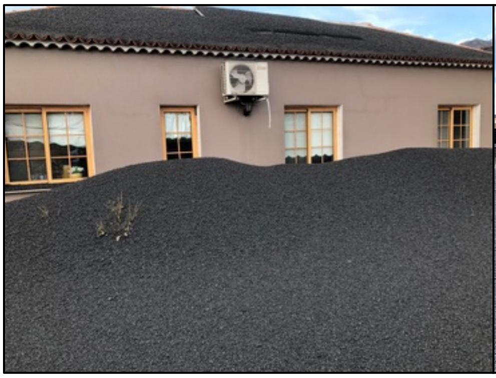
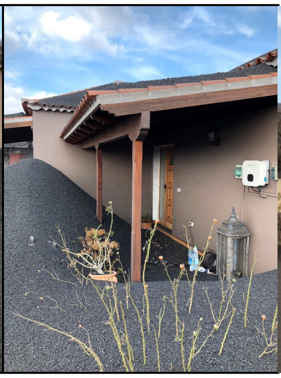
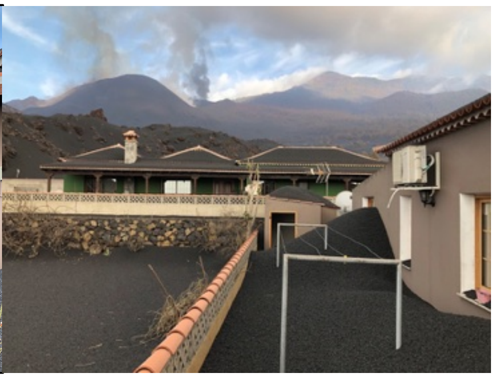

17/05/2022:

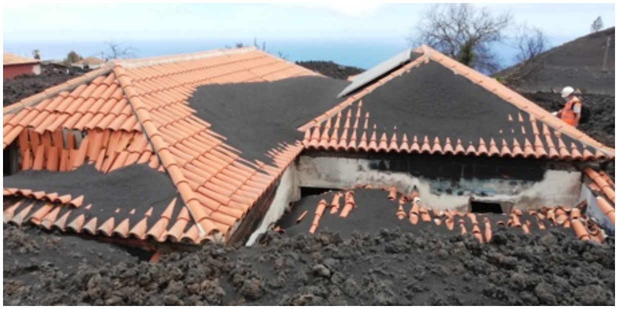
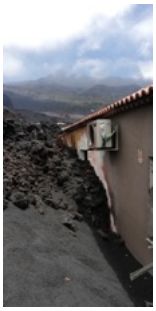
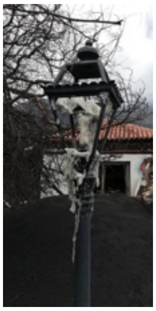
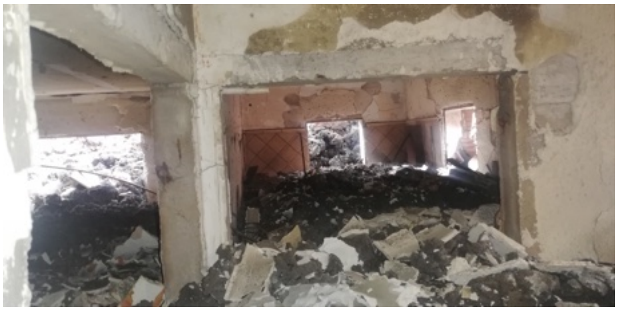
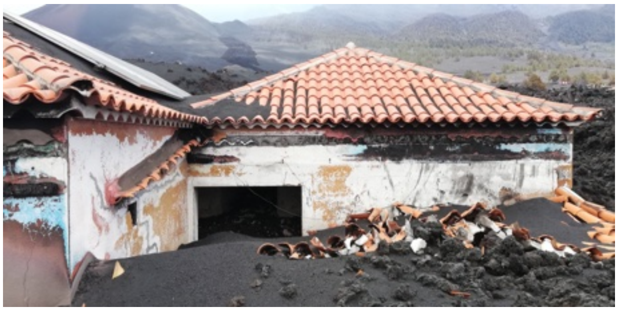
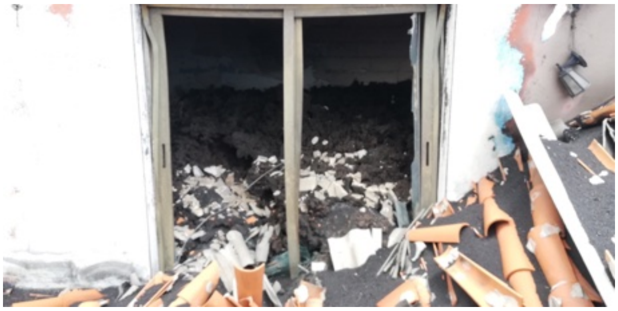
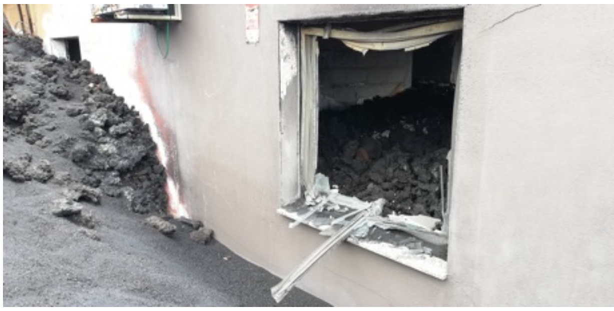
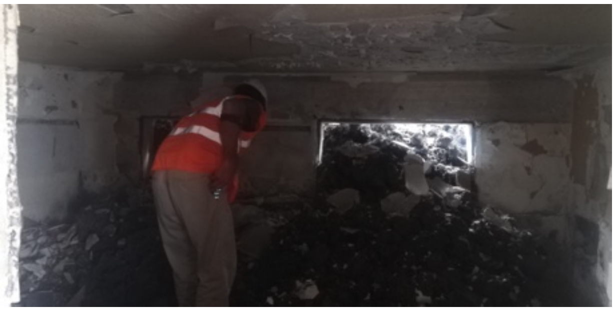
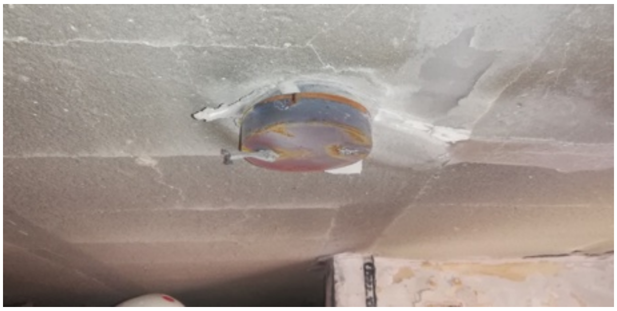

Name: DSLP-5 Damage State: 5
Location: 28.609228, -17.882847 Date: 17-05-2022

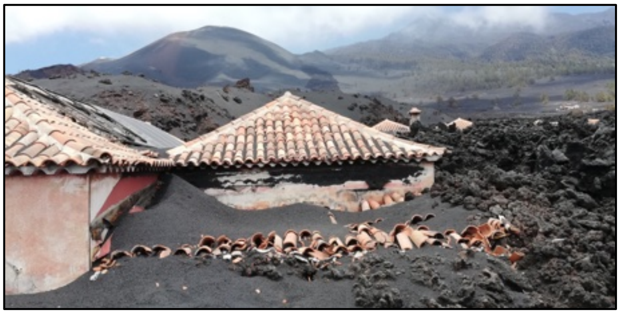

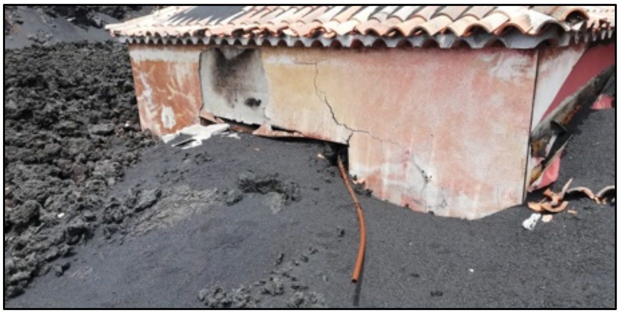

Footprint of the property:

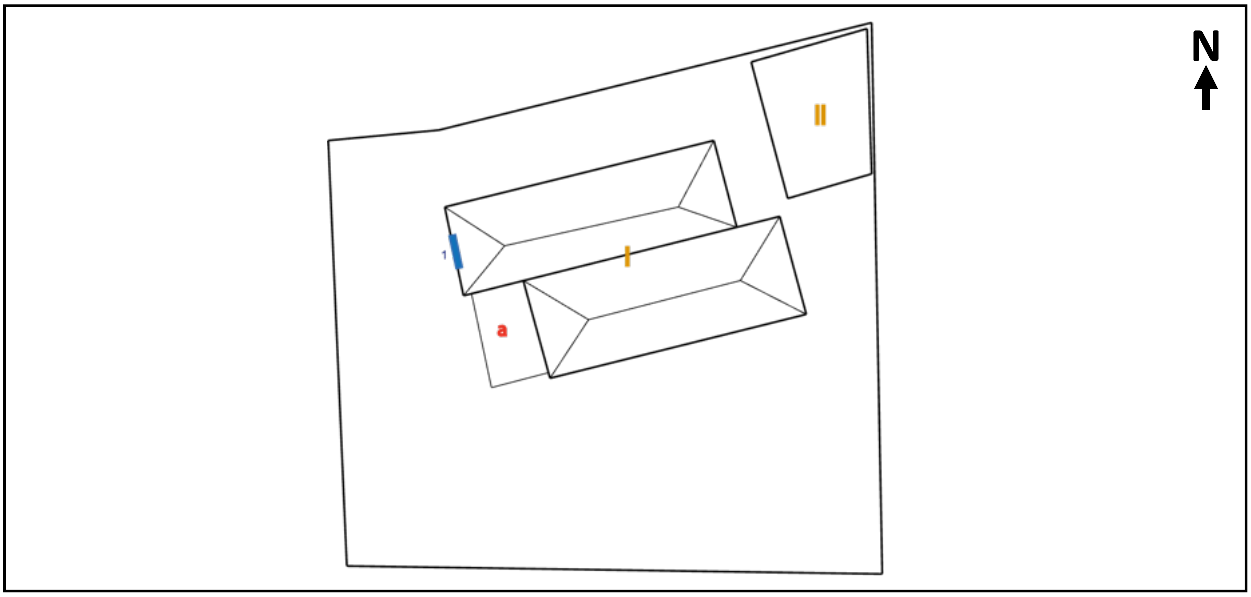

Type of structures in the property: I) Primary (main house) with accessory roof (a), II) Secondary (?)

N° Storeys: I) 1, II) 1

Openings:
I) Main house/ First floor: - 1:lava up to the top

* No openings visible on the north side of the house

*The double load where the pitches (pitch angle of 19-27°) of the two northern roofs meet could have caused it to collapse since its shape allows for greater accumulation. Just under this area there are no cement blocks to hold the fiberglass sheets and tiled roof.

*broken solar panels probably caused by the internal heat of the house (3 out of 8 and 4 out of 8 in both southern pitches of the main house).

Construction materials: I) AT0R1

Age of construction: New

Lava type: Slabby pahoehoe and A'a

Lava thickness: ~2.5 m

Temporal order: Pahoehoe on top of A'a

Damage: Flow impacting from the E to the main house. First floor completely flooded by lava, pahoehoe probably entering S-SE openings. Lava Pahoehoe comes out through W opening (1). Structural damage, fractures and openings in the W walls and fractures in the N side of the house, fire damage in the interior, heat removes exterior paint from the house leaving some parts of the wall white. Burnt wood beams and collapsed fiberglass and tiled roof of the accessory roof (a). Secondary structure (II) completely covered by A'a lava.

26/10/2021:

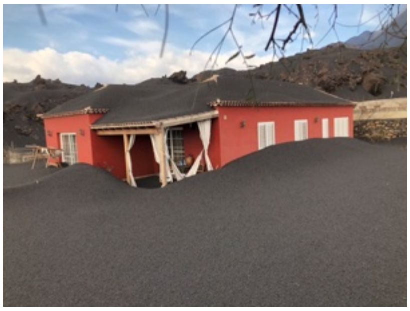

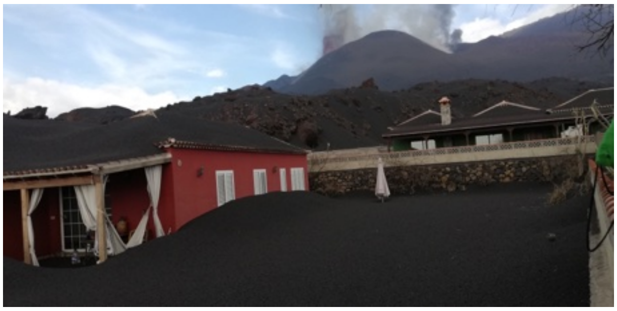

17/05/2022:

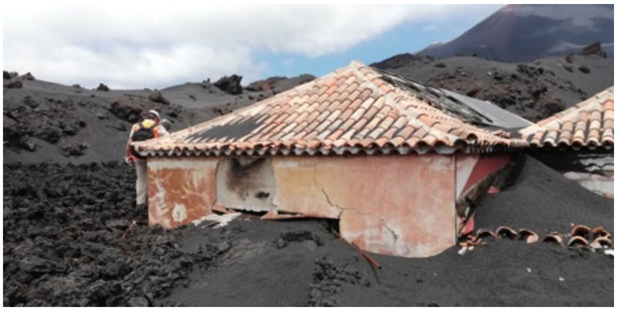

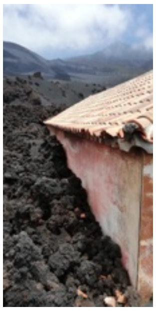

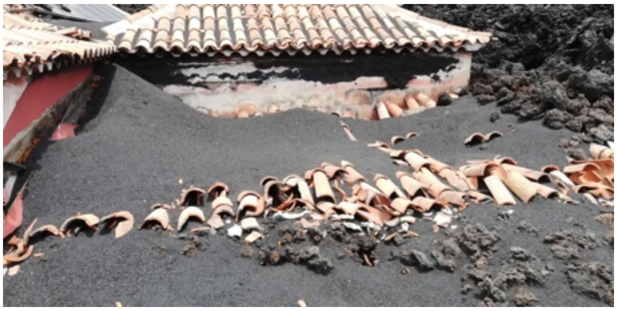

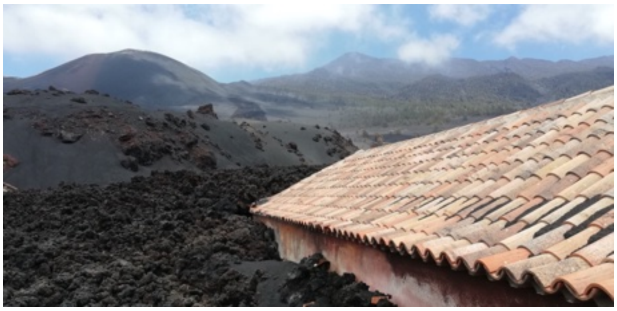

Name: DSLP-6 Damage State: 5
Location: 28.609499, -17.882672 Date: 17-05-2022

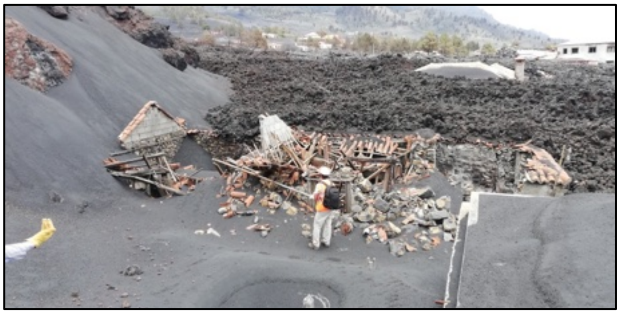

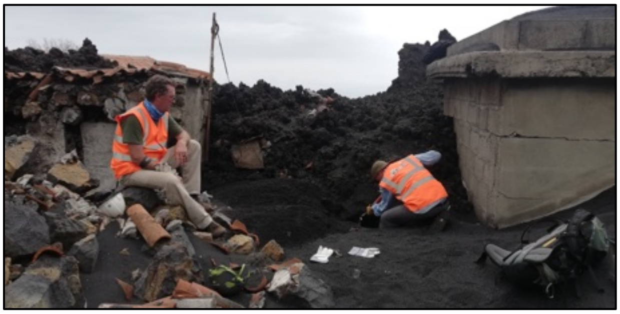

Footprint of the property:

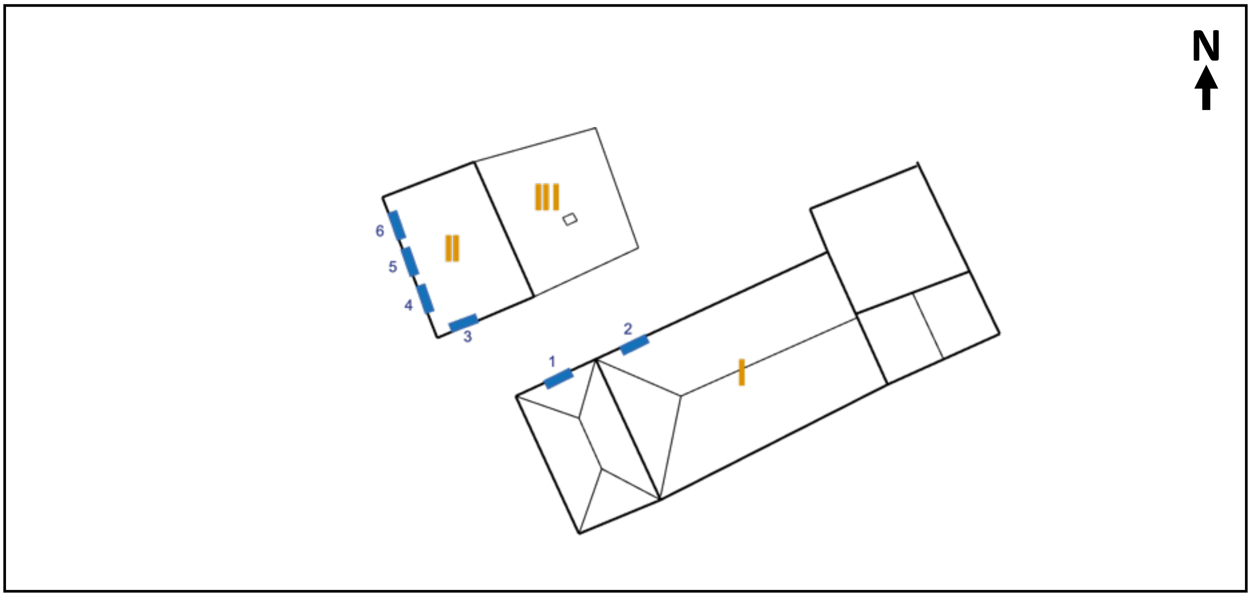

Type of structures in the property: I) Secondary 1 (storage room), II) Secondary 2 (storage room), III) Secondary 3 (well)

N° Storeys: I) 1, II) 1, III) 0

Openings:
I) Main house/ First floor: - 3: 90 cm long, - 4: 90 cm long, - 5: 100 cm long, - 6: 90 cm long
*Broken stone walls ~60 cm wide in contrast to 12 cm concrete block walls
*total stratigraphic column is measured on the roof of structure "II“ (58 cm)
H: 8.5 cm
G:1 cm coarse ash to lapilli + 3.5 cm
E+F: 3.5 cm
D: 2-3 cm
Cfine: 2.5 cm
Ccoarse: 6 cm
B: 12 cm (including Btop)
A: 22 cm

Construction materials: I) AT0I1, II) FC0R1

Age of construction: Old

Lava type: A'a

Lava thickness: ~ 3 m

Temporal order: Only A'a

Damage: Flow impacting from the S to the secondary structure 1. The A'a lava pushes the wall of broken stones and tilts it to the N. Lava does not enter the structure 1 but surrounds it from the S-SW until it reaches the secondary structure 2 and enters it through opening 3. Structural damage, in the northern wall of structure "I", and SE corner of structure "II". Melted cables and opening 1 (wooden door) burned. The pressure of the lava finished collapsing the roof that had previously begun to fall due to the accumulation of tephra and then helped the north wall to collapse.

28/10/2021:

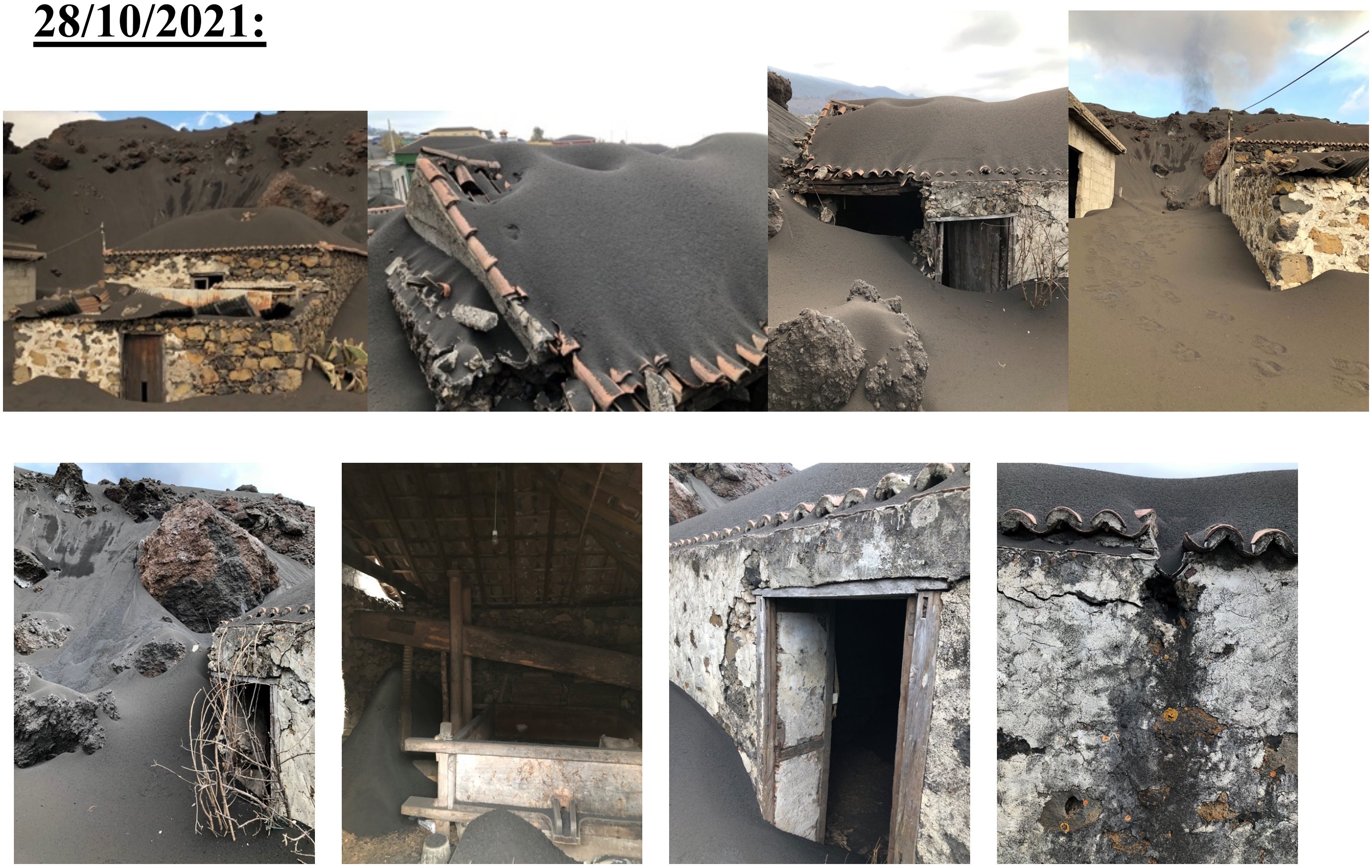

17/05/2022:

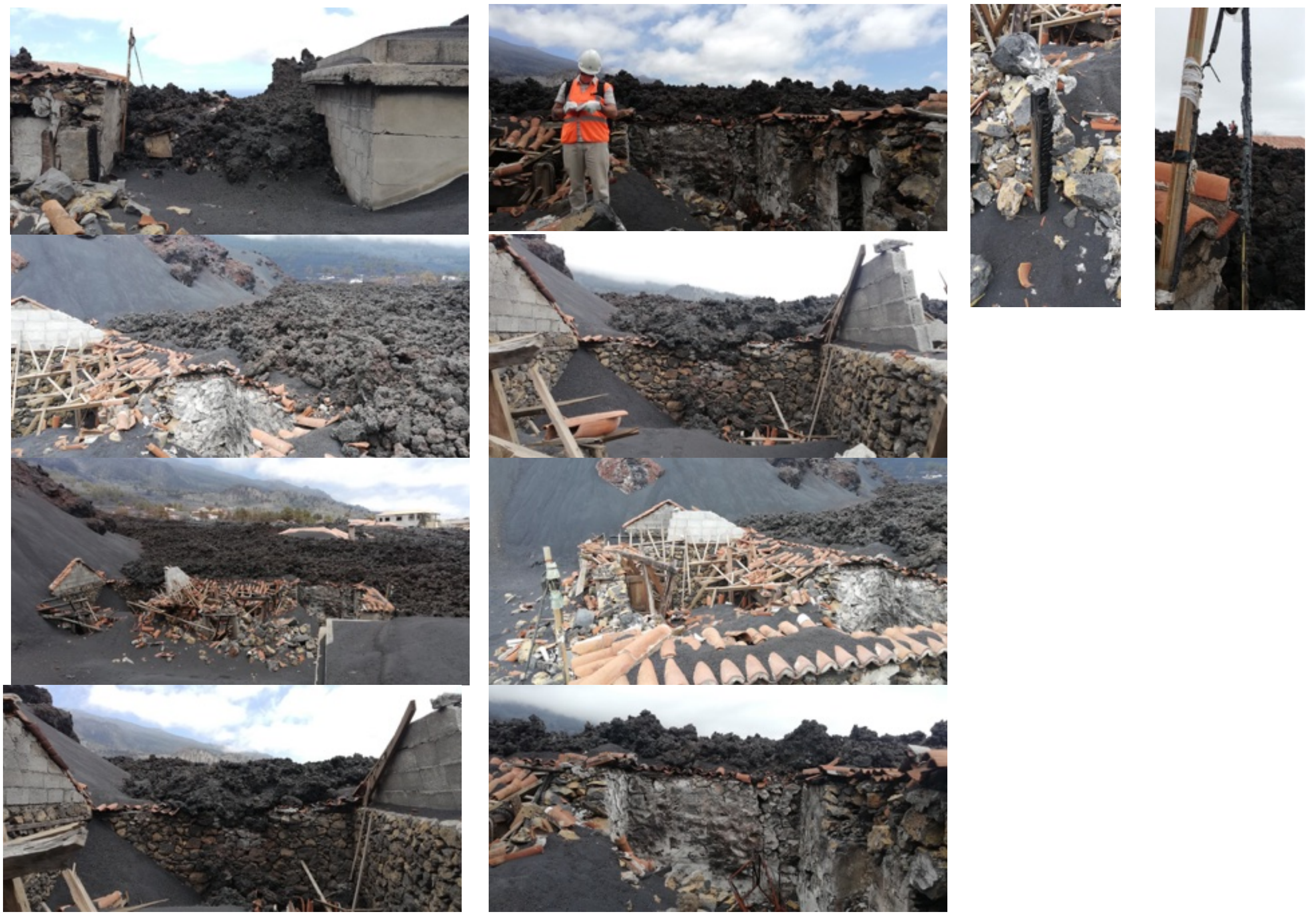

Name: DSLP-7
Location: 28.609616, -17.896451

Damage State:
Date: 17-05-2022

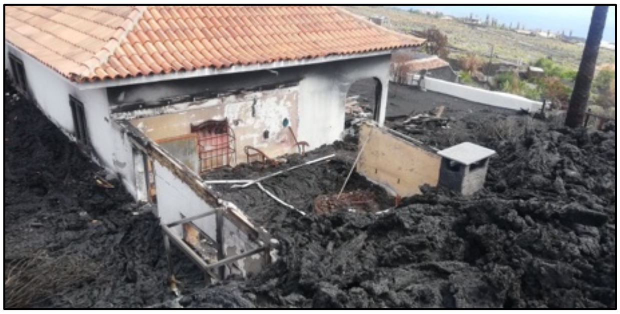

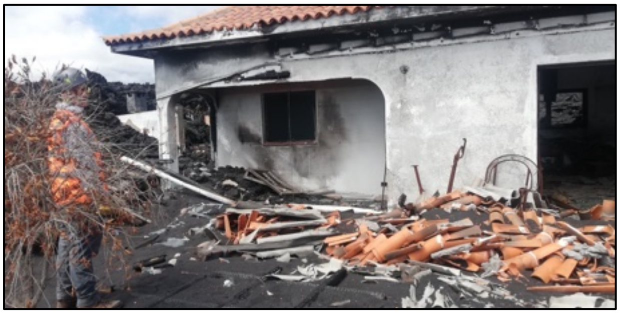

Footprint of the property:

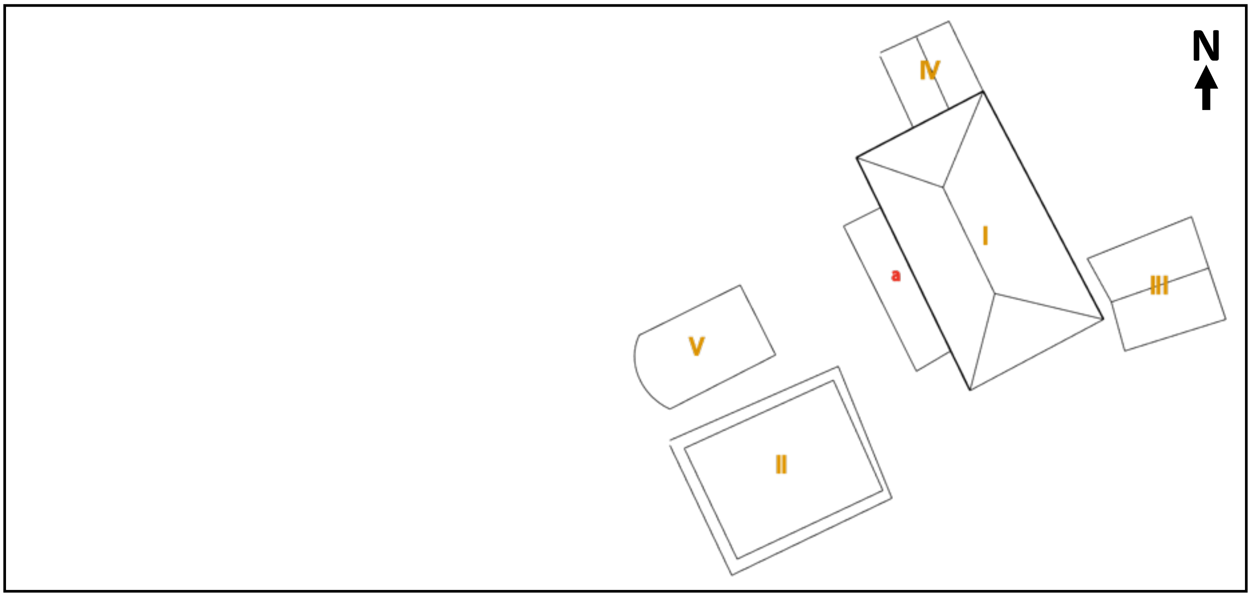

Type of structures in the property: : I) Primary (main house) with accessory roof (a), II) Secondary 1, III) Secondary 2 (Garage), IV) Secondary 3 (barbecue area), V) Pool

N° Storeys: I) 1, II) 1, III) 0, IV) 1, V) 0

Openings:
I) Main house/ First floor:

* point of interest: stratigraphic section that has not been cleaned. Pahoehoe lava over tephra is observed. Section "D" is not complete but there are 6.5 cm below "D" and 18 cm above "D" that apparently reach "G".

Construction materials: I) AT0R1, II) FC0R1, III) AM0E0, IV) AX0R1

Age of construction: New

Lava type: Pahoehoe

Lava thickness: m

Temporal order:

Damage: (T277 field campaign #1 no damaged). Pahoehoe lava floods secondary structure 3. Separation of the lava and the wall of about 3-4 cm is observed, probably produced after lava cooling.

17/05/2022:

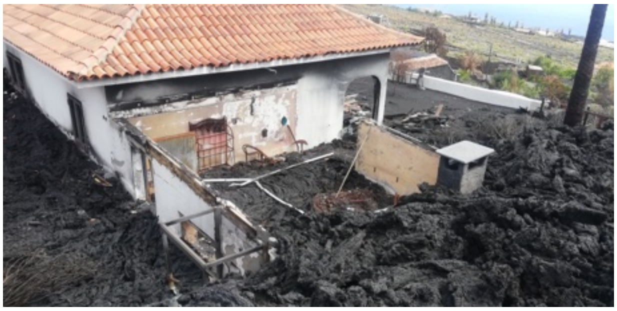

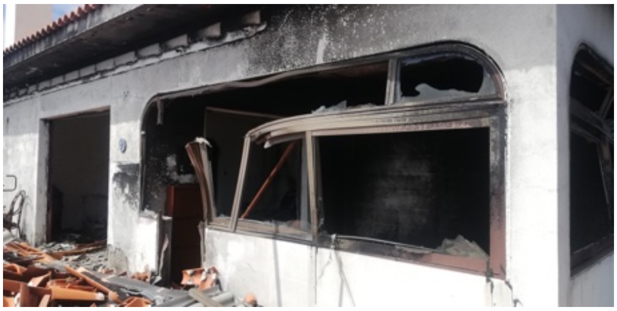

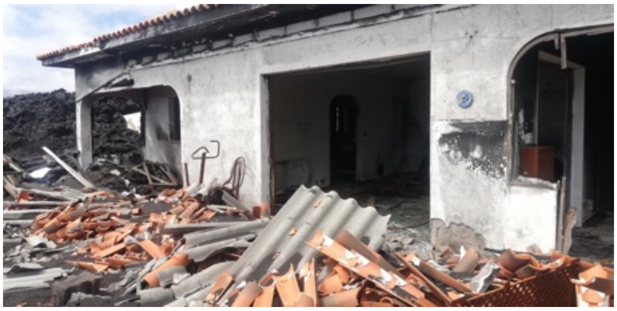

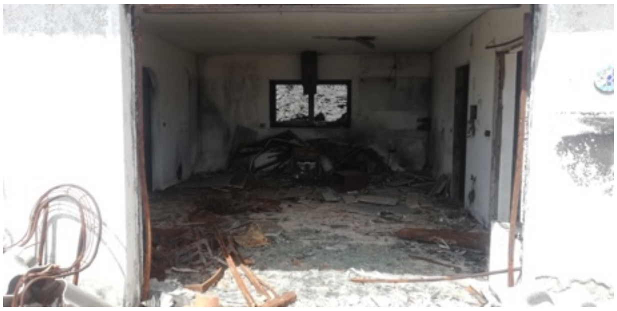

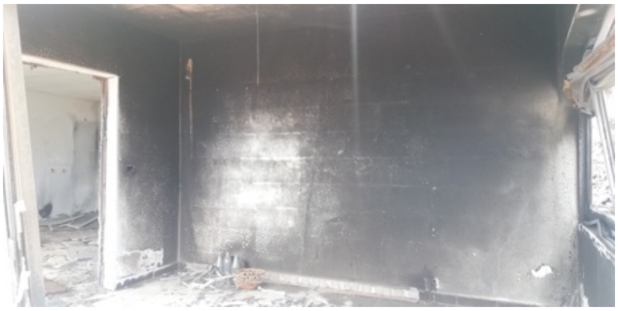

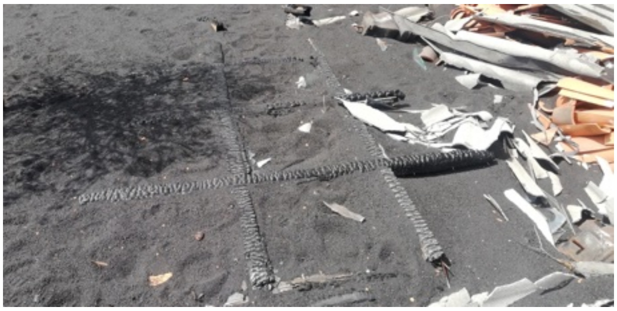

Name: DSLP-8
Location: 28.60881, -17.881182

Damage State:
Date: 18-05-2022

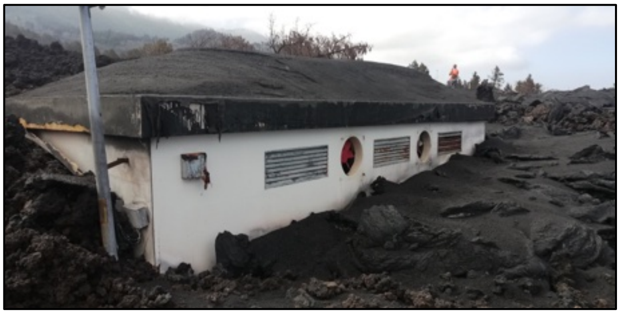

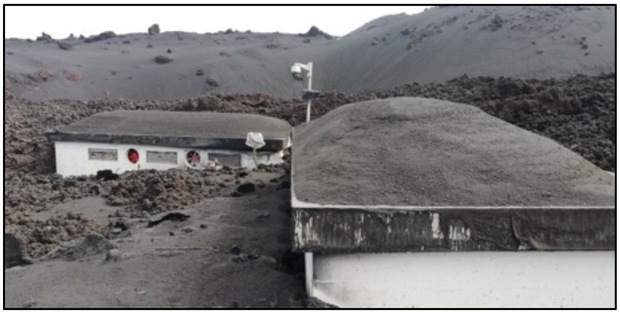

Footprint of the property:

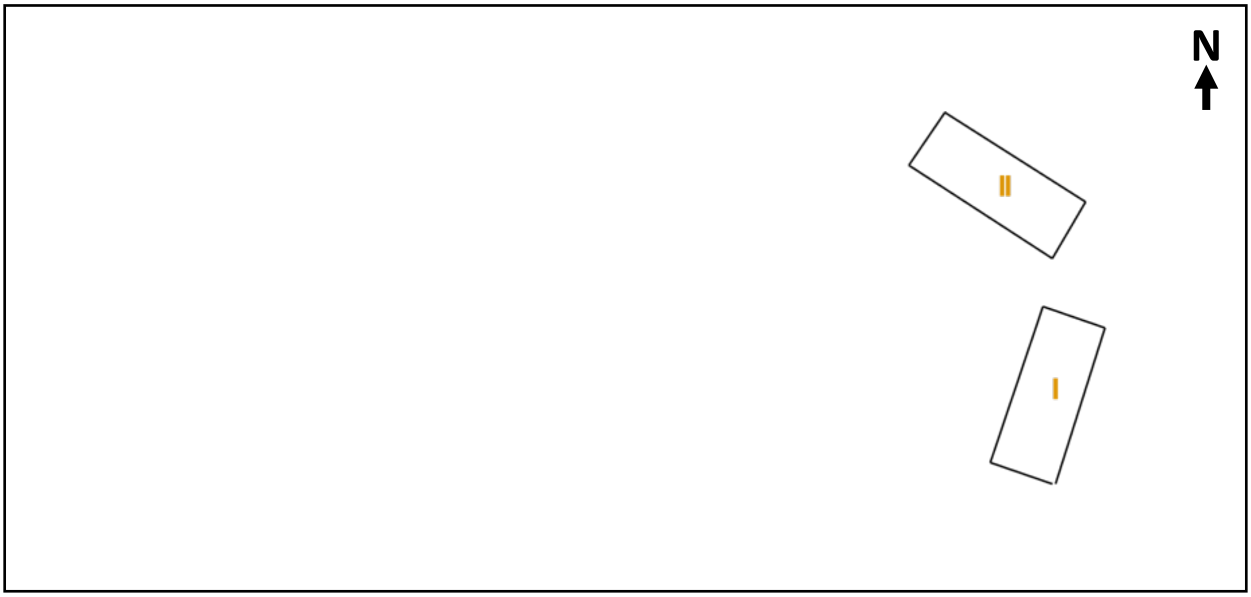

Type of structures in the property: I) Secondary 1, II) Secondary 2

N° Storeys: I) 1, II) 1

Openings:
I) Main house/ First floor:

Construction materials: I) FC0R1, II) FC0R1

Age of construction: New

Lava type: A'a, Slaby pahoehoe

Lava thickness: 1 to 2 m

Temporal order: Slaby Pahoehoe on top of A'a

Damage: The A'a lava flow goes down first and then the final flow is the slaby pahoehoe. It looks like it runs on the A'a flow, probably there is a reactivation of a tube or fissure that channels the pahoehoe flow due to a new injection of magma. There are 2 structures that were part of the complex of solar panels. Made of concrete, with a flat roof also made of concrete, they were not destroyed by the A'a flow but completely surrounded and almost buried. The roofs still preserve tephra with an accumulation of 50-60 cm. Metal and plastic structures are melted.

18/05/2022:

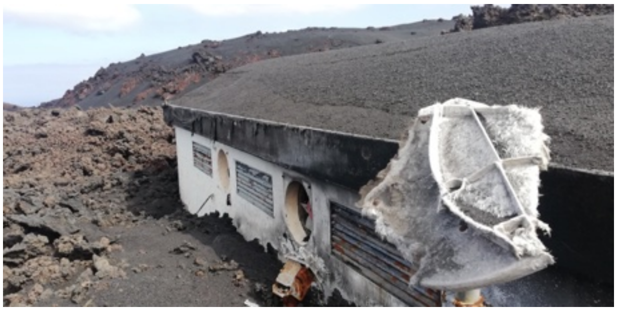
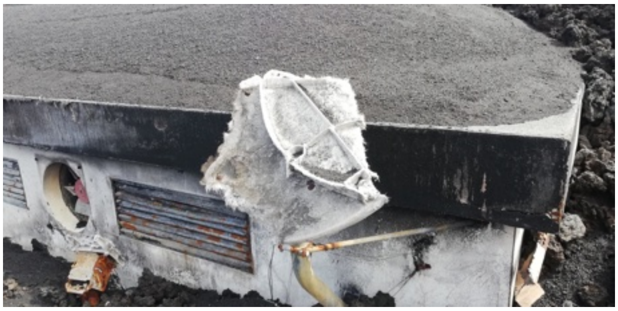
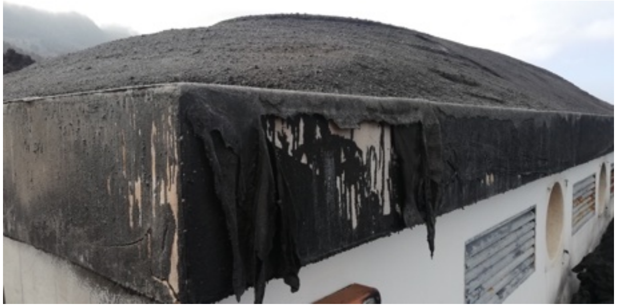
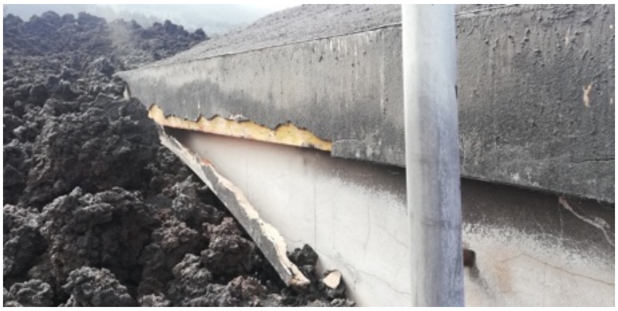
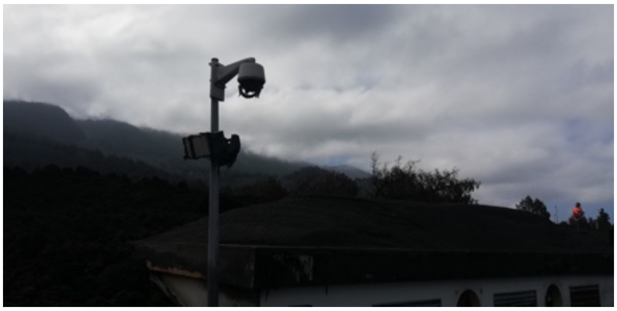
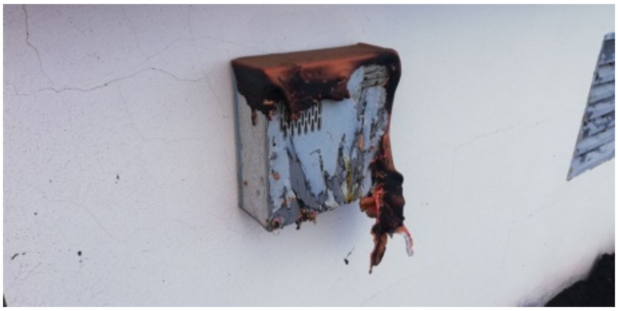
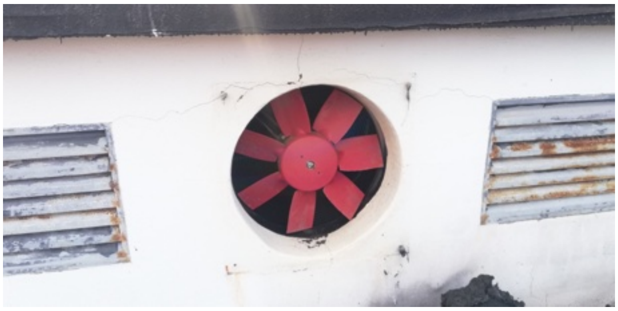
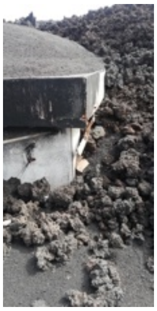
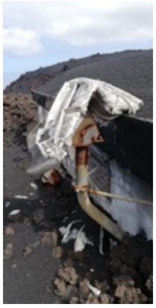

Name: DSLP-9
Location: 28.608921, -17.883020

Damage State: 5
Date: 18-05-2022

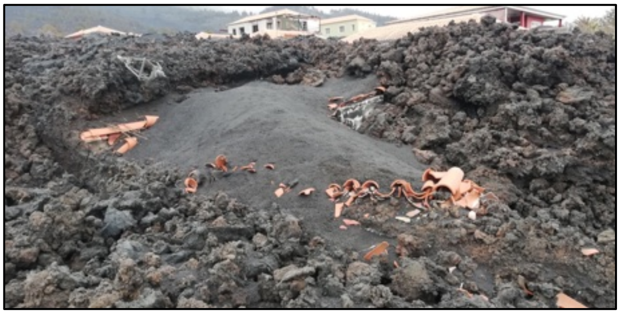

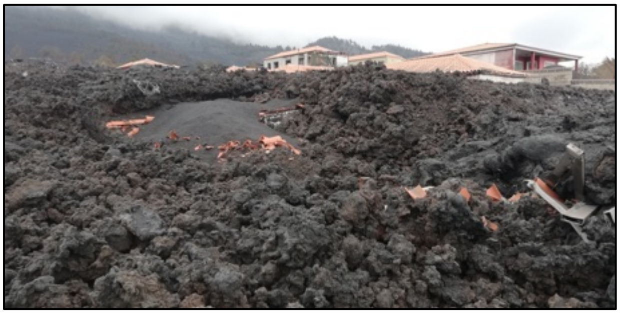

Footprint of the property:

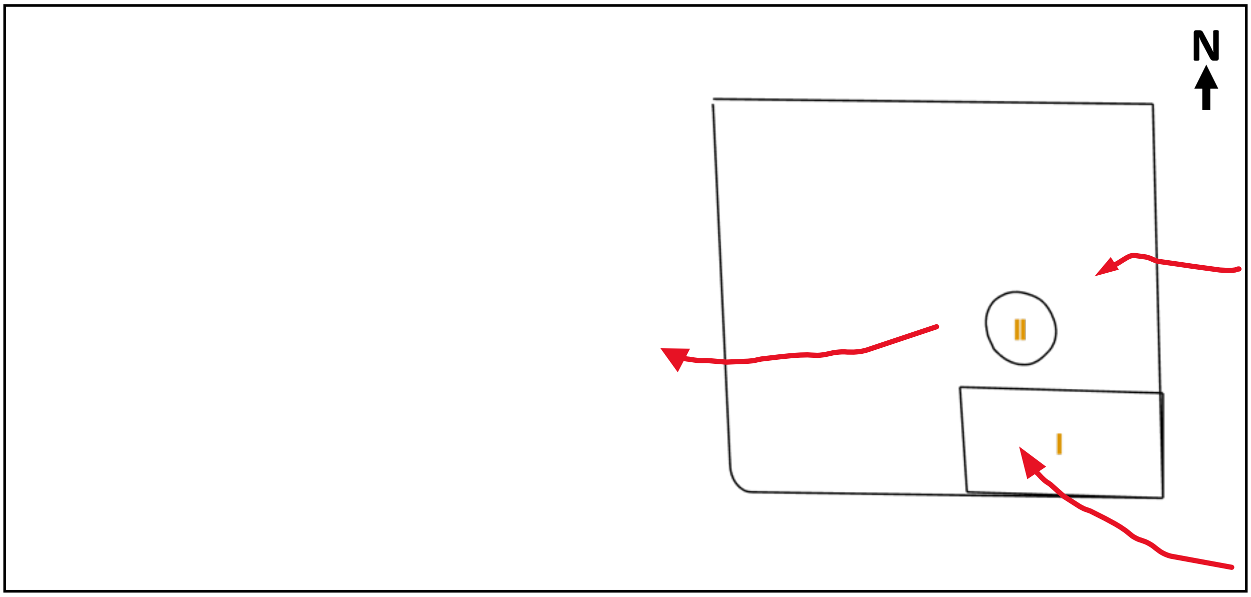

Type of structures in the property: I) Secondary 1, II) Pool?

N° Storeys: I) 0, II) 1

Openings:
I) Main house/ First floor:

Construction materials: I) ST0R1

Age of construction: New

Lava type: A'a, Pahoehoe

Lava thickness: m

Temporal order:

Damage: It may be part of the DSLP-5 property. It is completely destroyed and covered by lava and tephra. It looks like a secondary structure with metal beams, concrete walls and tiled roof. A'a lava observed in the NE a S sides. Pahoehoe observed to the W side.

Name: DSLP-10   Damage State: 5
Location: 28.608798, -17.883019   Date: 18-05-2022

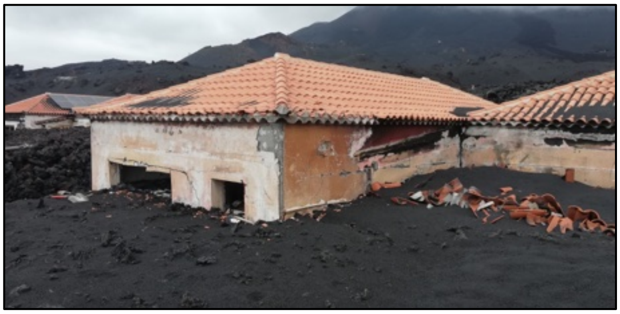

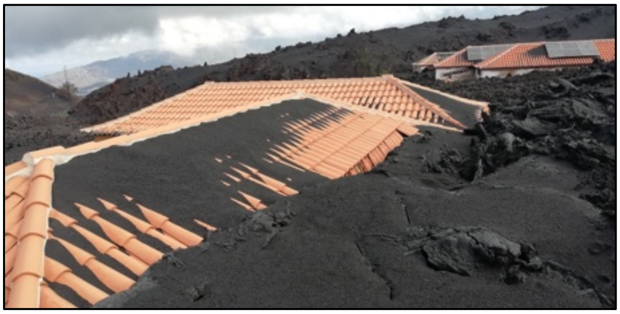

Footprint of the property:

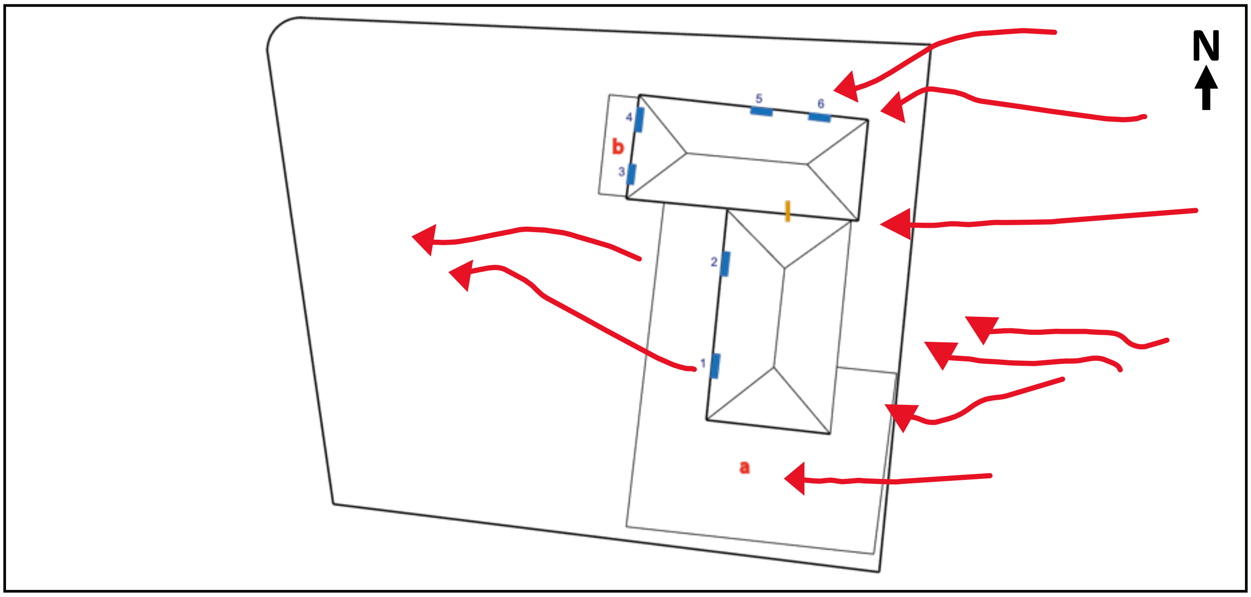

Type of structures in the property: I) Primary (main house) with accessory roof (a, b)

N° Storeys: I) 1

Openings:
I) Main house/ First floor: - 1: 228 cm wide with 35 cm from its top to the top of the pahoehoe lava,
- 2: 152 cm wide with 28 cm from its top to the top of the pahoehoe lava,
- 3: 65 cm wide with 48 cm from its top to the top of the pahoehoe lava,
- 4: 194 cm wide with 45 cm from its top to the top of the pahoehoe lava,.
- 5: 114 cm wide with 55 cm from its top to the top of the A'a lava,
- 6: 114 cm with 55 cm from its top to the top of the pahoehoe lava,

Construction materials: I) AT0R1

Age of construction: New

Lava type: A'a, Pahoehoe

Lava thickness: ~ 2 m

Temporal order:

Damage: There is structural damage all over the walls. Accessory roofs (a, b) collapsed. Lava A'a can be seen on the N and E (covering up to 1/3 of the house) and pahoehoe on the E (2/3) and S side. There are 140 cm from the axis of the double pitch of the roof until where the pahoehoe lava covered the roof. Lava A'a was channeled down by the south side of the house.

18/05/2022:

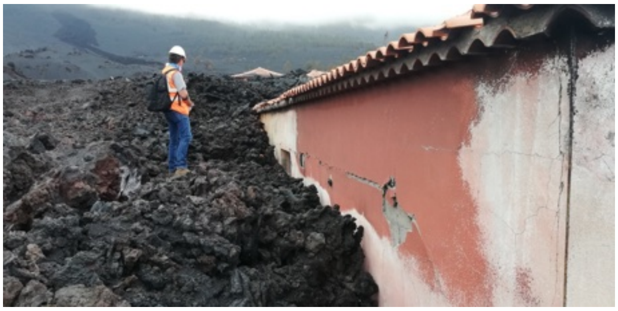

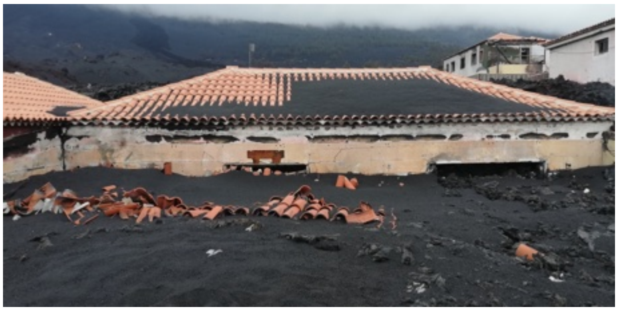

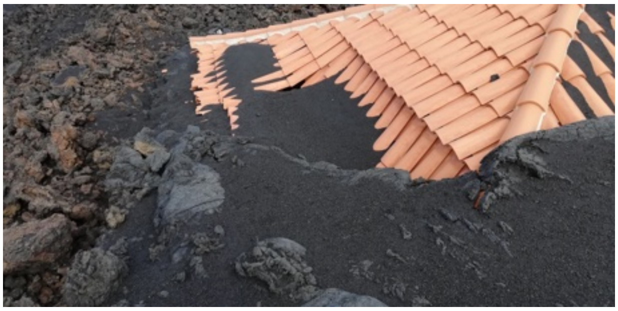

Name: DSLP-11
Location: 28.608698, -17.88273

Damage State: 5
Date: 19-05-2022

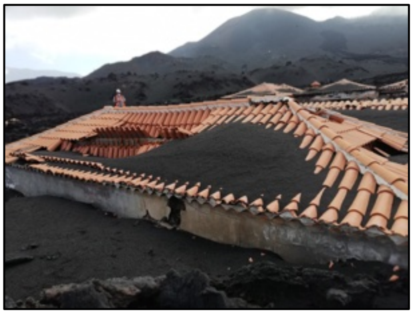

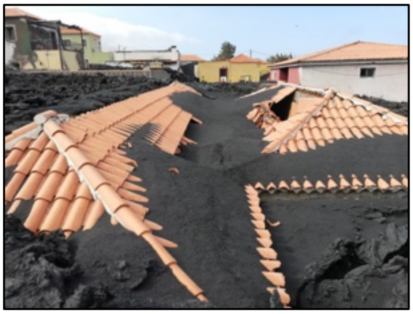

Footprint of the property:

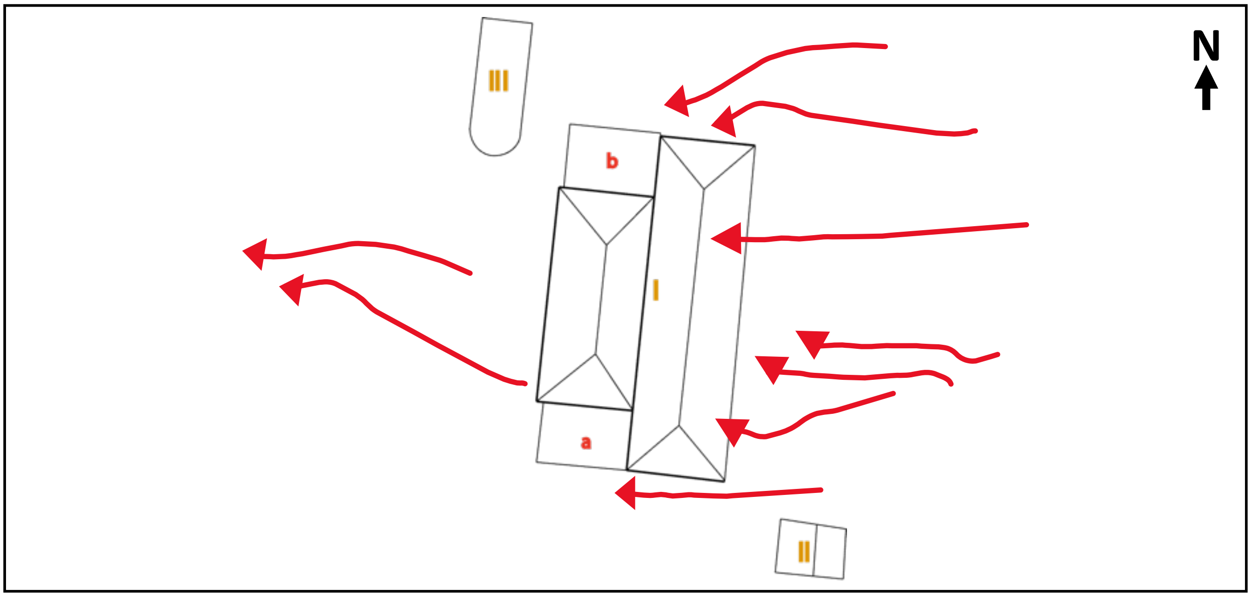

Type of structures in the property: I) Primary (main house) , II) Secondary (?), III) Pool

N° Storeys: I) 1, II) 1, III) 0

Openings:
I) Main house/ First floor:

Construction materials: I) AT0R1

Age of construction: New

Lava type: Pahoehoe

Lava thickness: ~ 2 m

Temporal order:

Damage: Totally surrounded by pahoehoe lava. Tephra accumulated in the middle of the 2 roof pitches generates collapse. There are cracks in the walls (facade). There is structural damage but not major, it is possible, but we are not 100% sure (it cannot be seen due to the lava flood).

26/10/2021:

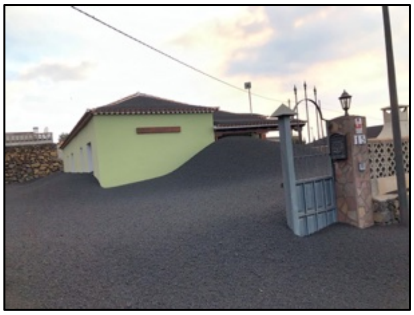

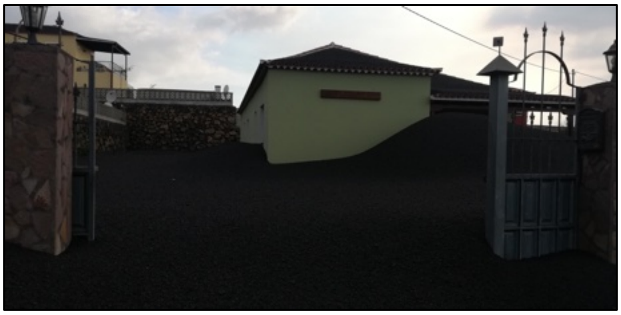

19/05/2022:

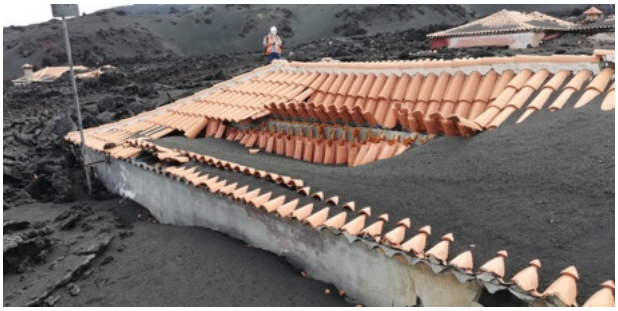

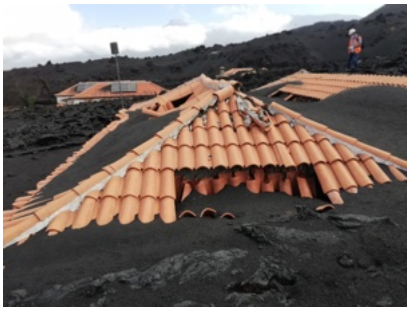

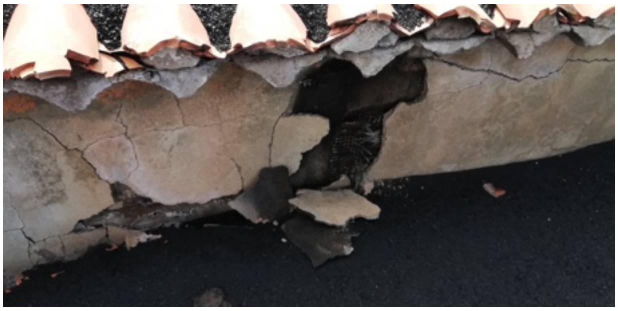

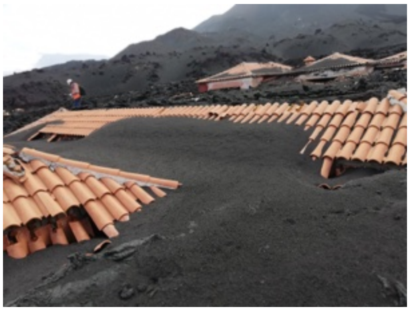

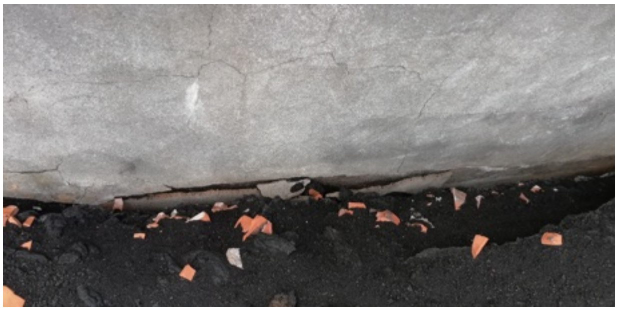

Name: DSLP-12
Location: 28.608502, -17.882884

Damage State:
Date: 19-05-2022

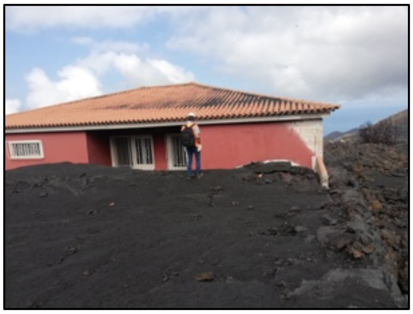

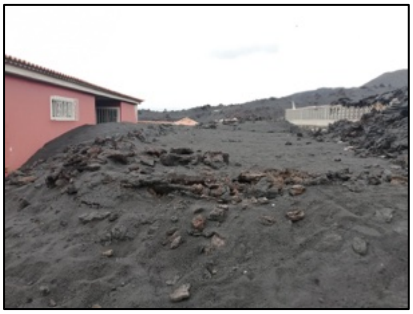

Footprint of the property:

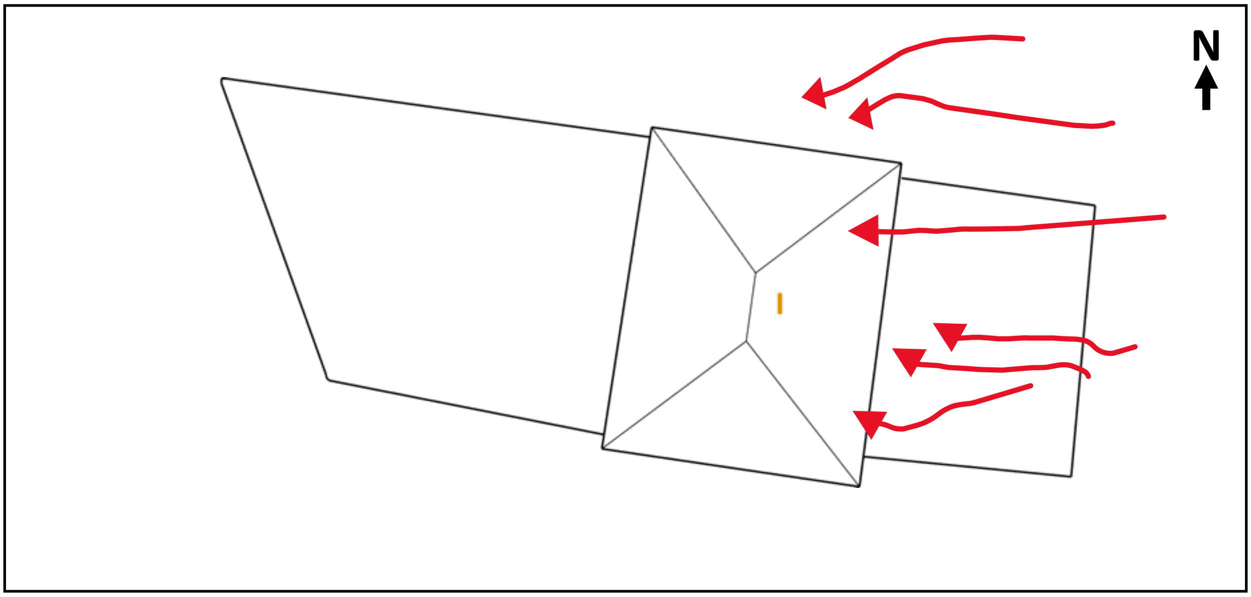

Type of structures in the property: I) Primary (main house)

N° Storeys: I) 1

Openings:
I) Main house/ First floor:

*between houses 12 and 10 there is a part of an accessory roof washed away by lava A'a.

Construction materials: I) AT0R1

Age of construction: New

Lava type: Pahoehoe

Lava thickness: m

Temporal order:

Damage: House surrounded by pahoehoe lava coming from the E and touching the NE corner. The heat removes red paint leaving the wall white and what is left of the paint appears bubbly. From the bottom of the roof to the top of the pahoehoe lava there are 130 cm. Structural damage (cracks in the walls) is observed. There are deflation cracks on the edges of the pahoehoe lava. We entered the house and took photos, there are fractures in every room of the house. On the N wall of the house an iron rod sticks out of it. This could be very bad for the house as in this coastal environment, it can be corroded very quickly.

08/02/2022:

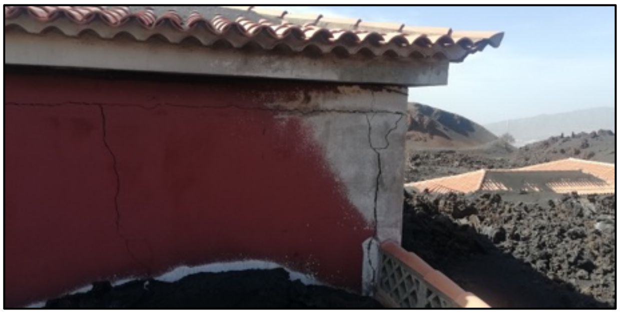

19/05/2022:

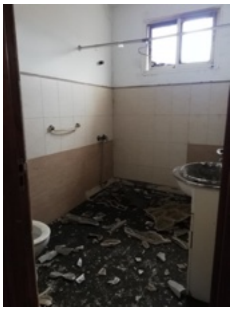
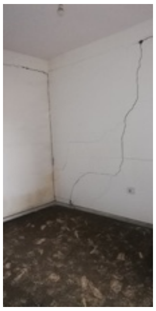
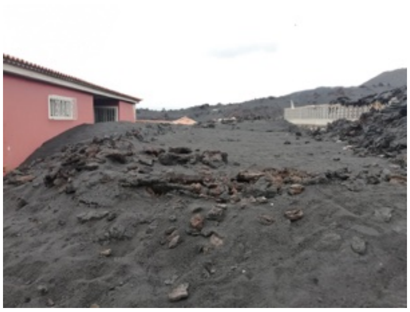
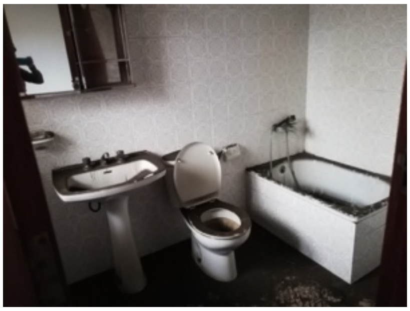
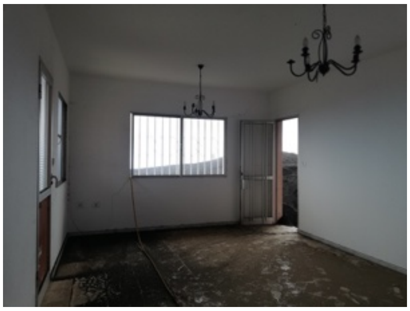
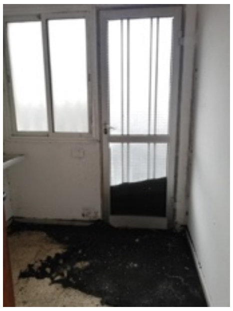
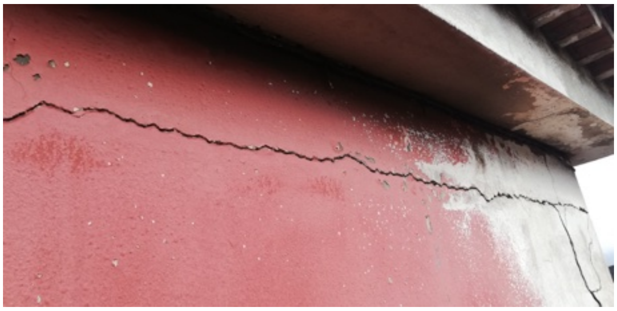
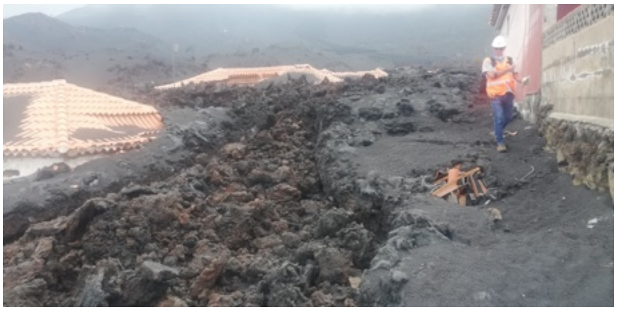
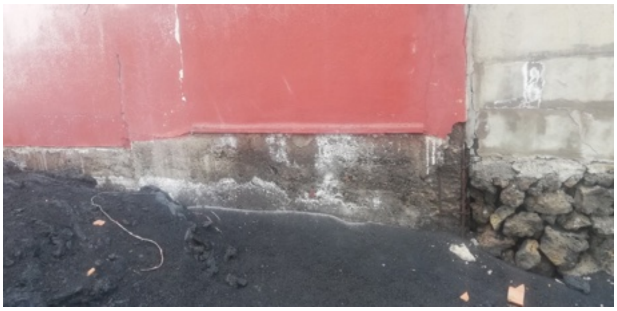

Name: DSLP-13
Location: 28.608547, -17.883503

Damage State:
Date: 19-05-2022

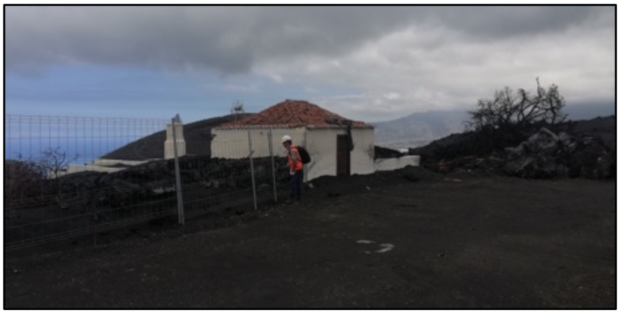

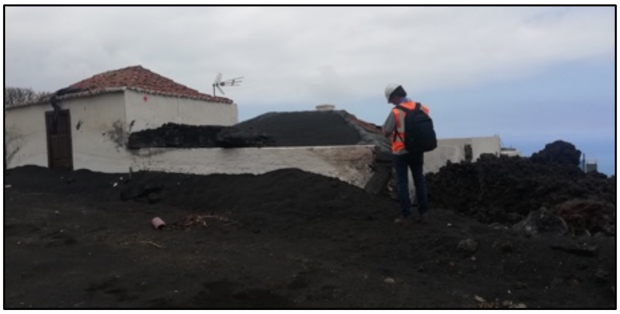

Footprint of the property:

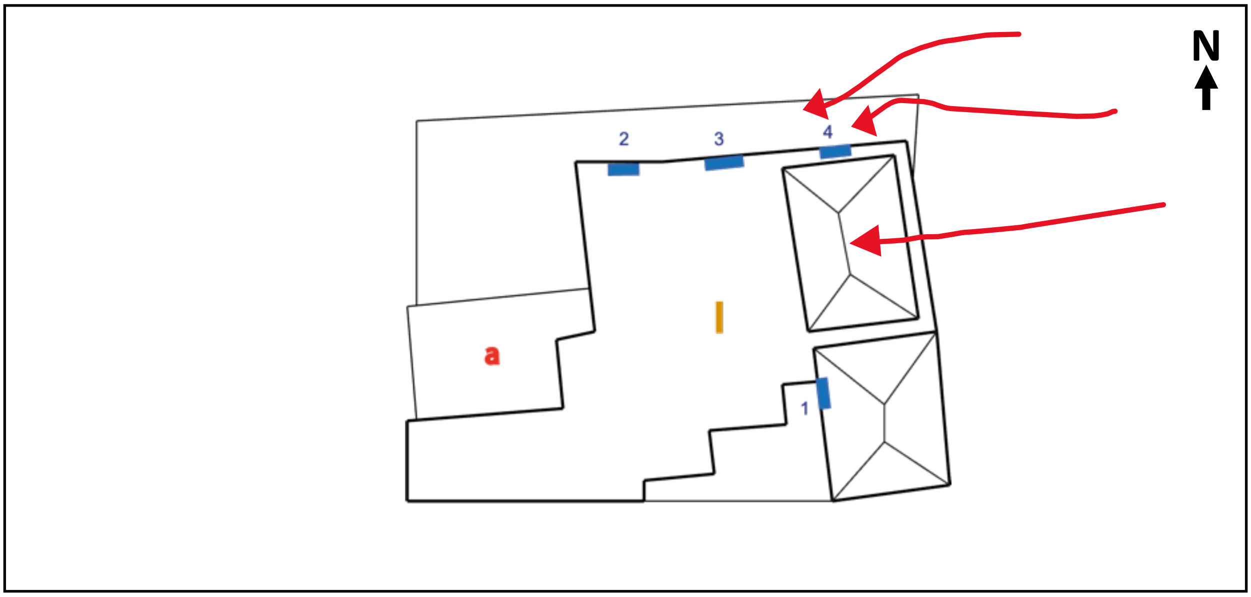

Type of structures in the property: I) Primary (main house) with accessory roof (a)

N° Storeys: I) 1

Openings:
I) Main house/ First floor: - 1: 82 cm wide (door), - 2: 94 cm wide (door), -3: 108 cm wide (window), - 4:78 cm wide (window)

*The lava from the road that covered the entrance of the house was removed.

Construction materials: I) AT2B1

Age of construction: Old

Lava type: Pahoehoe

Lava thickness: m

Temporal order:

Damage: Pahoehoe lava enters opening 4 but just a little and then surrounds the house to the N. Tephra accumulated to the north of the house protects openings 2 and 3 and blocks the advance of lava A'a into the house. There are fractures in the block and broken stone walls, but it is not possible to see if the roof broke under the pahoehoe lava.

30/10/2021:

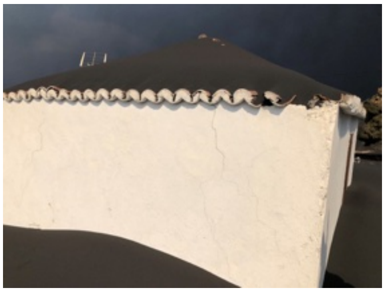

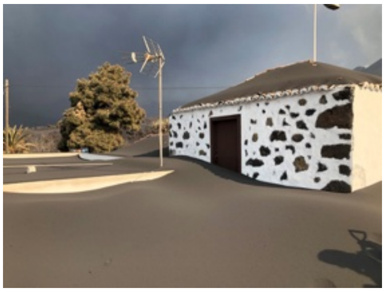

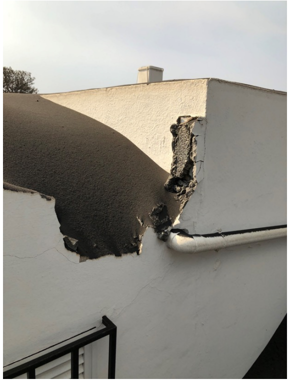

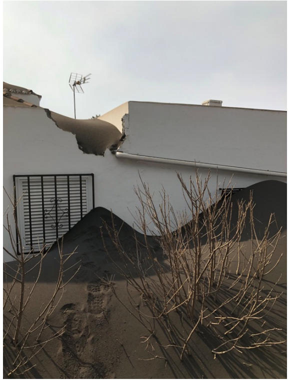

11/02/2022:

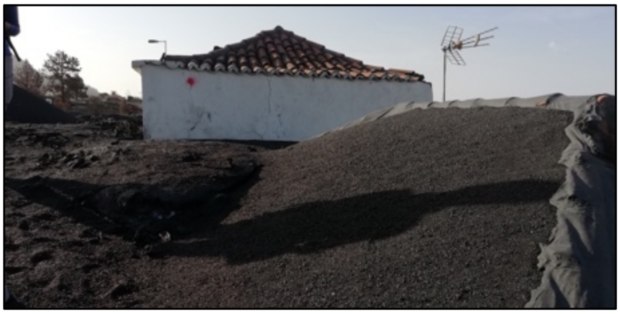

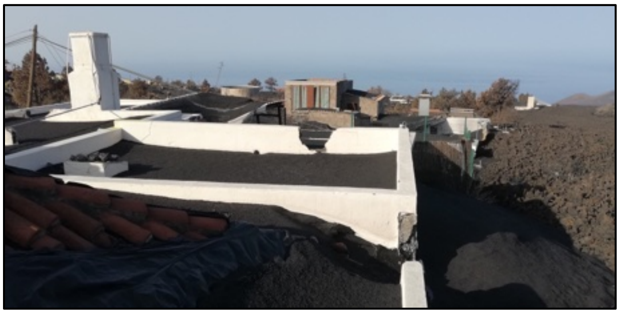

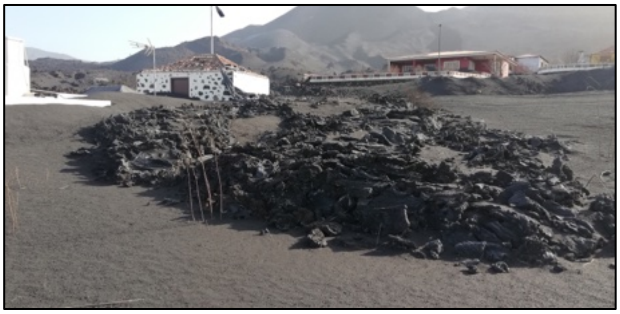

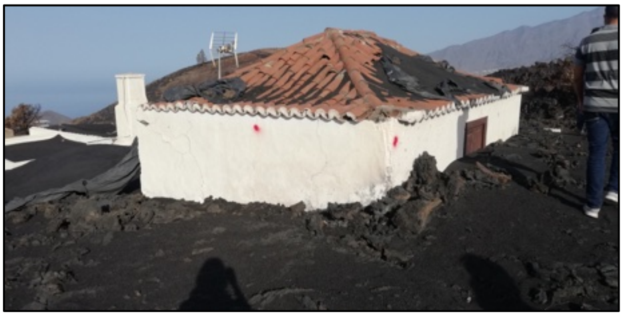

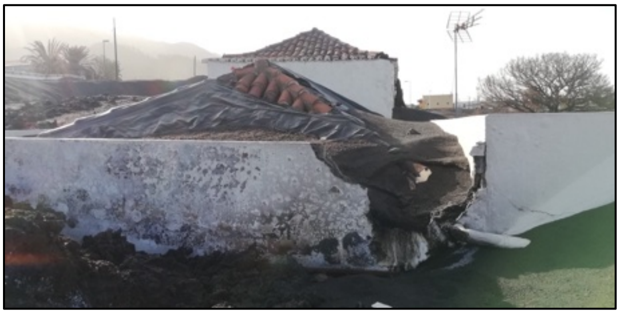

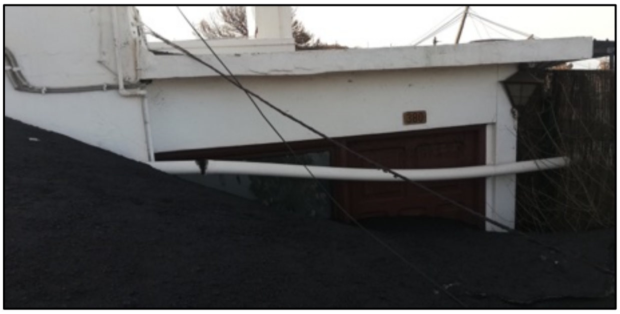

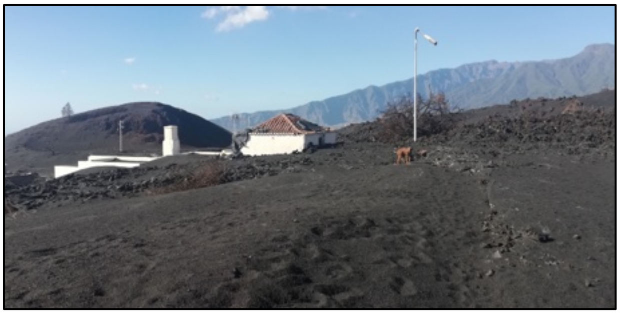

19/05/2022:

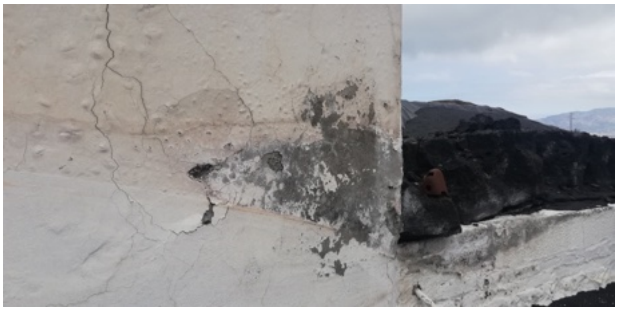

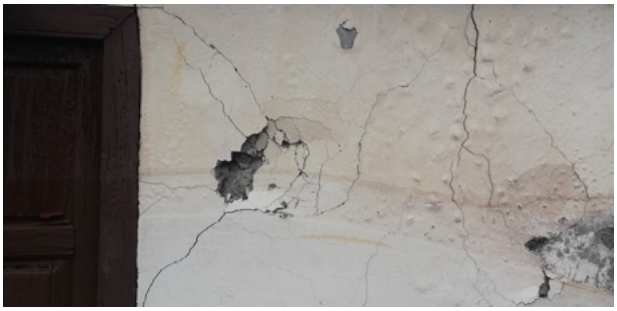

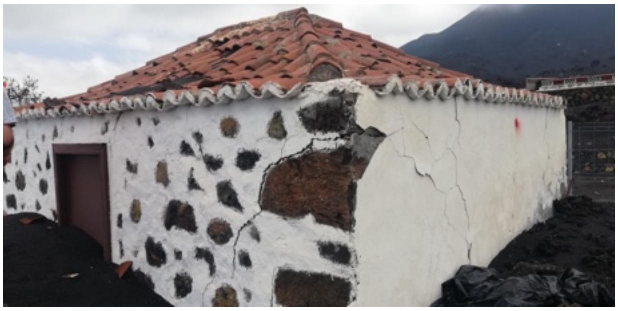

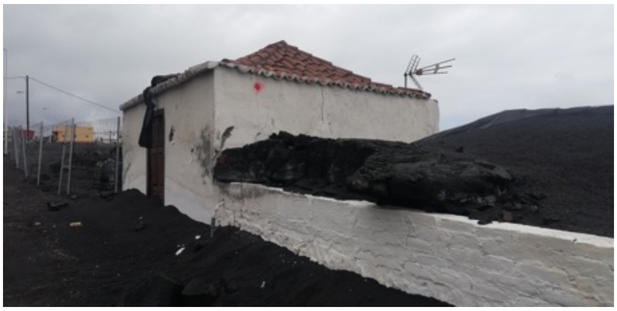

Name: DSLP-14
Location: 28.608648, -17.883728

Damage State:
Date: 19-05-2022

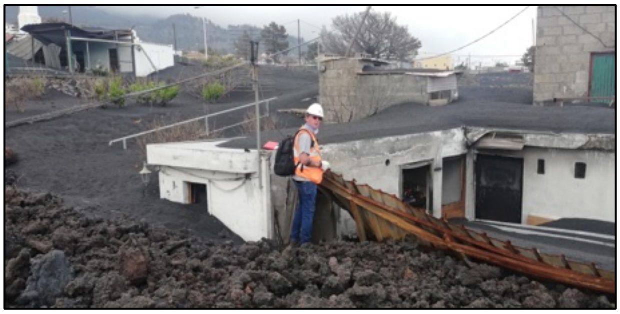
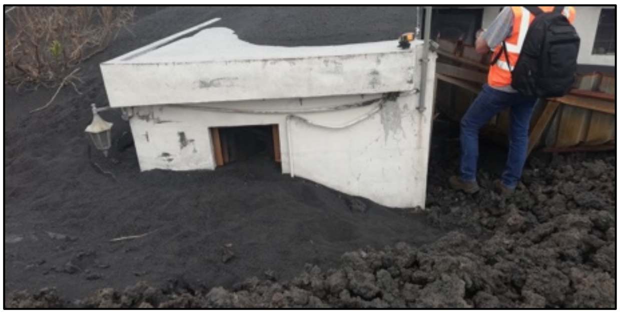

Footprint of the property:

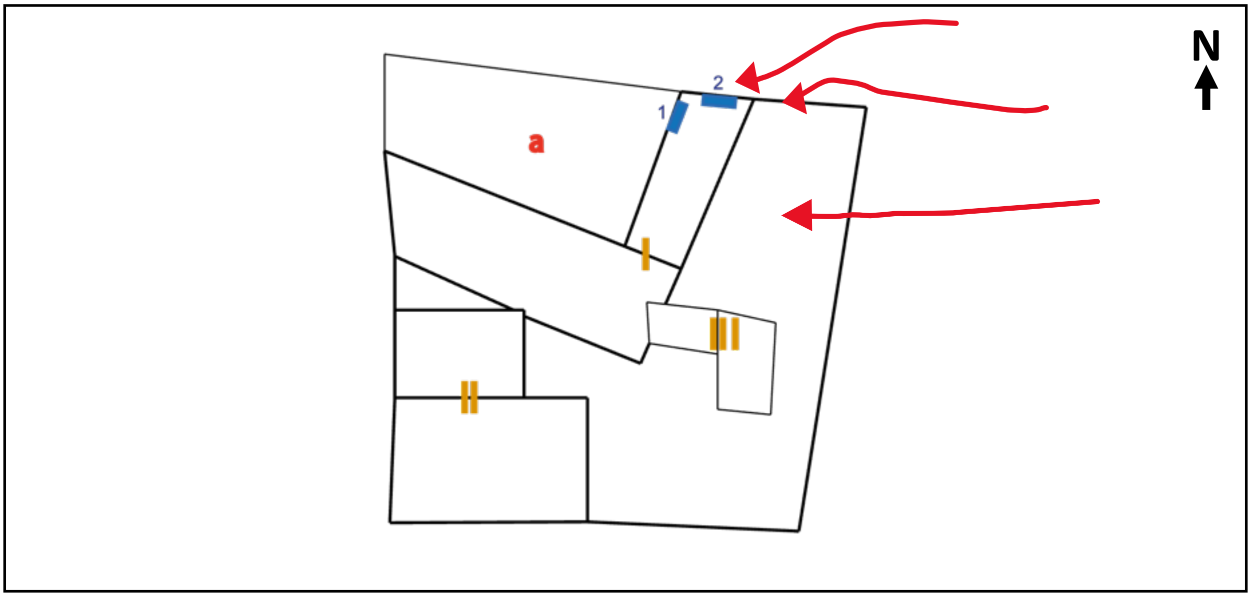

Type of structures in the property: I) Primary (main house), II) Secondary 1 (Storage?), III) Secondary 2 (Garage?)

N° Storeys: I) 1, II) 1, II) 1

Openings:
I) Main house/ First floor: - 1: 70 cm wide (door), - 2: 50 cm wide (window)

Construction materials: I) FC0R1, II) SX2R1, III)FX0E0

Age of construction: New

Lava type: A'a, pahoehoe

Lava thickness: m

Temporal order: Pahoehoe lava below A'a

Damage: Lava A'a enters through opening 2 (N) towards what looks like a bathroom and through the side door (opening 1). Corrugated metal accessory roof collapsed by tephra prior to lava arrival.

30/10/2021:

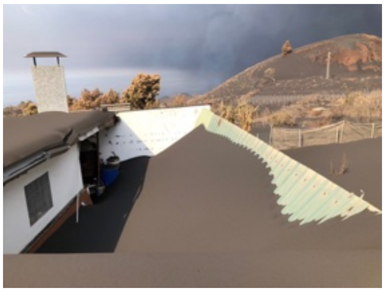
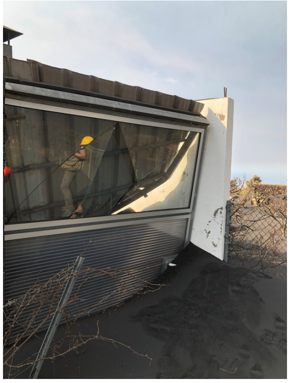
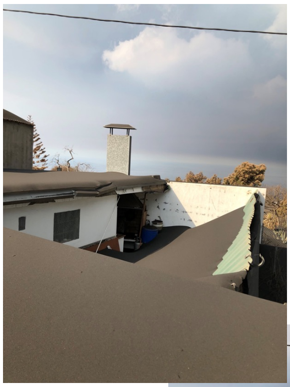
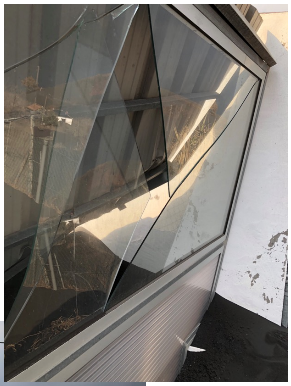
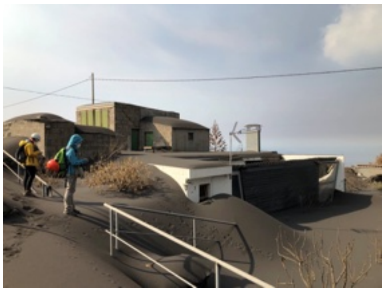
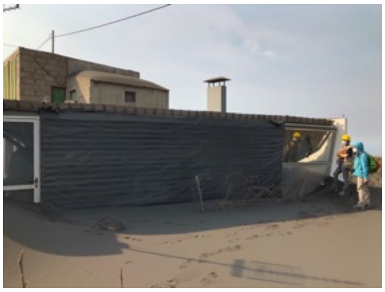
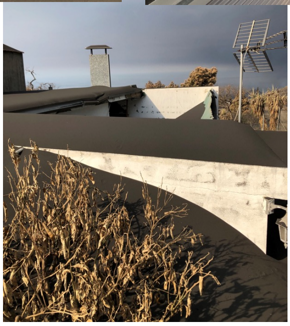

11/02/2022:

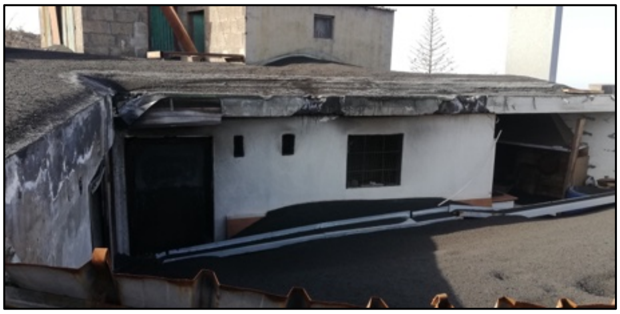
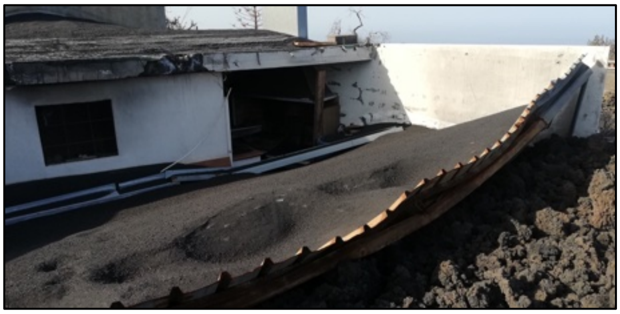
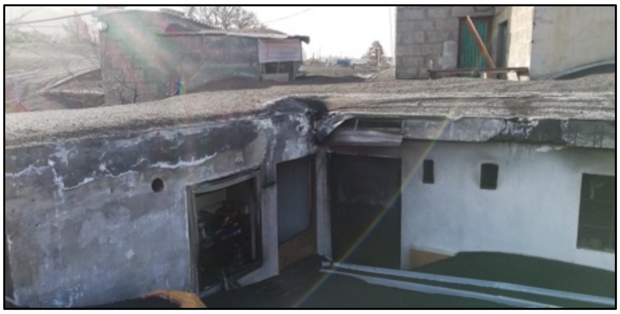
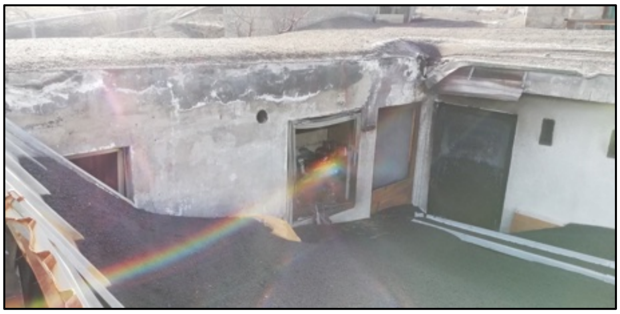
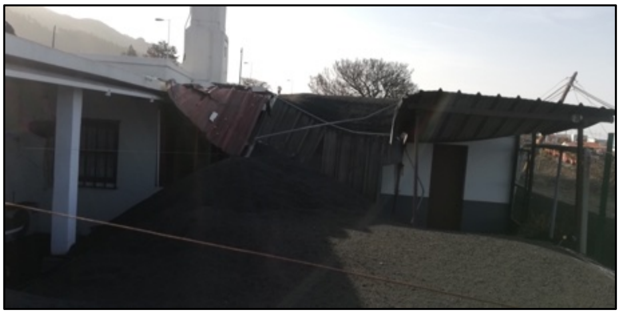

19/05/2022:

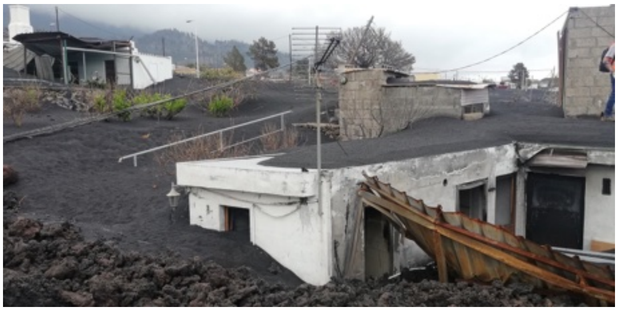

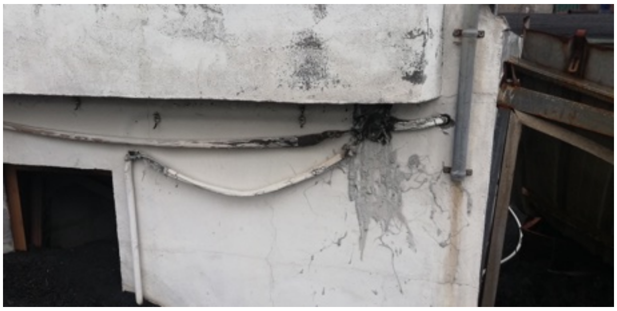

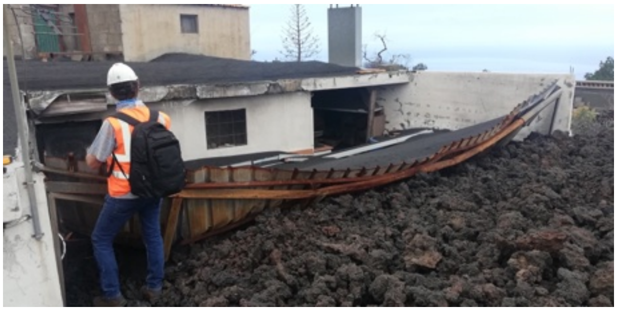

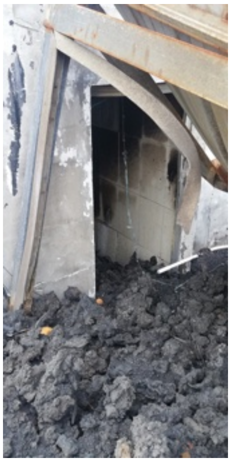

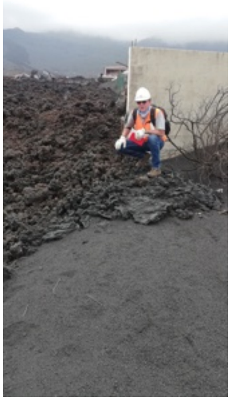

Name: DSLP-15 Damage State: 0
Location: 28.608684, -17.884058 Date: 19-05-2022

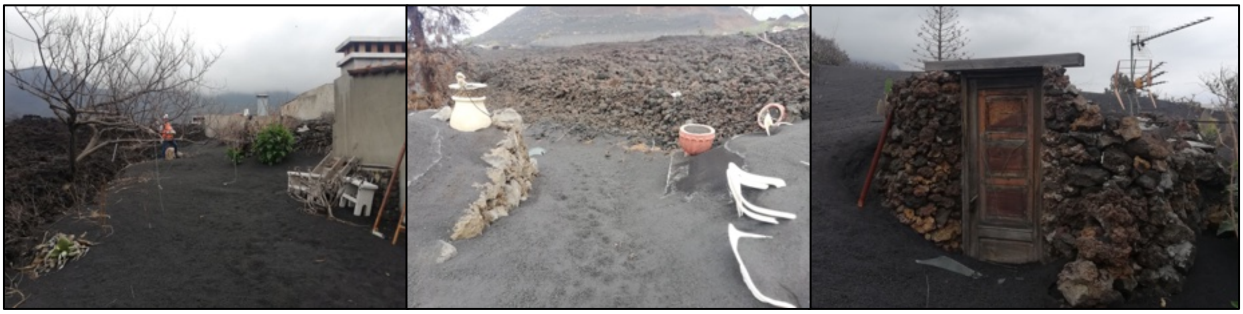

Footprint of the property:

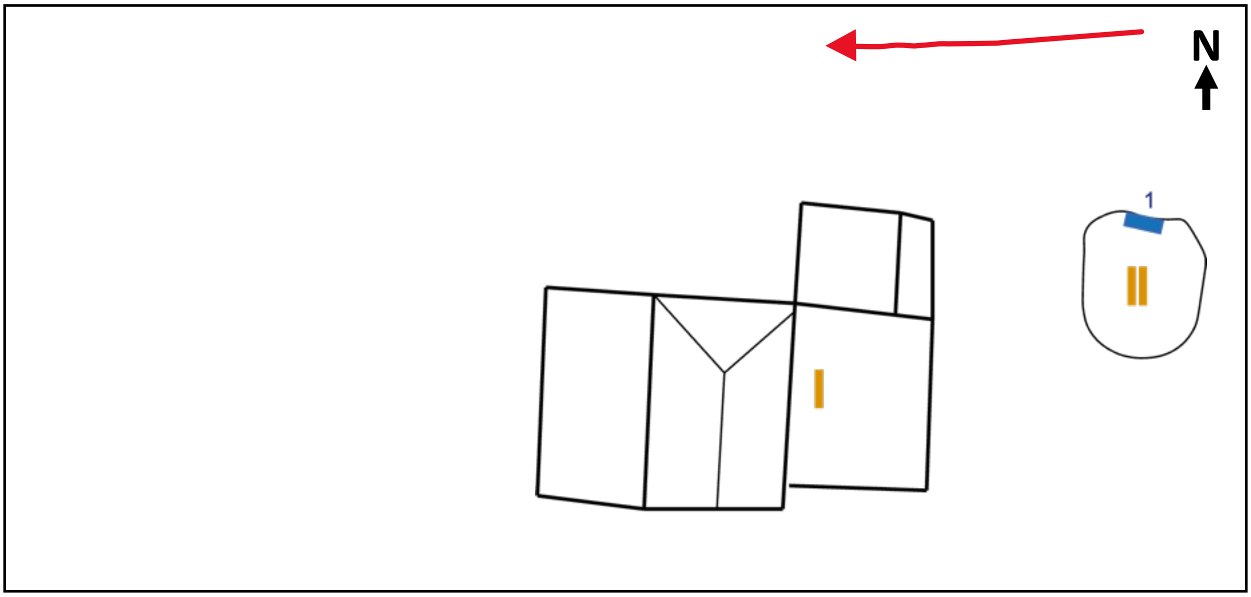

Type of structures in the property: I) Primary (main house), II) Secondary

N° Storeys: I) 1, II) 1

Openings:
I) Main house/ First floor:

Construction materials: I) ST0B1, II) FX2I1

Age of construction: New

Lava type: A'a

Lava thickness: m

Temporal order:

Damage: 460 cm of distance between the A'á lava and the structures of the house. The glass of the door facing the lava (opening 1, to the N) was probably broken by the heat but the lava did not touch the house and there are no other visible damages.

Name: DSLP-16 **Damage State: 5**

Location: 28.608745, -17.883628 **Date: 19-05-2022**

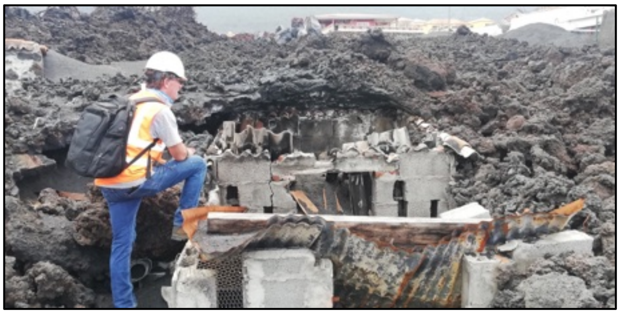

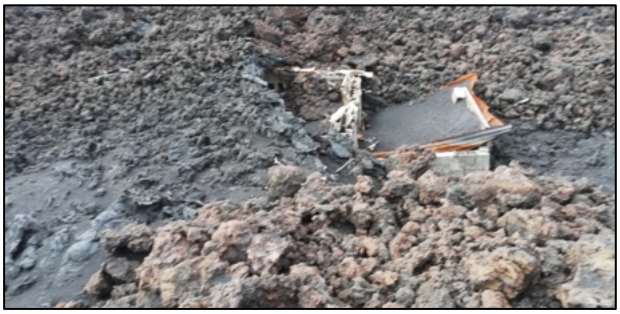

Footprint of the property:

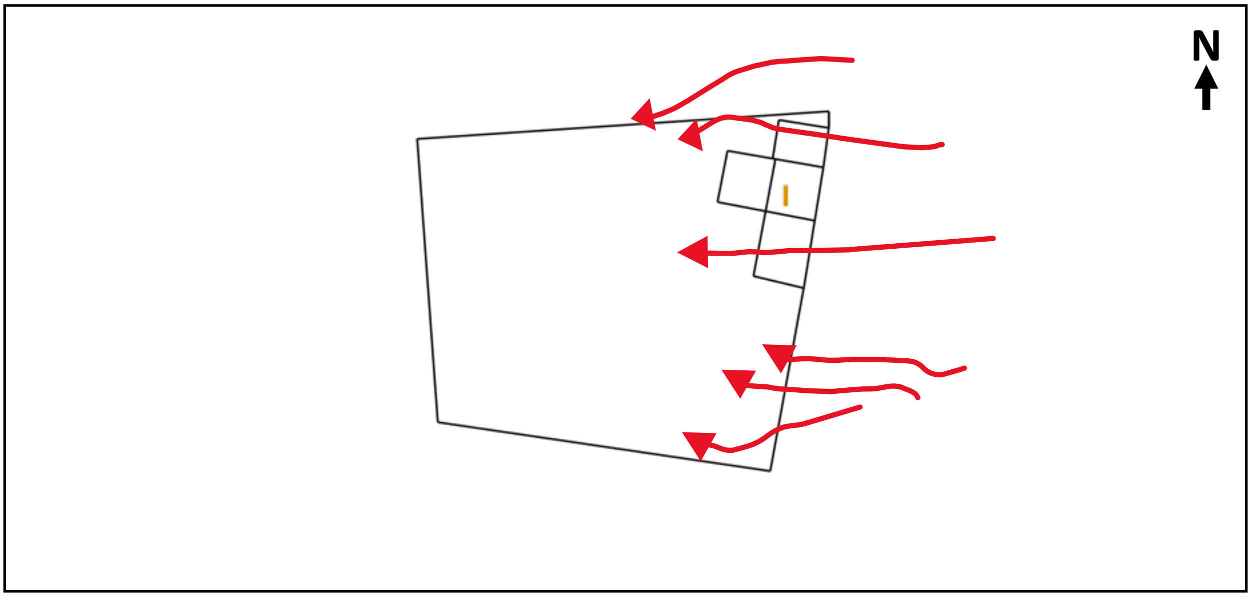

Type of structures in the property: I) Secondary? (Pajero?)

N° Storeys: I) 1

Openings:
I) Main house/ First floor:

Construction materials: I) FTCB1

Age of construction: New

Lava type: A'a, Pahoehoe

Lava thickness: ~ 2 m

Temporal order: A'a on top of Pahoehoe and viceversa

Damage: There are signs of 3 buried structures, one with broken stone and tiles, another with blocks and fiberglass and tiles roof , and another with blocks and a corrugated metal roof. The northern structure has A'a lava on top of pahoehoe. The other 2 structures are destroyed by pahoehoe lava and then lava A'a collapses the roofs.

Name: DSLP-17
Location: 28.609202, -17.883586

Damage State: 5
Date: 19-05-2022

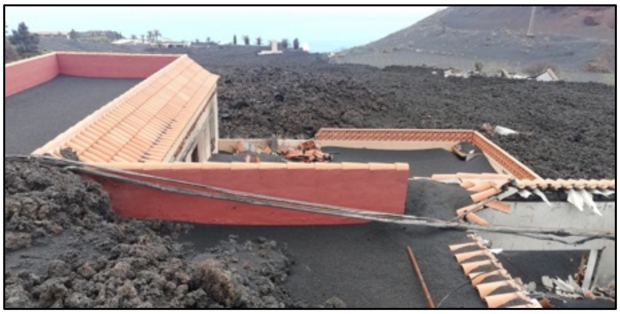

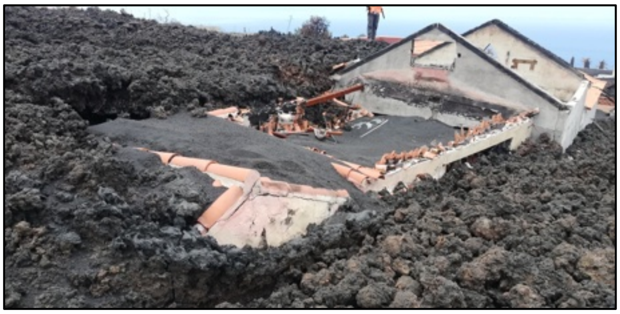

Footprint of the property:

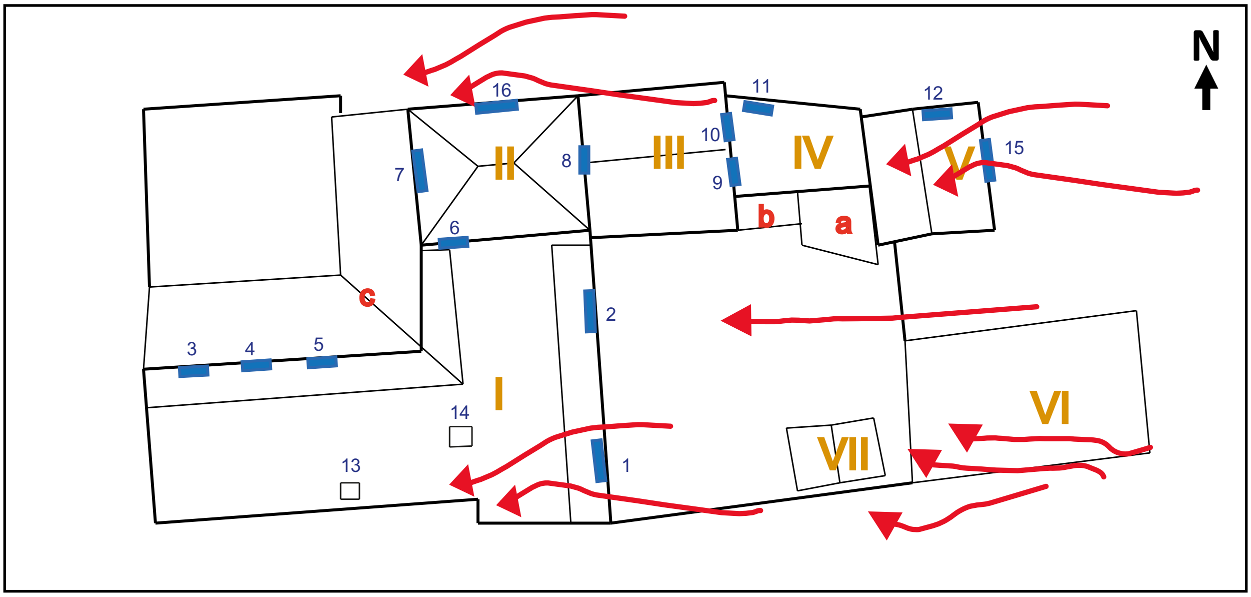

Type of structures in the property: I) Primary 1 with accessory roof (c), II) Primary 2; III) Primary 3, IV) Primary 4 with accessory roofs (a, b), V) Primary, VI) Secondary 1 (Garage), VII) Secondary 2 (storage)

N° Storeys: I) 1, II) 1; III) 1, IV) 1, V) 1, VI) 0, VII) 1

Openings:
I) Main house/ First floor: - 1: 115 cm wide, - 2: 180 cm wide, - 3: 120 cm wide, - 4: 98 cm wide,- 5: 120 cm wide,- 6: 97 cm wide (door), - 7: 178cm wide, - 8: , - 9: 100 cm wide, - 10: 100cm wide, - 11:, - 12: 198 cm wide,- 13: 60x60 cm,- 14: 60x60 cm, - 15: double window, - 16: 158 cm wide (window with bars)

*There is pahoehoe lava on the E and W side of the house (going down) and "transitional" on the S side. To the N there is only A’a.
*In structure (I) it can be seen that the lava entered through opening 6, then continued down the corridor, and also entered through the skylight in the ceiling (14).

Construction materials: I)FT1R1, II)AT0R1; III) AT0R1, IV) ST0R1, V) AT0R1, VI)SX0E0, VII)AX0E1

Age of construction: New

Lava type: A’a, Pahoehoe

Lava thickness: ~ 2 m

Temporal order: initial A’a lava, then thicker A'a (more fluid), then pahoehoe and finally A'a

Damage: All roofs collapsed except for Primary 1 (I). Lava A'a does not enter through openings 1 and 2, it only passes over the flat roof of (I). The bars protected the structure (II) from the lava entering through opening 16. Total failure of the S wall of (III) due to the external A’a flow. In (III) deflation is observed. A'a probably entered the house through opening E (15), filled the room up to the ceiling, and then breached wall W, draining the lava. There is evidence of lava on the walls (at least 2 m). A’a flow also entered the house through the opening 12. Composite material and tiles roofs supported by wooden beams (burned) are collapsed. Metal framing melted (windows). Wooden beams that supported the accessory roofs are burned.

30/10/2021:

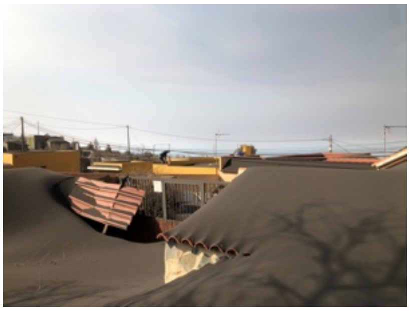

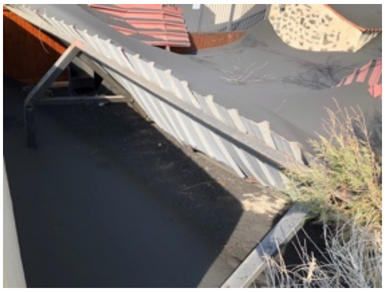

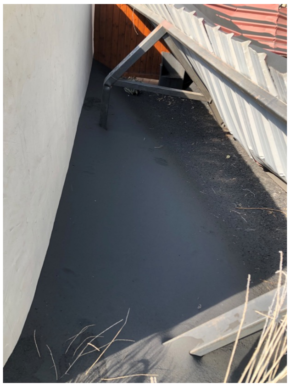

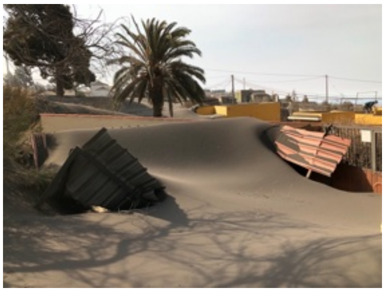

19/05/2022:

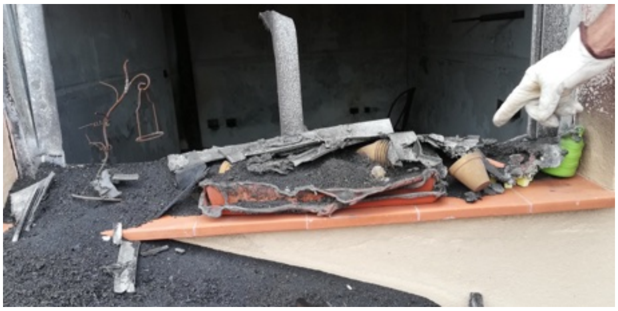

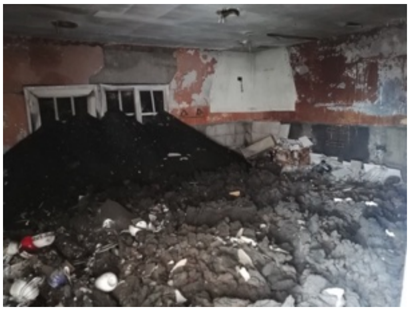

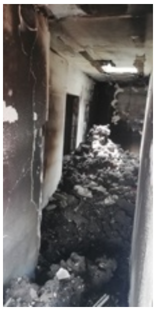

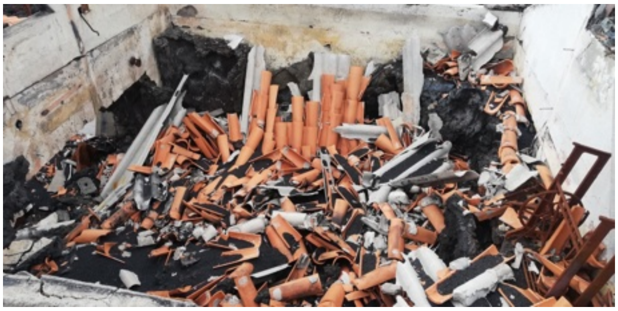

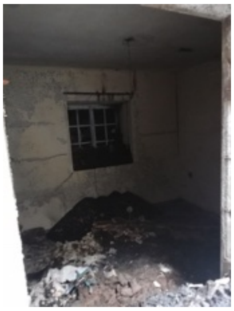

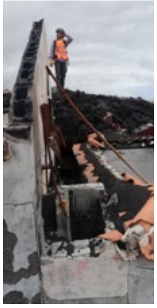

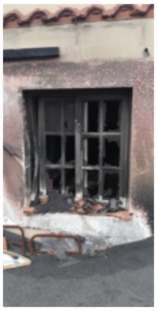

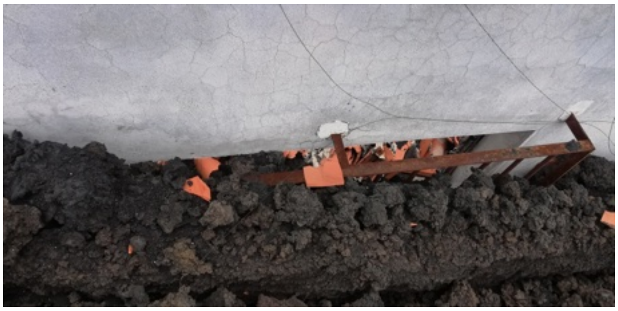

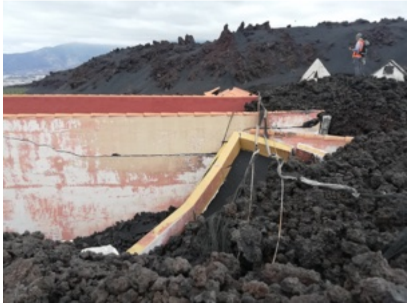

Name: DSLP-18
Location: 28.608551, -17.882365
Damage State: 3
Date: 17-05-2022

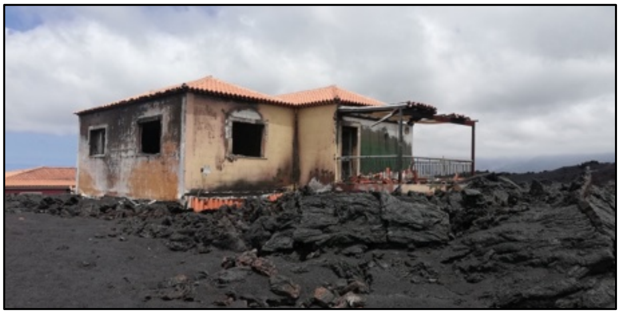

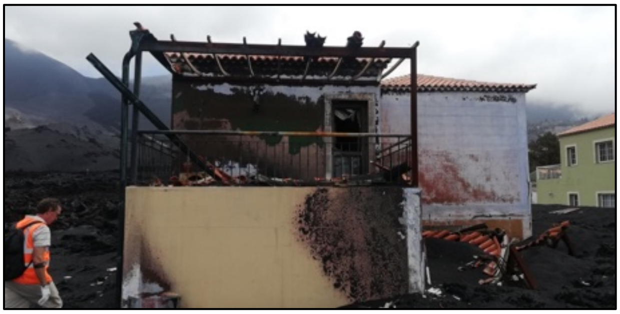

Footprint of the property:

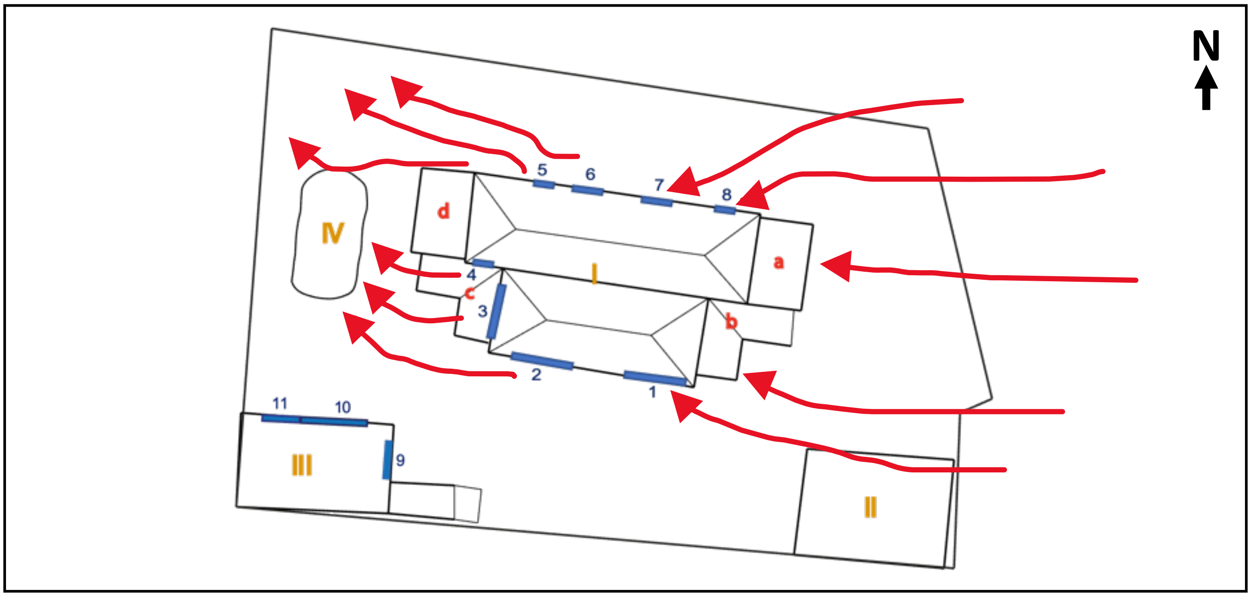

Type of structures in the property: I) Primary (main house) with accessory roofs (a,b,c,d), II) Secondary (Garage), III) Secondary (Barbecue), IV) Pool

N° Storeys: I) 2, II) 0, III) 1

Openings:
I) Main house/ First floor: - 1 & 2: 195 cm long with 10 cm from its top to the top of the lava, - 3: 167 cm long with 12 cm from its top to the top of the lava, - 4: 196 cm long with 40 cm from its top to the top of the lava, - 5: 75 cm long, - 6: 166 cm long with 36 cm from its top to the top of the lava, - 7: 196 cm long with 35 cm from its top to the top of the lava
I) Main house/ Second floor: - North: 3 single windows + 2 double windows, - West: Porch w/ 1 door, - South: 2 double windows, East: Porch + 1 door and 1 window
III) Barbecue: - 9: 137x110 cm, - 10: 253 cm long, - 11: 118cm long

Construction materials: I) AT0R2, II) ST0E0, III) ST0R1

Age of construction: New

Lava type: Pahoehoe

Lava thickness: 2.33 m

Temporal order:

Damage: Flow impacting from the E to the front of the main house. First floor completely flooded by lava entering openings E, NE and SE. Lava comes out through openings of the W, NW and SW of the house. No major structural damage but fire damage in the interior of the house. Thermal sagging in the steel rafters of the accessory roofs (Tube support 8x8 cm and 4x6 cm) and roof of composite material and tiles collapsed. Thermal sagging in the big steel rafter, burnt wood beams and collapsed fiberglass and tile roof of the Barbecue structure. Pool (IV) completely covered by lava.

28/10/2021:

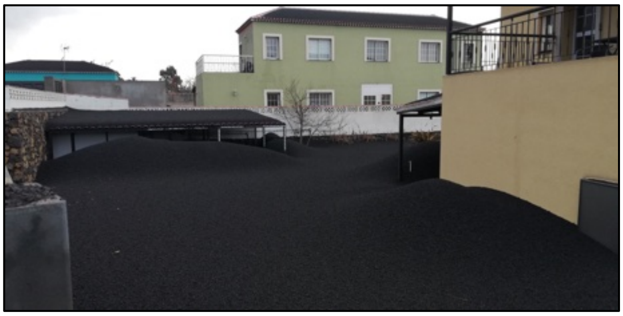
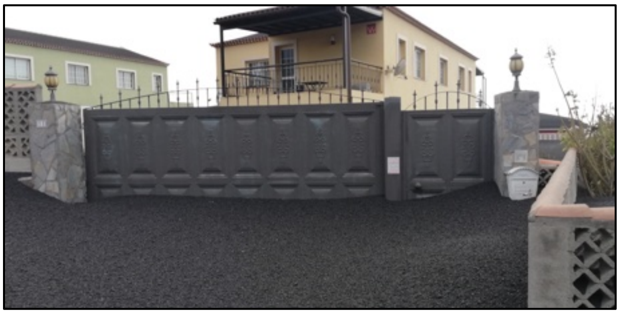
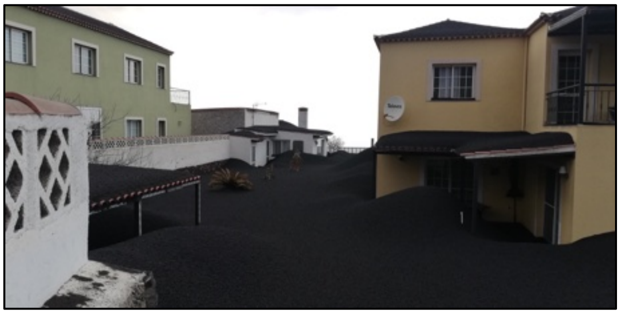
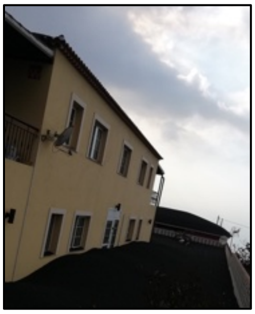

08/02/2022:

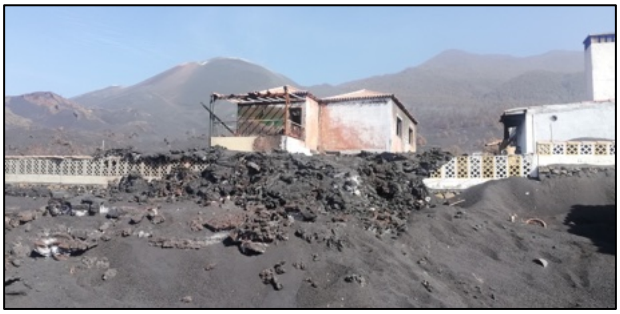
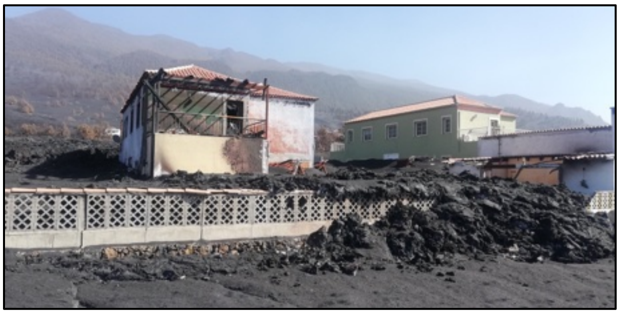
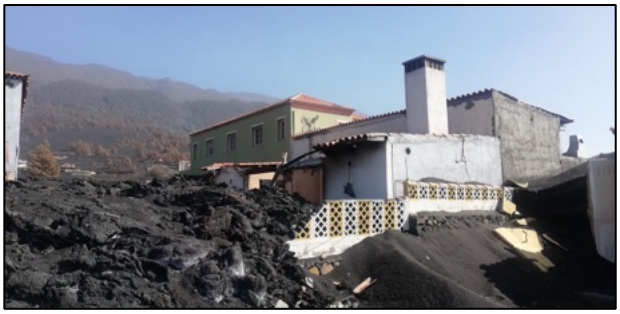

17/05/2022:

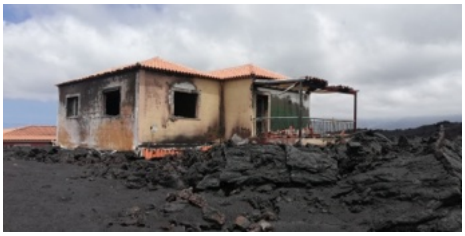
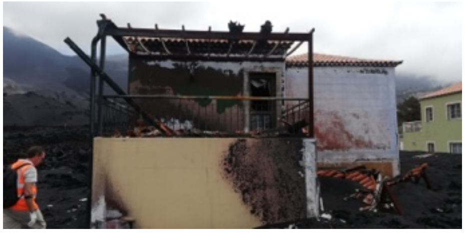

17/05/2022:

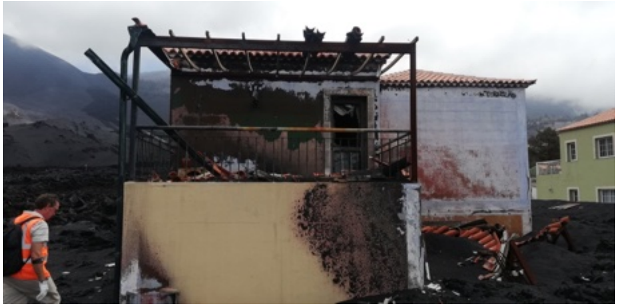

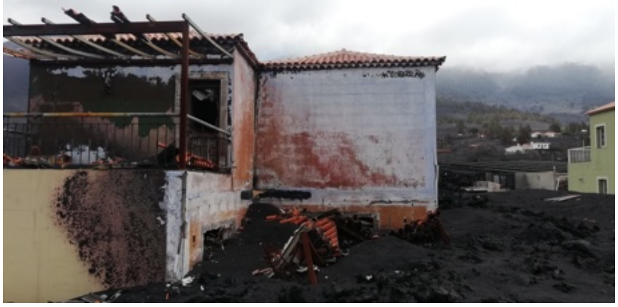

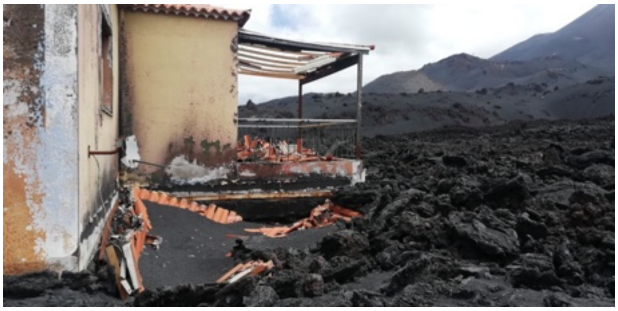

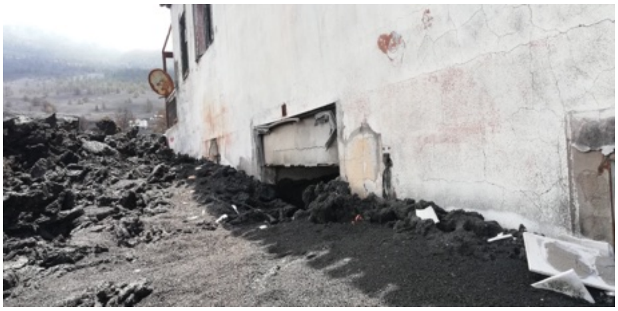

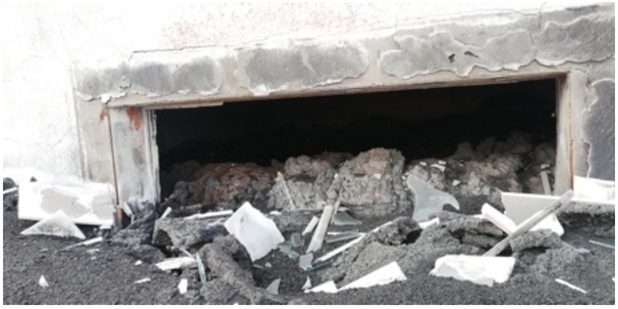

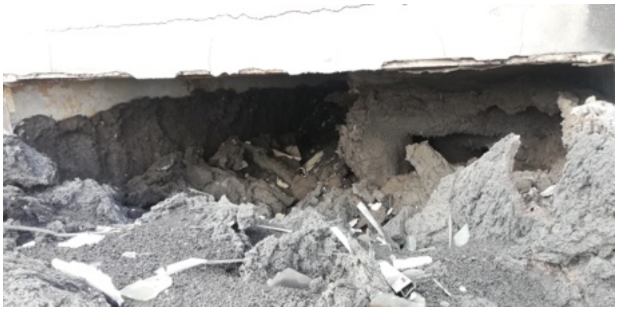

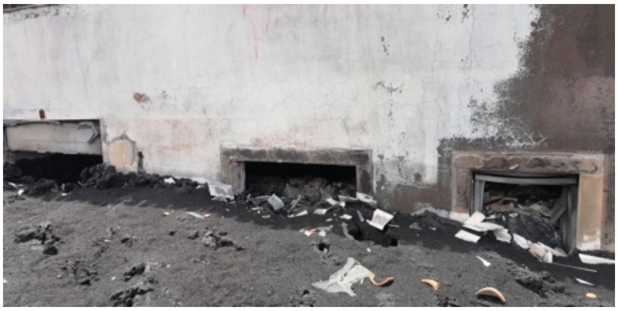

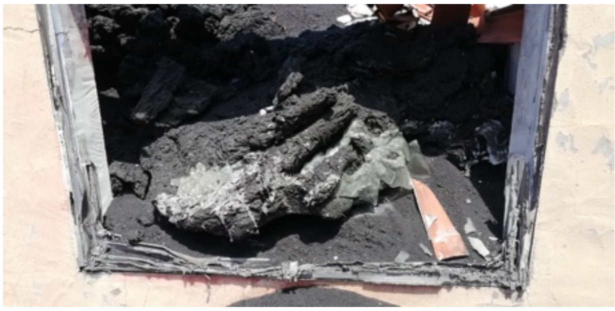

Name: DSLP-19 **Damage State: 5**
Location: 28.60931, -17.883784 **Date: 20-05-2022**

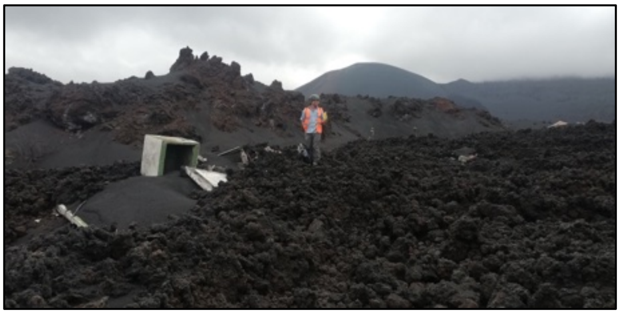

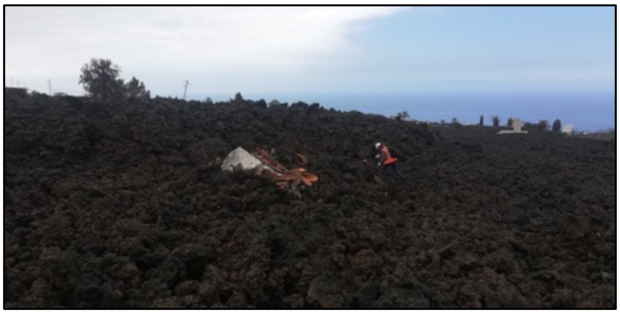

Footprint of the property:

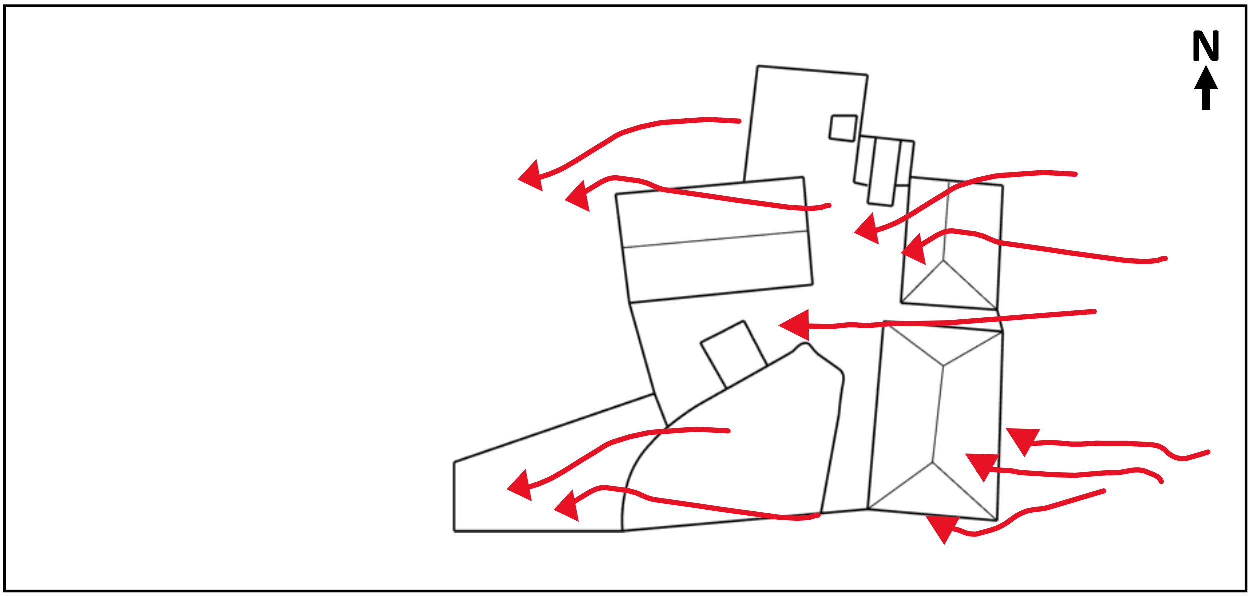

Type of structures in the property:

N° Storeys:

Openings:
I) Main house/ First floor:

Construction materials:

Age of construction:

Lava type: A'a, pahoehoe

Lava thickness: m

Temporal order:

Damage: Completely buried or washed away by A'a lava flow. Pahoehoe lava also appears in contact with one of the walls that is possible to see through the rubble with a new texture that we had not seen before. 10 m downstream there is another piece of the wall and tiles running SW and NW.

30/10/2021:

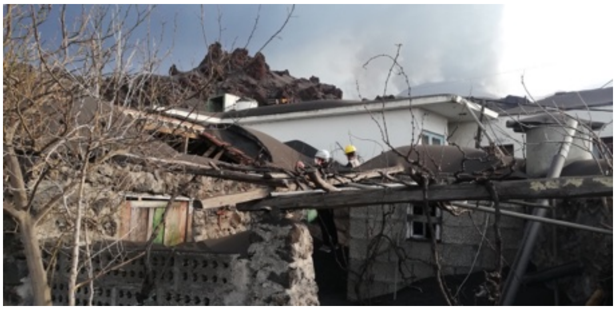

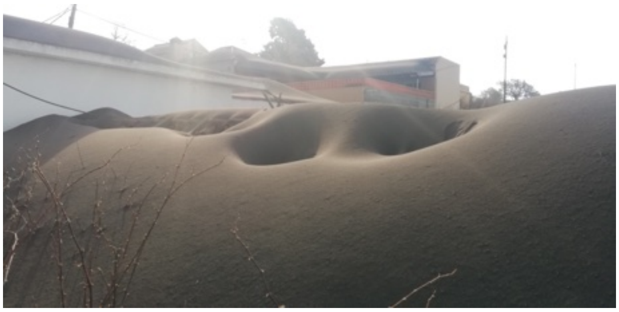

20/05/2022:

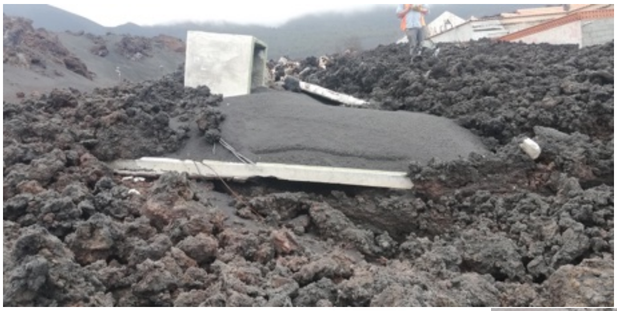

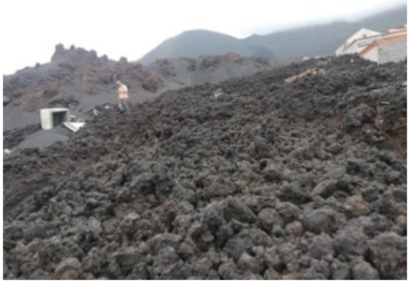

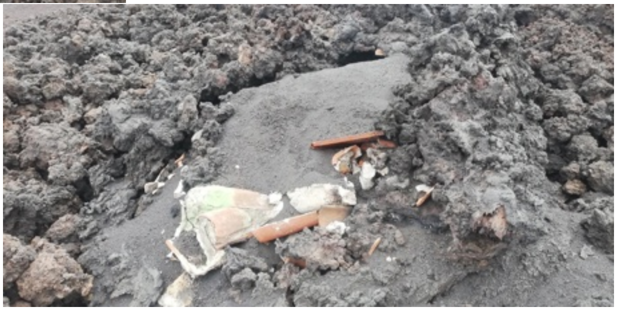

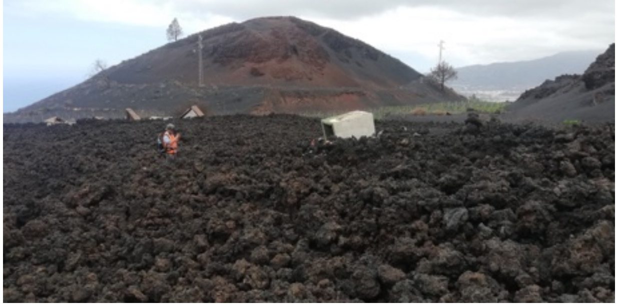

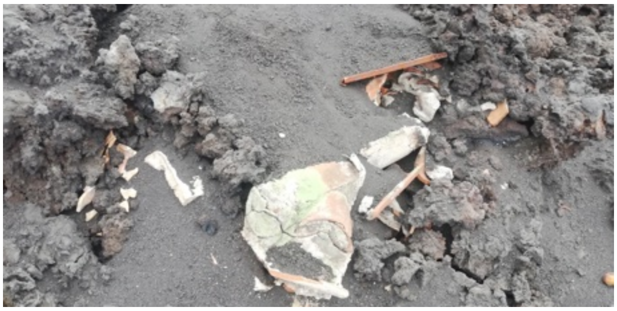

Name: DSLP-20
Location: 28.609445, -17.884313

Damage State:
Date: 20-05-2022

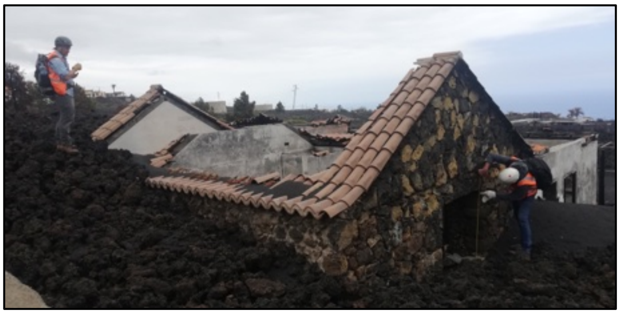

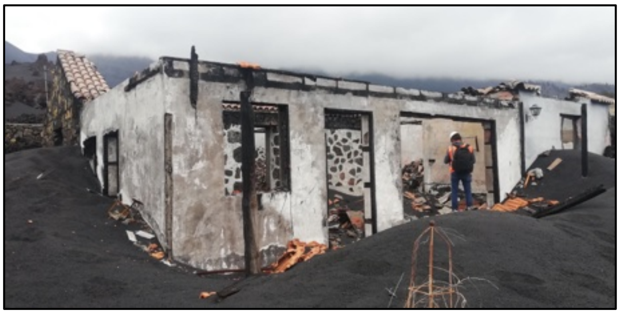

Footprint of the property:

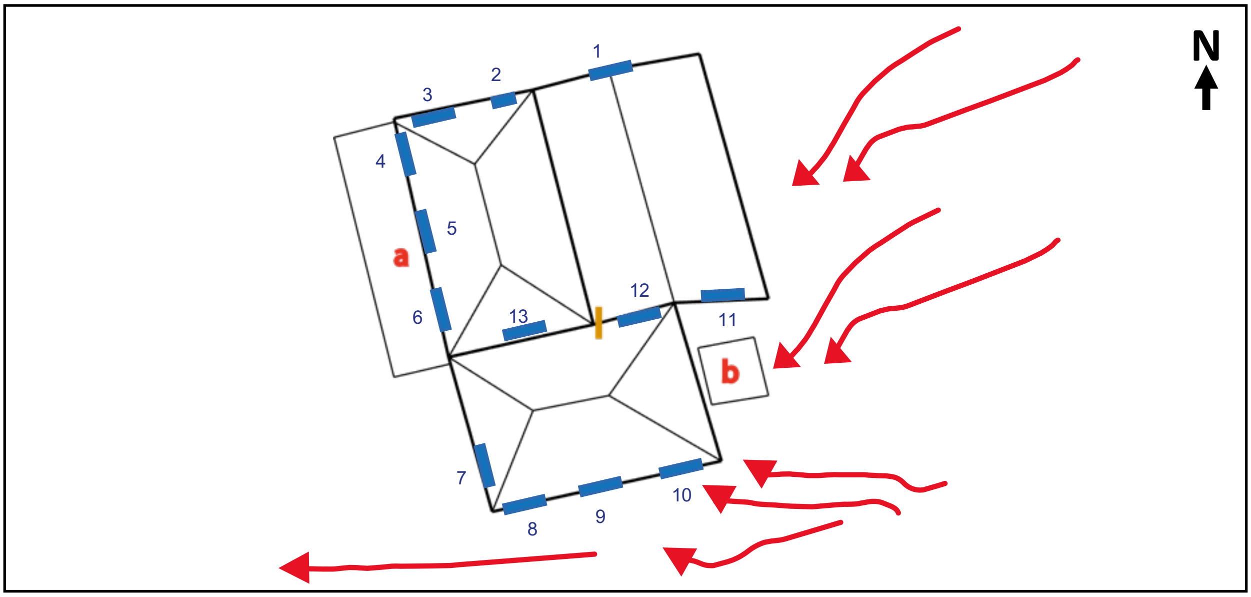

Type of structures in the property: I) Primary (main house) with accessory roofs (a, b)

N° Storeys: I) 1

Openings:
I) Main house/ First floor: - 1: 120 cm wide x 105 cm tall,- 2: 90 cm wide x 55 cm tall,- 3: 80 cm wide (door),- 4: 99 cm wide x 122 cm tall,- 5: 88 cm wide (door),- 6: 277 cm wide x 184 cm tall, - 7: 158 cm wide x 96 cm tall,- 8: 120 cm wide x 94 cm tall,- 9: 90 x 90 cm,- 10:,- 11: 119 cm wide x ~93 cm tall,- 12: 84 cm wide, - 13: 180 cm wide

Construction materials: I) AT0B1

Age of construction: New

Lava type: A'a

Lava thickness: m

Temporal order:

Damage: Fractures are observed in the walls, the top plate on the wall. The beams that support the ceiling and the tiles were made of wood and they are burned now. A'a lava entered the house through opening 8 and10 filling the room but stopped in the frame of opening 9.

30/10/2021:

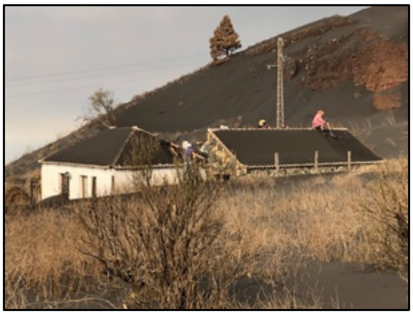

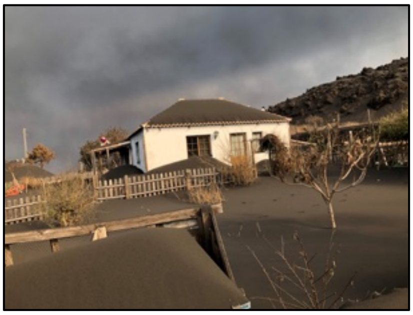

20/05/2022:

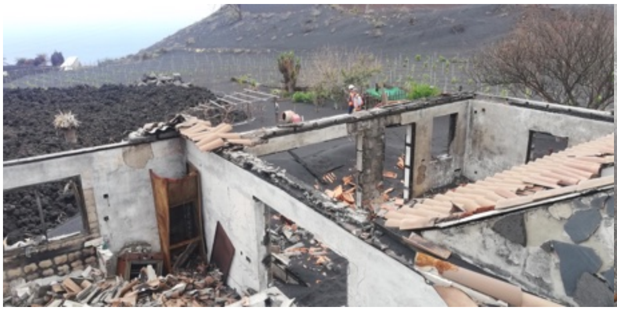

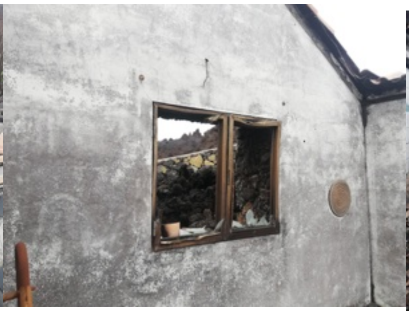

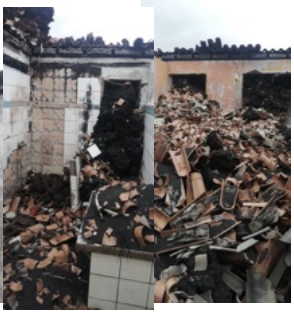

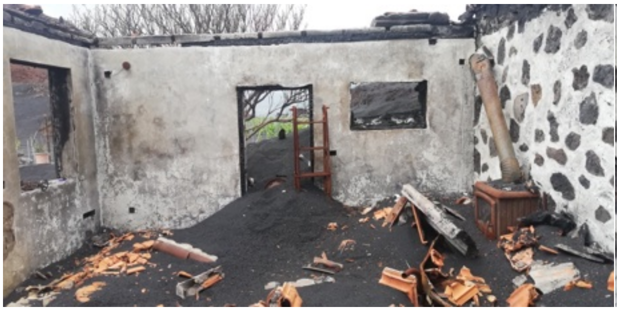

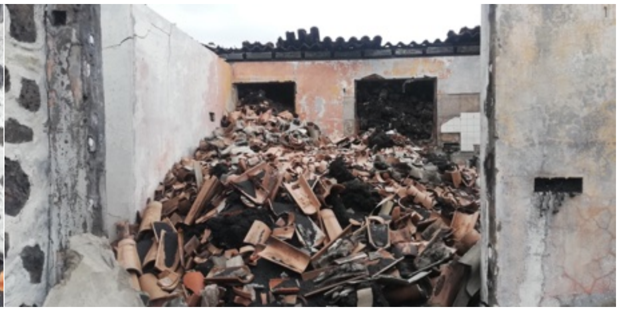

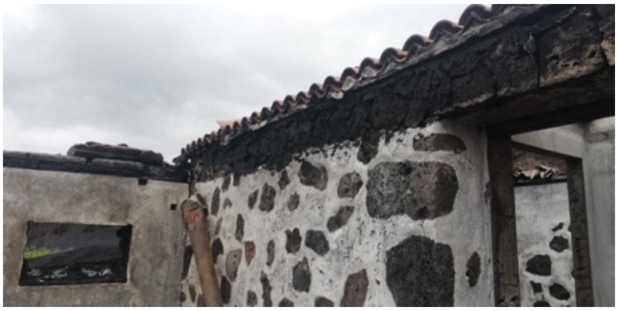

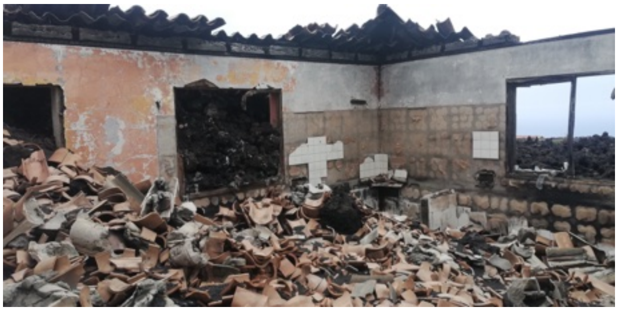

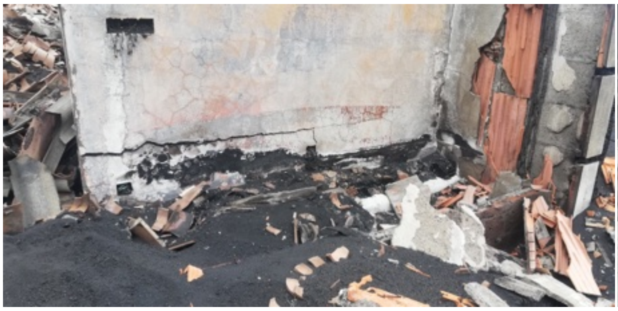

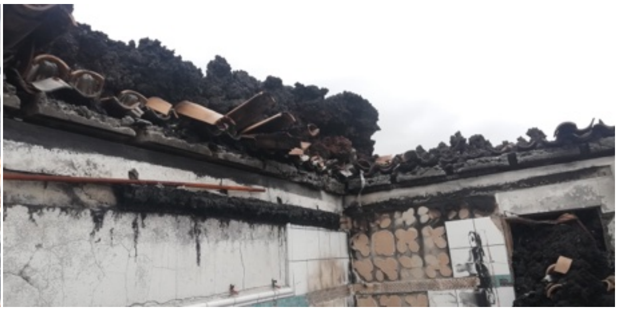

Name: DSLP-21 Damage State: 5
Location: 28.608901, -17.885993 Date: 20-05-2022

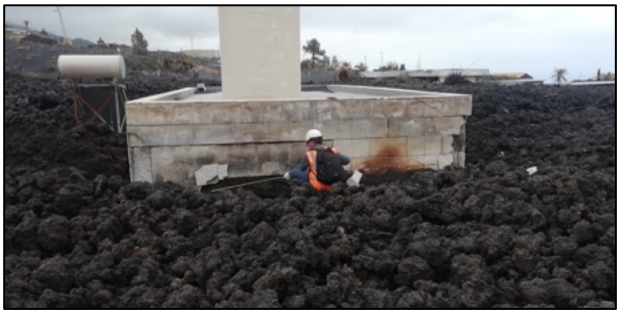

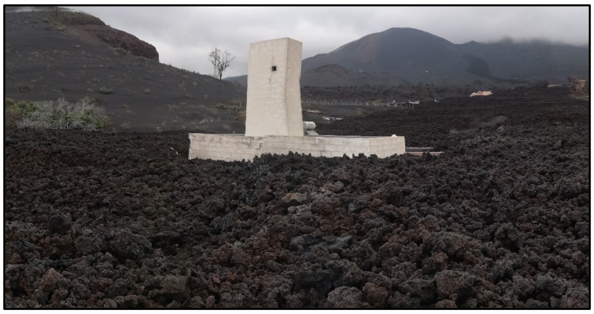

Footprint of the property:

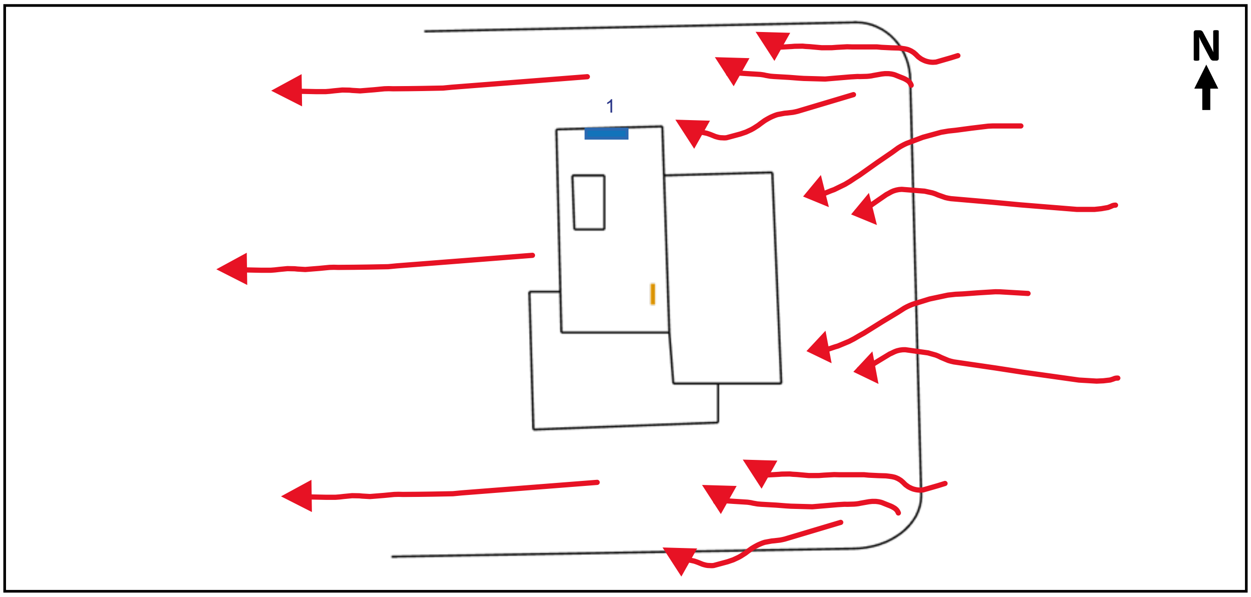

Type of structures in the property: I) crematorium cemetery of Las Manchas

N° Storeys: I) 1

Openings:
I) Main house/ First floor: - 1: 213 cm wide (window?)

* Between DSLP-21 and DSLP-22 we found a hole left by a palm tree (3.20 m deep) and a fissure where pahoehoe lava came out. The crack crosses the DSLP-22 structure at the NW corner and impacts the entire building.

Construction materials: I)FC0R1

Age of construction: New

Lava type: A'a

Lava thickness: m

Temporal order:

Damage: Lava A'a surrounds the entire building. The roof is bulging. The lava flow removed the tile facade that covered the building. The walls are made of reinforced concrete, some 1 cm diameter steel bars are observed in the broken corners of the building.

30/10/2021:

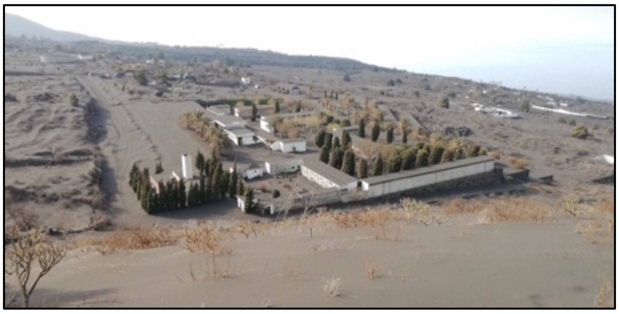

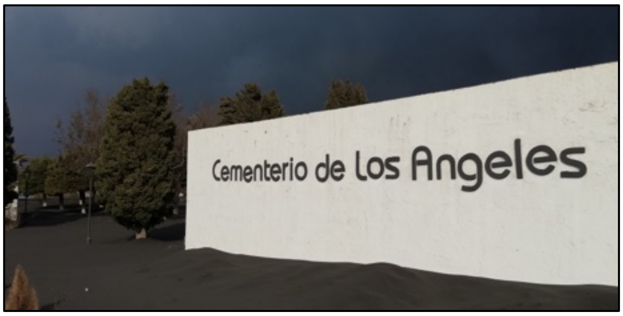

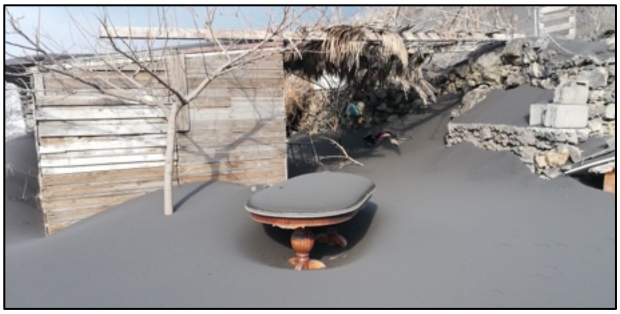

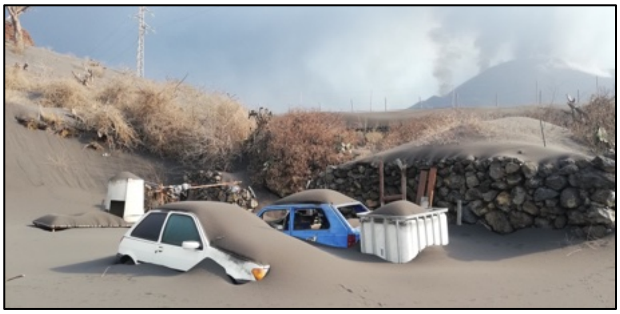

11/02/2022:

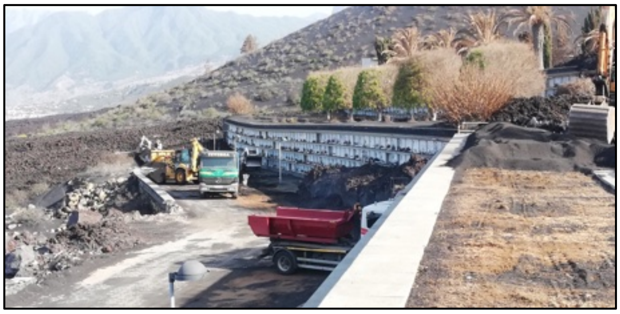

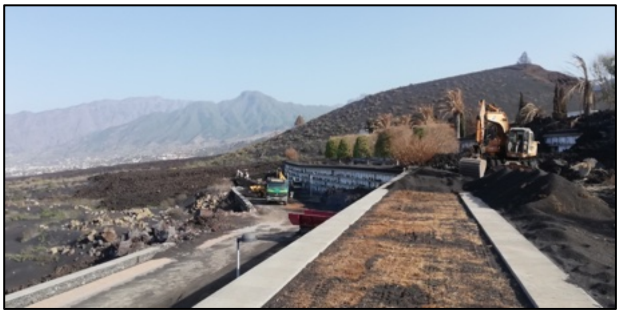

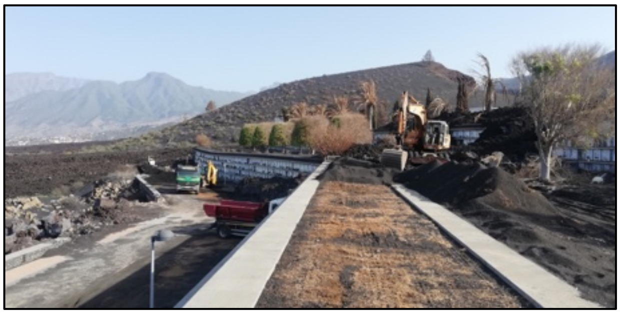

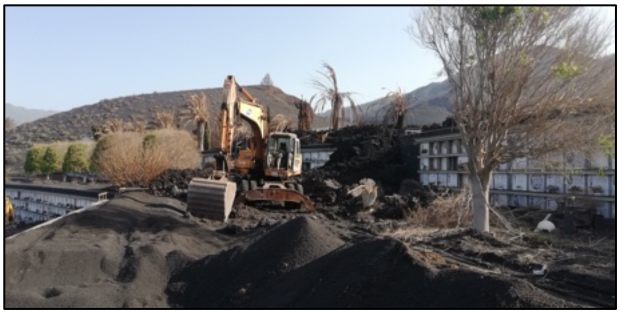

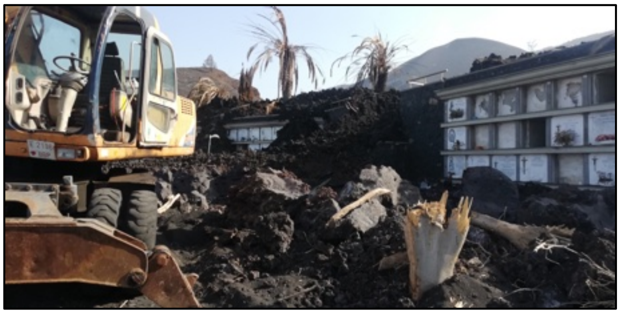

20/05/2022:

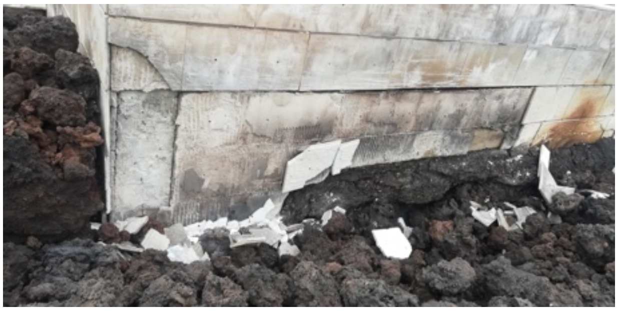

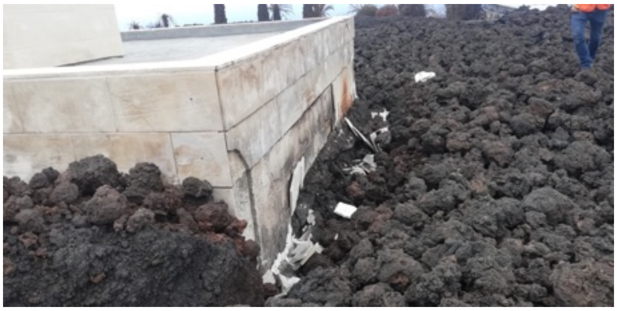

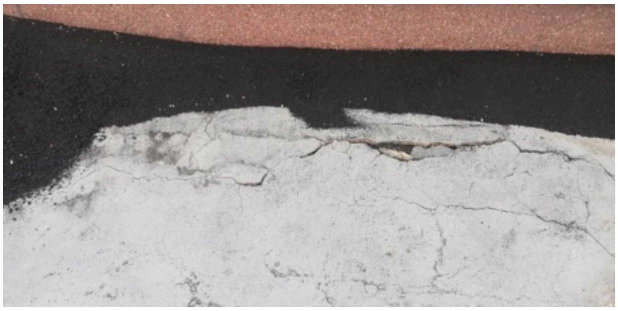

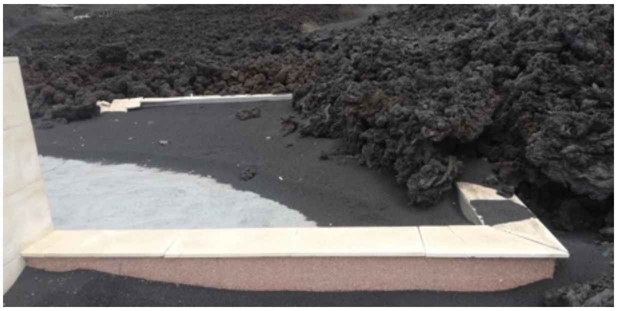

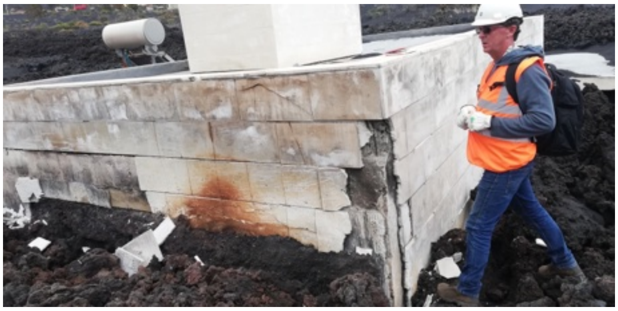

Name: DSLP-21/2 Damage State: 5
Location: 28.608355, -17.886395 Date: 20-05-2022

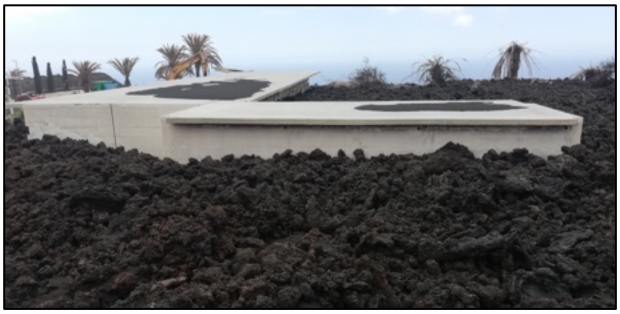
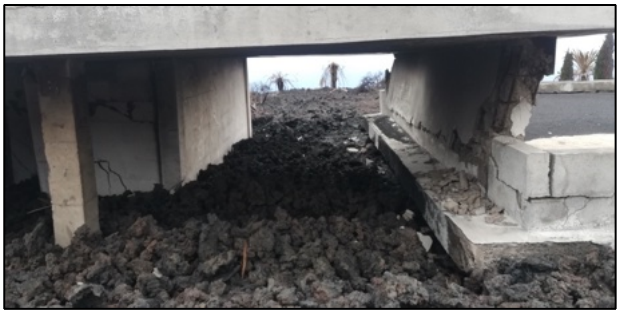

Footprint of the property:

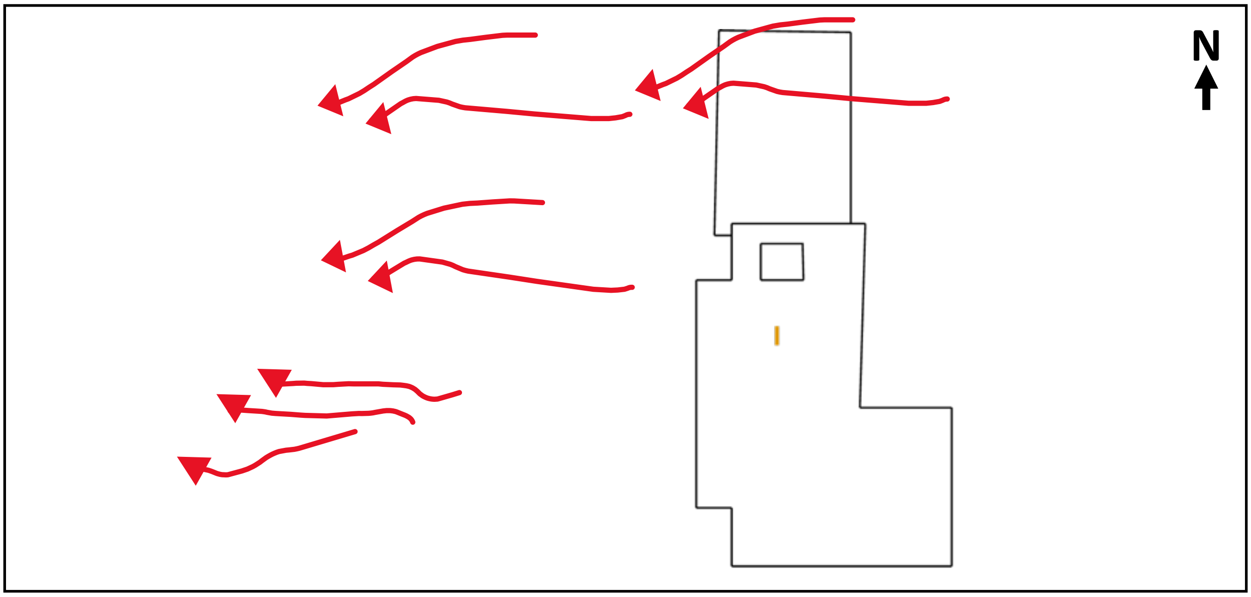

Type of structures in the property: I) cemetery of Las Manchas

N° Storeys: I) 1

Openings:
I) Main house/ First floor:

* Between DSLP-21 and DSLP-22 we found a hole left by a palm tree (3.20 m deep) and a fissure where pahoehoe lava came out. The crack crosses the DSLP-22 structure at the NW corner and impacts the entire building.

Construction materials: I)FC0R1

Age of construction: New

Lava type: A'a, pahoehoe

Lava thickness: m

Temporal order:

Damage: Structure made of blocks and reinforced concrete pillars. Fractures are observed throughout the building and the walls are inclined (9-10°). One of the pillars moved 16 cm to the left. Southern wall is displaced 2.5 cm from the pillar. The building looks burned inside.

20/05/2022:

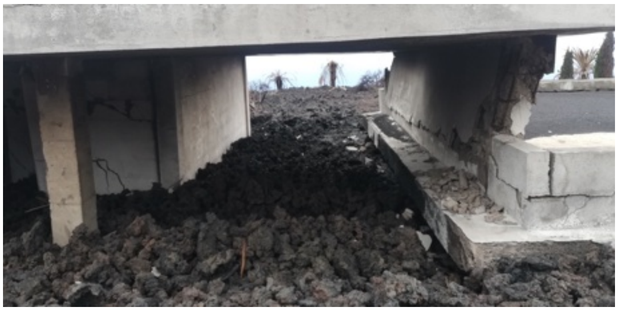

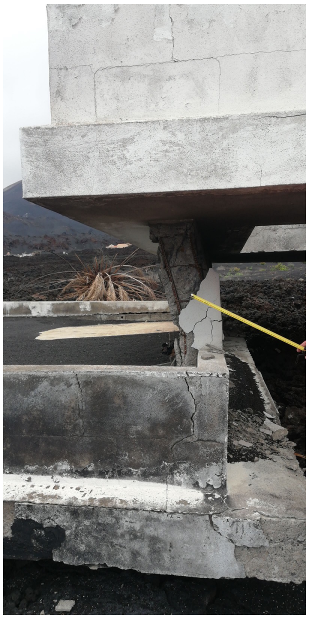

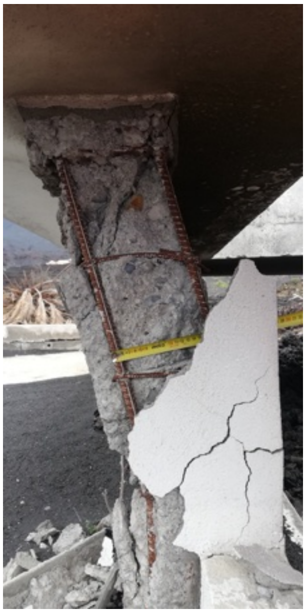

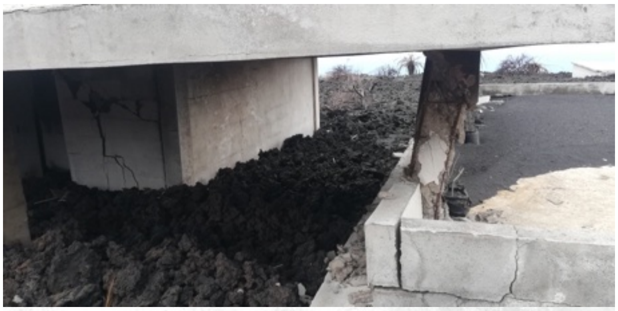

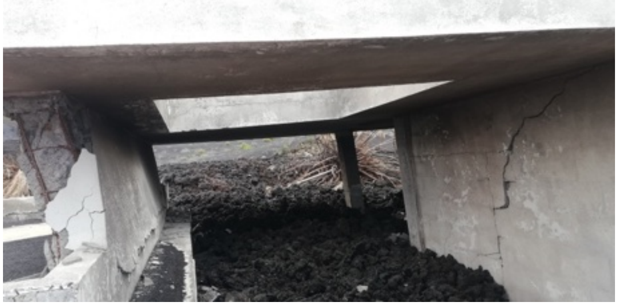

Supplement: Supplementary file 1 — Supplementary file1 (PDF 12752 KB) [file 445_2023_1700_MOESM1_ESM.pdf]
